# Thermal and UV-light dual-mode hydroboration of nitriles catalyzed by Mo(CO)_6_

**DOI:** 10.1039/d6ra04878a

**Published:** 2026-09-16

**Authors:** Shu-Fen Hou, Shijie Dong, Yujian Zhao, Haiyan Niu, Ailing Zhang, Mengyuan Zhang, Zaiyong Jiang, Kaoxue Li, Chunling Xin, Xiqi Ma

**Affiliations:** a College of Chemical Engineering and Environmental Chemistry, Weifang University Weifang 261061 P. R. China houshufen323@163.com; b China University of Petroleum East China Qingdao 266000 P. R. China

## Abstract

Molybdenum, an early-transition metal, exhibits both biological activity and catalytic properties. It displays unique advantages in the activation of organic molecules, particularly nitrogen-containing substrates. Herein, the simple organometallic carbonyl complex Mo(CO)_6_ was employed as a pre-catalyst for the hydroboration of nitriles. Under thermal and photocatalytic conditions, aliphatic, aromatic, and cyclic nitriles were transformed into diborylated amines with excellent chemoselectivity.

## Abstract 2

Mo(CO)_6_-catalyzed hydroboration of nitriles under dual thermal and UV-light activation.

## Graphical abstract

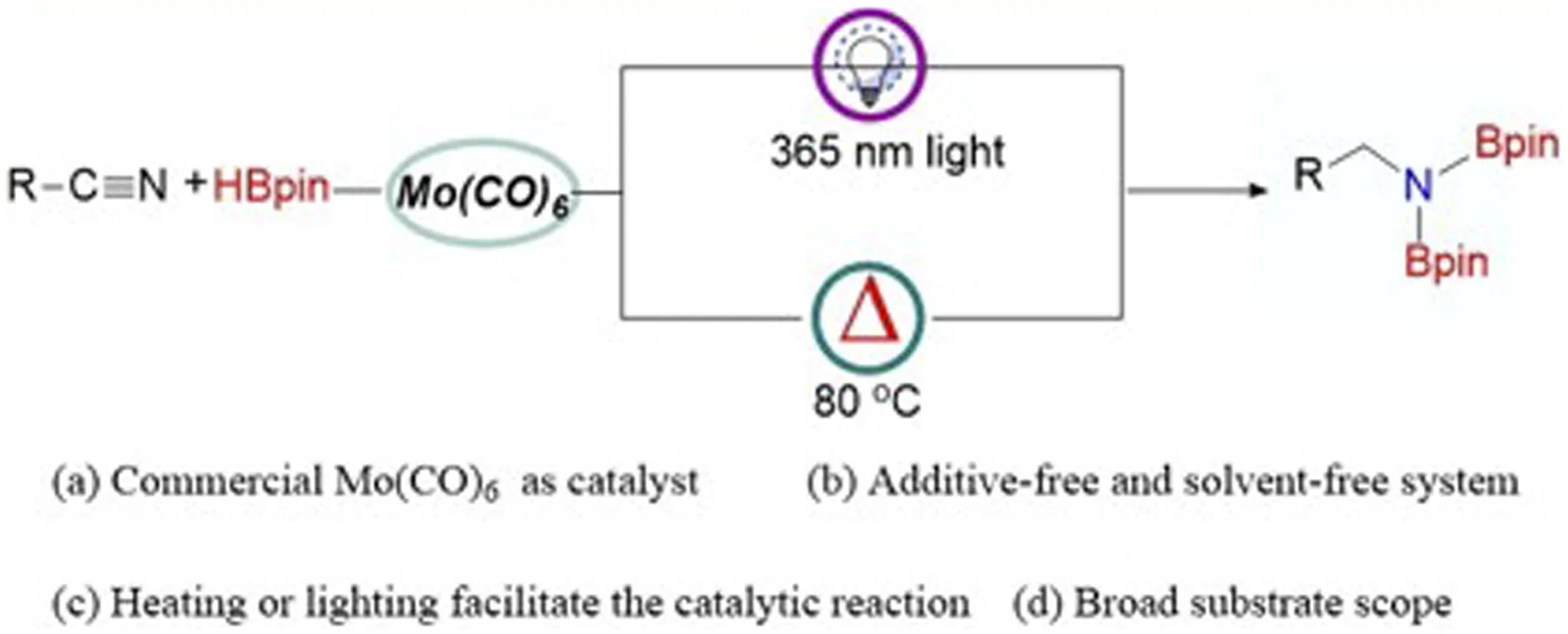

## Introduction

Nitriles exhibit a remarkably high C≡N bond dissociation energy (891.0 kJ mol^−1^), rendering them resistant to reduction under standard reaction conditions.^1^ Catalytic hydroboration provides a mild route for the reduction of nitriles.^2–4^ The hydroboration of nitriles catalyzed by late-transition metals (such as Ru, Ti, Fe, Co, Mn, Zn, Cu) has been extensively studied.^5–13^ Early-transition metals like Ti, Zr, W, and Mo are also promising owing to their versatile coordination chemistry. As shown in Scheme 1, Panda's group developed several Ti-based catalysts bearing N-donor or phosphine–borane ligands for hydrogenation of nitriles under mild conditions.^14–16^ Zr-based complexes capable of catalysing nitrile hydroboration were also described by the same team. Group 6 metal species have likewise found application as catalysts in nitrile hydroboration. Nikonov *et al.* disclosed a Mo^IV^ imido–hydrido complex (ArN)Mo(H)(Cl)(PMe_3_)_3_, which showed activity towards organic substrates such as ketones, esters, alkenes, and alkynes, yielding their respective hydroboration products.^17^ This complex also efficiently promoted the hydroboration of acetonitrile and benzonitrile with HBCat (catecholborane), generating bis(borylated) amines RCH_2_N(BCat)_2_. To date, this remains the only documented example of molybdenum-catalysed nitrile hydroboration. In 2024, Song and co-workers^2^ prepared W(CO)_4_(NCMe)_2_ from W(CO)_6_ and CH_3_CN, and utilised this complex as a hydroboration catalyst for nitriles and esters.

**Scheme 1:**
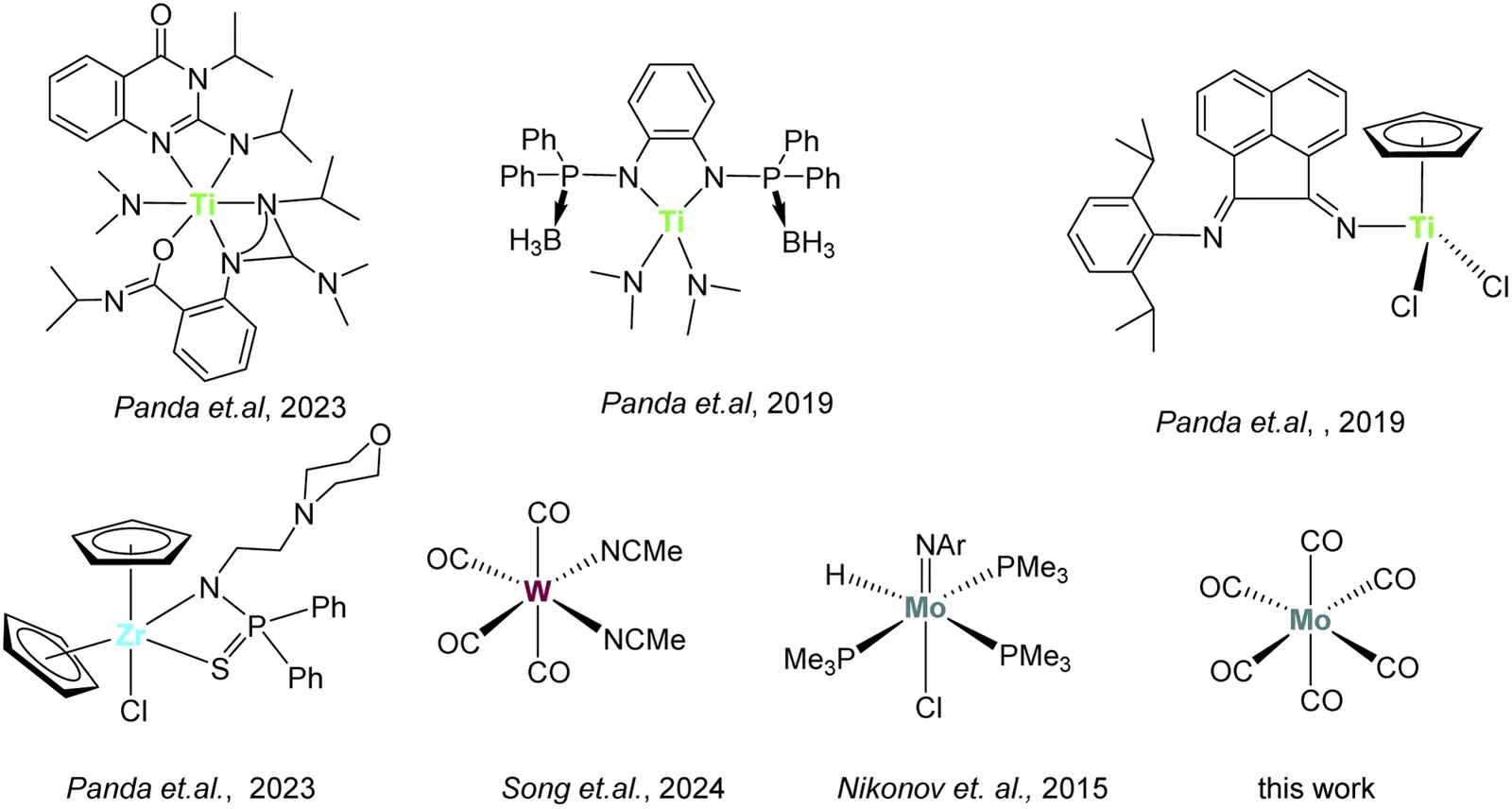
Selected examples of early-transition-metal catalysts for hydroboration reactions.

Early-transition-metal catalysts boast high natural abundance, low cost, low toxicity, and favourable biocompatibility. They feature tunable redox behaviour and high coordination numbers, display strong oxophilicity, and show excellent activity toward inert small-molecule activation. Their catalytic profiles are complementary to those of late-transition metals, rendering them highly promising for practical industrial deployment.^18,19^ As an early-transition metal, molybdenum plays an essential role in numerous biological processes and chemical cycles.^20–22^ It serves as a catalytic cofactor for a diversity of metalloenzymes, underpinning vital metabolic pathways across all domains of life, which accounts for its biological-evolutionary importance. Molybdenum exhibits strong Lewis acidity and high reactivity towards diverse organic substrates.^23–25^ Favorable elemental properties coupled with low toxicity endow Mo with both biological accessibility and high catalytic competence.^26–28^ High-valent molybdenum species, for example molybdenum oxides, are well-established catalysts for oxidation transformations.^29–31^ Notably, the Nobel-prize-winning molybdenum alkylidene complexes are prominent examples for alkene metathesis.^31,32^ Moreover, selected low-valent molybdenum compounds efficiently catalyse the reduction of unsaturated moieties.^33^ Molybdenum-based systems serve as homogeneous catalysts capable of mediating hydrogenation, transfer hydrogenation, hydrosilylation, hydroboration and hydrostannation.^34^ However, most of these Mo catalytic systems require ligand functionalization or external additives even when derived from commercially available molybdenum starting materials (Mo(CO)_6_ or MoX_*n*_Y_*m*_; X = Cl, Br, *n* = 3, 4, 5).

Our group has long focused on the chemistry of molybdenum-containing compounds. We have previously synthesized a suite of molybdenum complexes and demonstrated their utility in the hydrogenation of unsaturated substrates.^35,36^ Prior literature reveals that the preparation of many molybdenum catalysts relies on carbonyl-group dissociation from Mo(CO)_6_ triggered by heat or photoirradiation. Motivated by the reports from Baker^6^ and Song,^2^ herein we utilise Mo(CO)_6_ directly as a pre-catalyst for nitrile hydroboration under thermal or photochemical activation. Notably, this catalytic transformation tolerates ambient atmosphere and does not require rigorous anhydrous and oxygen-free handling; nitrile hydroboration can take place even in the presence of air.

## Results and discussion

We commenced our study by selecting 4-methylbenzonitrile (1b) as the model substrate (Table 1). Subjecting 1b to Mo(CO)_6_ (5 mol%) and HBpin (2.5 eq.) at 80 °C furnished the diborylated amine product (2b) with 99% conversion. Gratifyingly, ^1^H NMR and GC-MS analyses confirmed diborylated amine as the sole product, with no imine by-product detected. Temperature-dependent screening demonstrated that no hydroboration occurred at 30 °C, whereas 78% conversion was achieved at 50 °C and full conversion (99%) was observed at 80 °C. This trend is consistent with thermally driven carbonyl-ligand dissociation being essential for the activation of Mo(CO)_6_. Unlike many previously documented hydroboration protocols that demand N_2_-protected environments, our system exhibits good air tolerance. Carrying out the reaction under an air atmosphere instead of N_2_ led to only marginal yield loss (entry 4). We further assessed the moisture tolerance of this catalytic system.^37^ Introduction of 0.1 ml water into the reaction mixture resulted in a drastic drop of isolated yield to 20% (entry 5), indicating high water sensitivity. Solvent screening using THF revealed that optimal reactivity was achieved under solvent-free conditions.

**Table 1:** Optimization of reaction conditions for the hydroboration of 4-methylbenzenecarbonitrile^a^^,^^b^

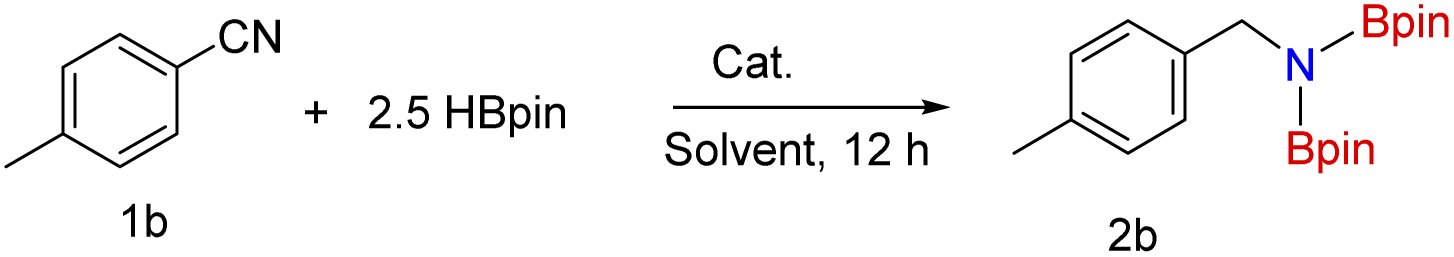				
Entry	Cat. (5 mmol%)	Solvent	*T* (°C)	Yield for 2b
1	Mo(CO)_6_	Neat	30	<5%
2	Mo(CO)_6_	Neat	50	78%
**3**	**Mo(CO)**_**6**_	**Neat**	**80**	**99%**
4^c^	Mo(CO)_6_	Neat	80	90%
5^d^	Mo(CO)_6_	Neat	80	20%
6	Mo(CO)_6_	THF	80	81%
7	W(CO)_6_	Neat	80	44%
8	Cr(CO)_6_	Neat	80	60%
9	Mo(acac)_2_	Neat	80	21%
10	MoO_2_	Neat	80	19%
11	Na₂MoO_4_	Neat	80	37%

a Conditions: 4-methylbenzonitrile (0.4 mmol), HBpin (1.0 mmol), pre-catalyst (5 mol%), 12 h.

b Yields determined by ^1^H NMR with tetraethylsilane as internal standard.

c Reaction under air.

d 0.1 ml H_2_O added.

Other group 6 metal carbonyl precursors (W(CO)_6_ and Cr(CO)_6_) were assayed at 80 °C in the absence of solvent, and both gave inferior yields compared with Mo(CO)_6_. High-valent molybdenum complexes MoO_2_ (Mo^IV^), Mo(acac)_2_ (Mo^IV^), and Na_2_MoO_4_ (Mo^VI^) also afforded relatively low product yields. Such reactivity disparities are plausibly ascribed to low-valent molybdenum centres possessing higher reactivity towards borane substrates.

Under the optimized reaction conditions, we explored the substrate scope across a broad range of nitriles, covering aliphatic derivatives as well as aromatic nitriles decorated with various electron-donating and electron-withdrawing moieties (Table 2). Among aromatic substrates, alkyl-substituted examples bearing electron-donating methyl (2b–2d) and methoxy (2j) groups delivered the target hydroboration products in good-to-excellent yields. Specifically, 2c and 2d were isolated in 96% and 94% yields, respectively, showing that sterically bulky methyl substituents impose only minor detrimental effects on this transformation. Consistent with this observation, the yield of 2k also implies that steric hindrance exerts negligible impact on product formation. Nitriles equipped with electron-withdrawing CF_3_- and halogen groups were efficiently accommodated, affording >90% product yields (2e–2i). High yields (>90%) were maintained for substrates carrying F, Cl, Br, and I halogen substituents. Furthermore, heterocyclic nitriles including furan-based (2n) and thiophene-based (2o) substrates were also compatible with this catalytic system.

**Table 2:** Hydroboration of diverse nitriles catalysed by Mo(CO)_6_ at 80 °C^a^

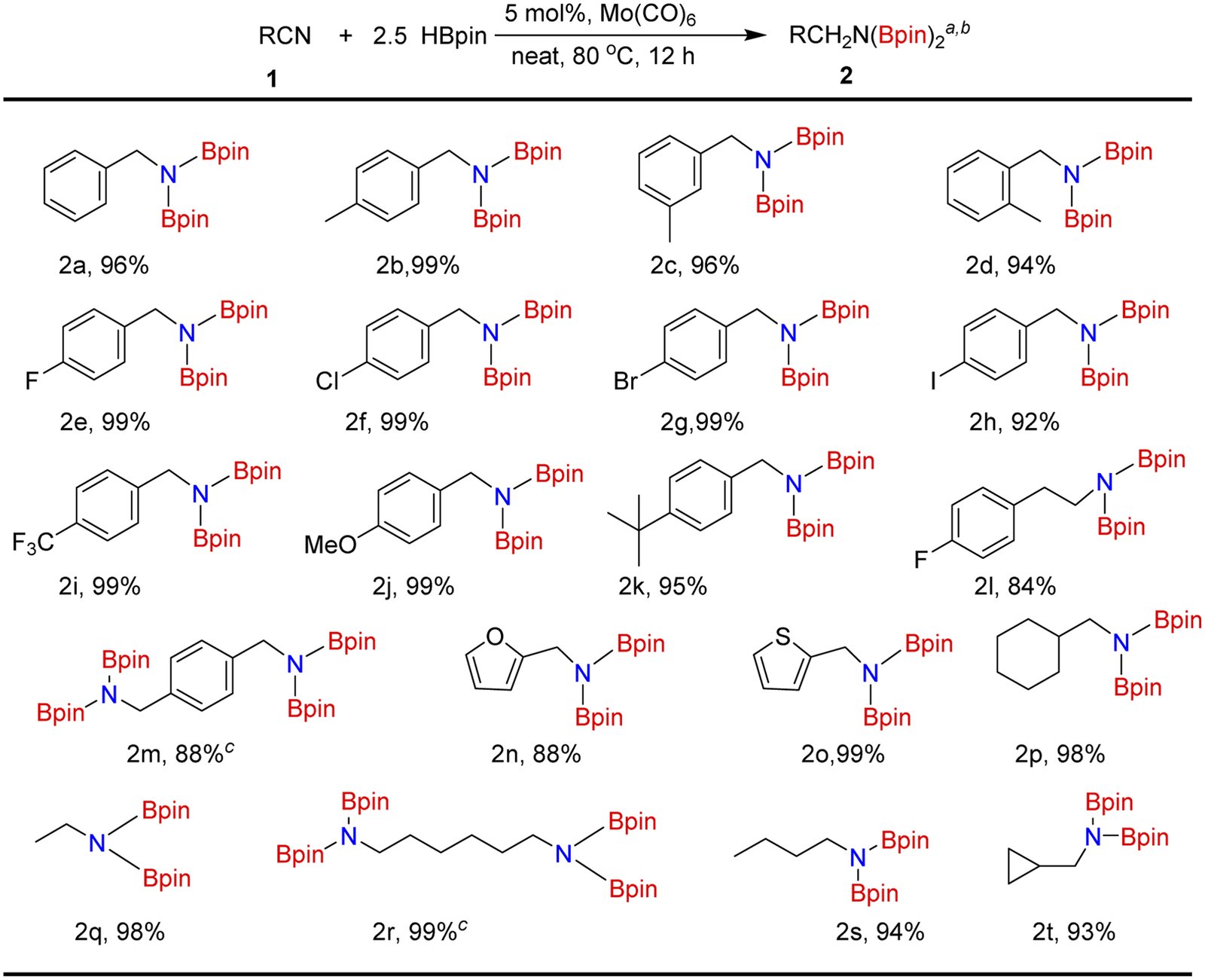

a Reaction conditions: nitriles (0.4 mmol), HBpin (1.0 mmol, 2.5 eq.), Mo(CO)_6_ (5 mol%), 80 °C, 12 h.

b Yields were determined by ^1^H NMR spectroscopy of the crude reaction mixture using tetraethylsilane as the internal standard.

c 5 eq. of HBpin and 10 mol% of Mo(CO)_6_ were used.

For aliphatic nitrile substrates, including acetonitrile (2q), butyronitrile (2s), and *p*-fluorobenzonitrile (2l), the corresponding hydroboration products were obtained in yields of 98%, 94%, and 84%, respectively. Cyclopropyl nitrile (2t) was also compatible with this catalytic system, delivering the target product in 93% yield without ring-opening of the three-membered cyclic structure. Furthermore, terephthalonitrile (1m) and adiponitrile (1r) were successfully transformed into their respective hydroboration products 2m and 2r, with isolated yields of 88% and 99%.

### Application of hydroboration reaction of nitriles

The diborylated species generated through this hydroboration protocol are versatile synthetic intermediates, which can be further transformed into a variety of nitrogen-containing functional compounds. These boron-functionalized products can react with aldehydes to construct N-substituted amide structures, and can also be hydrolyzed to obtain different amine derivatives. On the basis of the above results, we further explored the synthetic value of this catalytic system by carrying out a series of derivatization experiments (Scheme 2). The crude hydroboration mixture was directly treated with *p*-methylbenzaldehyde, and the target imine product was obtained with an NMR yield of 90%. In parallel, direct acid hydrolysis of the diborylated intermediate efficiently afforded the corresponding ammonium salt at 95% yield. Simple alkaline treatment of the resulting ammonium salt could further release the free amine product 4-methylbenzylamine, with an isolated yield of 80%. These experimental outcomes demonstrate that the Mo(CO)_6_-based catalytic system can serve as a reliable and practical approach for the modular synthesis of diverse amine derivatives.

**Scheme 2:**
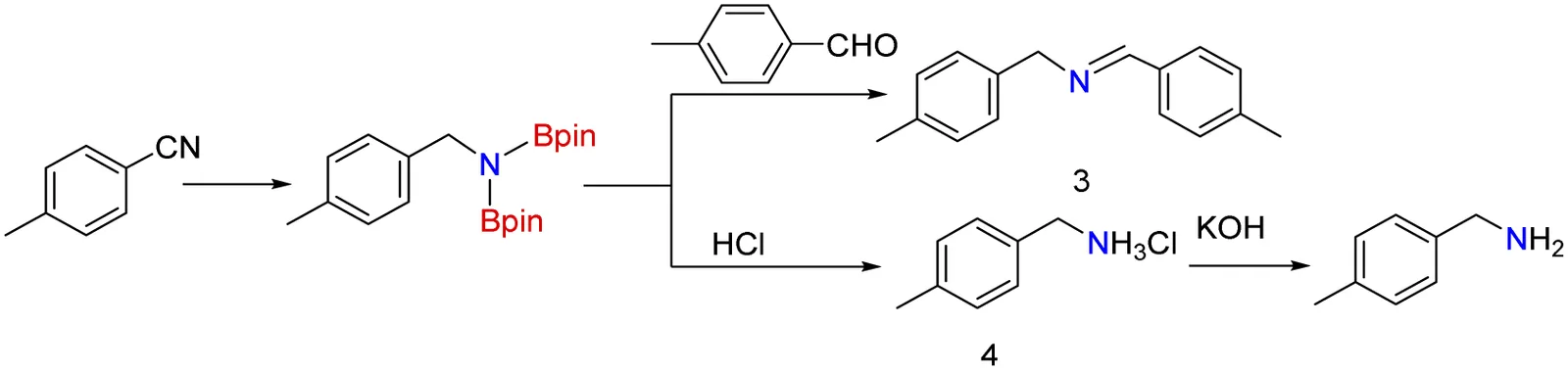
Application of hydroboration reaction of nitriles.

### Hydroboration of nitriles under UV light

Activation of Mo(CO)_6_ necessitates carbonyl-group dissociation. In our initial experiments, heating was utilised to induce this process. Aside from thermal activation, light irradiation represents a highly efficient means of triggering carbonyl dissociation. On this basis, we explored light-driven hydroboration of nitriles. 365 nm UV irradiation was chosen on the basis of spectroscopic observations and literature precedents. Mo(CO)_6_ shows weak absorbance at this wavelength, which enables gentle CO extrusion,^38–41^ whereas shorter-wavelength UV light causes unselective decomposition of both catalyst and substrate.^42^ Additionally, high substrate transparency, enhanced catalytic stability, and operational simplicity collectively validate 365 nm as the ideal irradiation wavelength for this nitrile hydroboration system. The hydroboration of 4-methylbenzonitrile was carried out under 365 nm UV irradiation, and the reaction mixture instantly turned yellow. A progressive colour deepening was observed over the course of the reaction. Following 8 h irradiation, ^1^H NMR analysis of the crude mixture revealed formation of the target hydroboration product in 99% yield. This result confirms that light is capable of promoting nitrile hydroboration.

A series of group VI metal carbonyl precatalysts were screened in the UV light mediated hydroboration of 4-methylbenzonitrile (Table 3). Increasing the loading of Mo(CO)_6_ from 1 mol% to 5 mol% led to a substantial rise in the yield of diborylated amine 2b, peaking at 99% under 5 mol% catalyst loading. Under these conditions, a TON of 20 and TOF of 2.5 h^−1^ were obtained. Lowering HBpin from 2.5 to 2 equivalents diminished product yield to 70%, pointing to the need for excess hydroborating reagent. We then probed the water compatibility of this photocatalytic hydroboration system. Addition of 10 mol% water reduced the product yield to 58%, indicating that water deactivates the molybdenum catalytic species. Parallel catalyst comparison revealed inferior reactivity for W(CO)_6_ (65% yield) and Cr(CO)_6_ (41% yield) relative to Mo(CO)_6_, establishing molybdenum hexacarbonyl as the most effective precatalyst for this transformation. Compared with W(CO)_6_ and Cr(CO)_6_, Mo(CO)_6_ has the lowest M–CO dissociation energy due to weak π-back donation and inter-ligand repulsion, so it readily generates unsaturated active sites;^43^ Mo has balanced HOMO energy,^44,45^ achieving fast electron transfer and facile site formation simultaneously;^41,46,47^ upon light irradiation, photolytic CO loss generates coordinatively unsaturated active species. Mo(CO)_6_ exhibits optimal photodissociation efficiency: Cr(CO)_6_ undergoes rapid over-photolysis forming inactive metal clusters, while W(CO)_6_ requires higher energy irradiation to trigger effective CO extrusion.^46,48^

**Table 3:** Optimization experiments for hydroboration of 4-methylbenzenecarbonitrile under UV light^a^^,^^b^

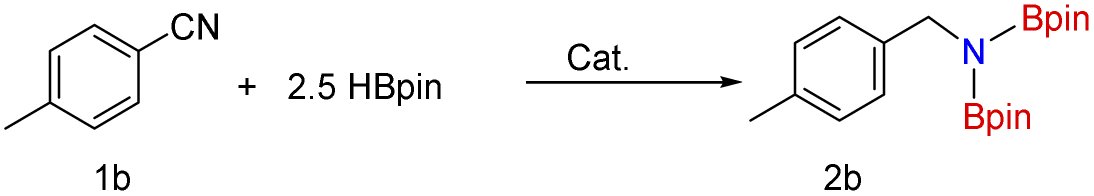			
Entry	Cat.	Catalyst loading	Yield for 2b
1	Mo(CO)_6_	1 mol%	65%
2	Mo(CO)_6_	2 mol%	80%
3	Mo(CO)_6_	5 mol%	99%
4^c^	Mo(CO)_6_	5 mol%	70%
5	W(CO)_6_	5 mol%	65%
6	Cr(CO)_6_	5 mol%	41%

a Reaction condition: nitriles (0.4 mmol), HBpin (1 mmol, 2.5 eq.), 365 nm light 30 W irradiation for 8 h.

b Yields were determined by ^1^H NMR spectroscopy of the crude reaction mixture using tetraethylsilane as an internal standard.

c HBpin (0.8 mmol, 2 eq.).

We next extended the optimized conditions to a range of nitrile substrates. Benzonitrile, 4-fluorobenzonitrile and (4-fluorophenyl)acetonitrile afforded the corresponding hydroboration products in 94%, 90% and 75% yield, respectively (Table 4).

**Table 4:** Select examples for hydrogenation of nitriles under UV light

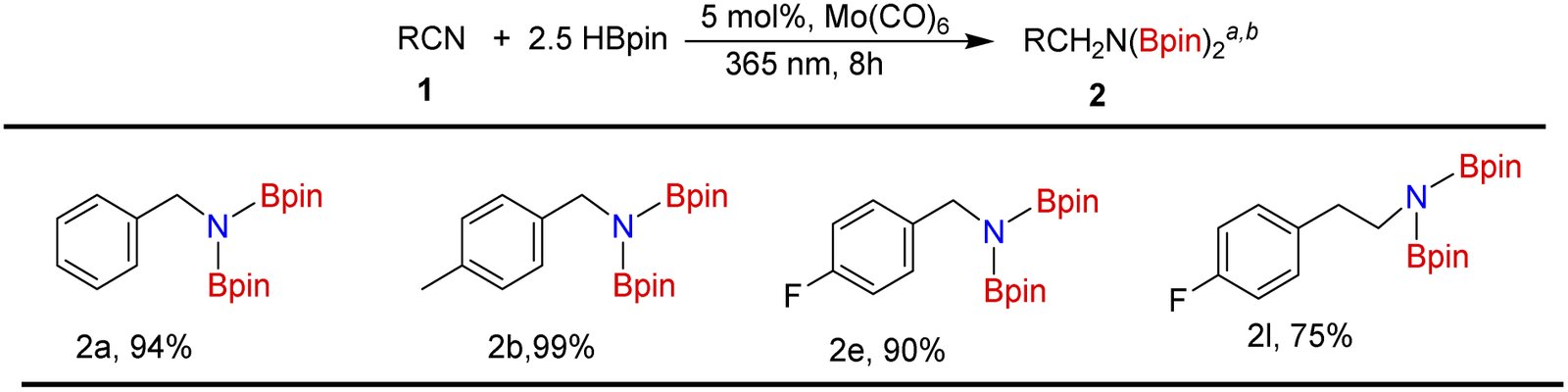

### Mechanistic investigations into UV-light-promoted nitrile hydroboration

A light-on/-off experiment was carried out to probe the role of light in the Mo(CO)_6_-catalysed photochemical diborylation of nitriles (RCN) with HBpin. Under standard conditions, substrate 1b was irradiated for 2h and product generation was monitored *via*^1^H NMR. Upon switching to dark conditions for an additional 2h, no further product accumulation was observed (Fig. 1A). Repeated light–dark alternations showed that product formation took place only under irradiation. These results establish that light is mandatory for the photochemical reaction channel. Nevertheless, 90% yield was attained at 80 °C in the absence of light, indicating that nitrile hydroboration may also proceed *via* a thermally activated manifold independent of photoexcitation.

**Fig. 1:**
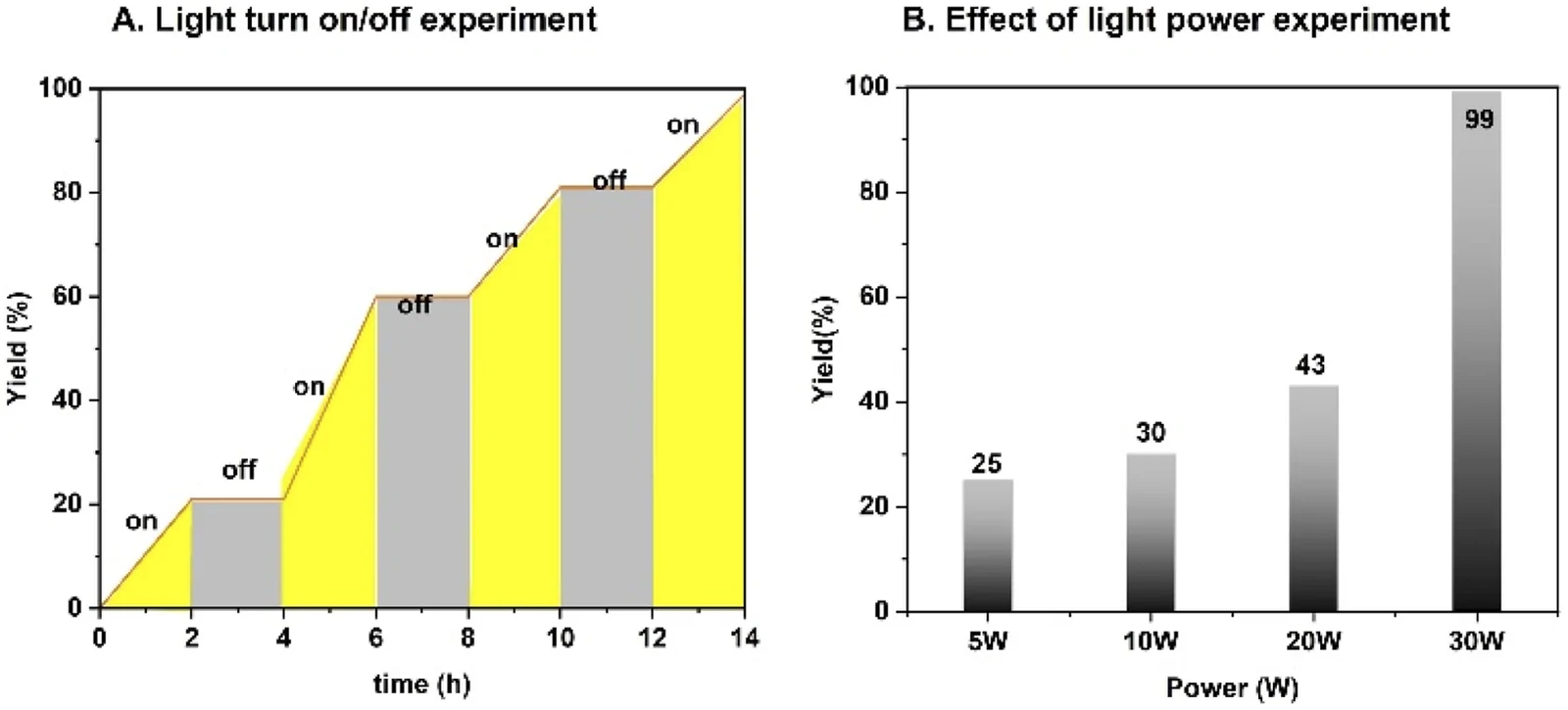
Mechanism studies of hydroboration of nitriles under UV light. (A) Light on/off experiment; (B) power control experiments.

Subsequently, we maintained a constant substrate concentration and evaluated the hydroboration performance under distinct light intensities (5 W, 10 W, 20 W, 30 W), with the corresponding product yields summarized in Fig. 1B. As illustrated in the figure, higher light intensity promotes the formation of the target product. Notably, this intensity-dependent effect is more prominent at low-power irradiation (5 W and 10 W), where the reaction only delivers product yields below 50%. Increasing the light power to 30 W significantly improved the reaction efficiency, affording an improved product yield of 99%. Collectively, these results confirm the photon-driven nature of this hydroboration reaction. Light intensity serves as a critical regulatory factor for the reaction process, whereby higher irradiation power accelerates the reaction rate and effectively facilitates product generation.

To further elucidate the role of light irradiation in this catalytic system, a pre-irradiation control experiment was performed (Scheme 3). Mo(CO)_6_ was first dissolved in tetrahydrofuran, and the resulting solution was irradiated under UV light for 2 h. Subsequently, HBpin and nitrile substrate were added sequentially, and the mixture was stirred in the dark for an additional 6 h. H NMR analysis revealed negligible product formation (less than 5%). This observation unambiguously demonstrates that *in situ* light irradiation is indispensable for triggering and sustaining the catalytic hydroboration reaction.

**Scheme 3:**
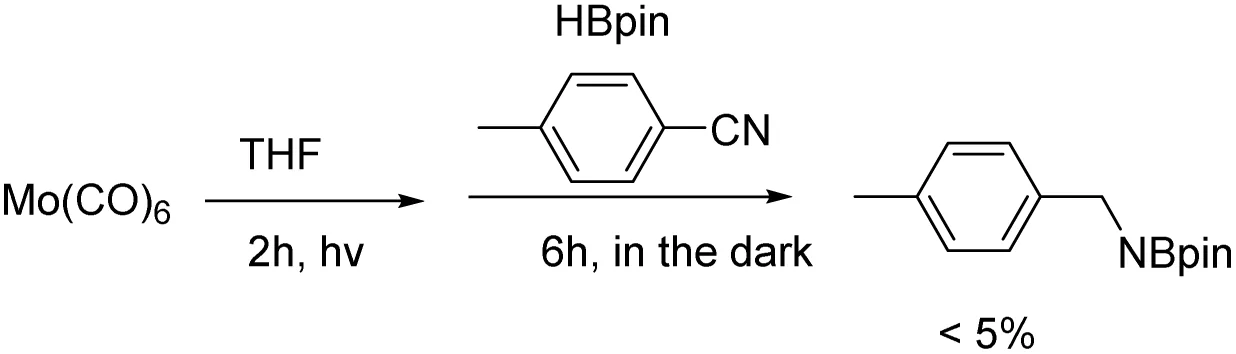
Study on the requirement of light for the reaction.

To further identify the nature of the catalytically active species, classic poisoning experiments with Hg, PPh_3_, and CS_2_ were carried out, and the corresponding results are summarized in the Table 5. Mercury addition barely suppressed the catalytic reaction, affording the target product in 97% yield. In sharp contrast, PPh_3_ induced gradual catalytic inhibition: the product yield decreased to 85% with 1 equiv. of PPh_3_, and further declined to 56% when the PPh_3_ dosage was increased to 5 equiv. Similarly, the introduction of CS_2_ led to moderate reaction suppression, with product yields dropping from 90% (0.5 equiv. CS_2_) to 70% (5 equiv. CS_2_).

**Table 5:** Poisoning experiments

Entry	Poisoning agent	Amount added	Yield for 2b	Conclusion
1	Hg	A drop of Hg	97%	Active species not Hg-poisoned
2	PPh_3_	1.0 equiv. (*vs.* Mo)	85%	Partial inhibition for cat.
3	PPh_3_	5.0 equiv. (*vs.* Mo)	56%	Inhibition by phosphine
4	CS_2_	1.0 equiv. (*vs.* Mo)	90%	Partial inhibition
5	CS_2_	5.0 equiv. (*vs.* Mo)	80%	Partial inhibition

As a classic scavenger for metallic nanoparticles, Hg is widely used to distinguish homogeneous and heterogeneous catalytic active sites. The negligible activity loss after Hg treatment strongly excludes heterogeneous molybdenum nanoparticles as the dominant active species in this system. The progressive inhibition caused by PPh_3_ can be attributed to its strong competitive coordination to coordinatively unsaturated molybdenum carbonyl fragments, which occupies the active sites and blocks substrate binding and activation. Meanwhile, the partial deactivation induced by CS_2_ further verifies the participation of unsaturated molecular molybdenum species in the catalytic cycle. The incomplete suppression of catalytic activity also indicates that the active molecular species cannot be fully sequestered under the tested experimental conditions.

To gain further insights into boron-containing species along the catalytic pathway, time-course ^11^B NMR monitoring experiments were performed under both photoirradiation and thermal conditions (Fig. S1). For the photo-driven reaction, aliquots were collected after 2 h, 4 h and 8 h of 365 nm UV-light irradiation. Similarly, parallel thermal reactions were sampled at 2 h, 4 h and 8 h under heating. In both series of spectra, two dominant resonances at *ca.* 24 ppm and 20 ppm are observed, which are assigned to the *N*,*N*-diborylamine product and HBpin, respectively. The intensity of the product resonance gradually increases with prolonged reaction time, accompanied by the gradual consumption of HBpin. Importantly, no signals assignable to borohydride-containing intermediates were detected either upon UV irradiation or during the heating process. These observations imply that any putative boron-hydride intermediate formed in the catalytic cycle is short-lived and undergoes rapid subsequent transformation, so that its steady-state concentration remains below the detection limit of *ex situ*^11^B{^1^H} NMR spectroscopy.

Mechanistic studies establish a dual photo-thermal activation process for this transformation. Thermal activation enables efficient nitrile hydroboration at 80 °C in the dark, while 365 nm UV light can mildly trigger CO photolysis to drive the reaction at room temperature. A series of control experiments, including light-on/off tests, light intensity gradient experiments, and pre-irradiation investigations, verified that the photochemical pathway is strictly light-dependent. Additional poisoning experiments confirmed that the catalytically active species are homogeneous unsaturated molybdenum carbonyl fragments, rather than heterogeneous metal nanoparticles.

## Conclusions

Herein, an efficient and facile Mo(CO)-catalyzed dihydroboration protocol for a wide range of nitriles was developed, which is accessible *via* both thermal and UV-light activation. Systematic catalytic screening confirmed that Mo(CO) exhibits uniquely superior reactivity among group VI metal carbonyls and high-valent molybdenum complexes. This catalytic system possesses remarkable synthetic advantages, including good air tolerance, broad substrate applicability covering aromatic, aliphatic, heterocyclic and dinitrile substrates, and high-yielding production of diverse diborylated amines. Furthermore, the obtained diborylated products can be readily transformed into valuable amines and amide derivatives, manifesting practical synthetic utility.

## Author contributions

Shu-Fen Hou: conceptualization, investigation, methodology, writing, funding acquisition, supervision. Shijie Dong, Yujian Zhao, and Haiyan Niu: investigation and data curation. Ailing Zhang: formal analysis. Mengyuan Zhang and Zaiyong Jiang: methodology and resources. Kaoxue Li: supervision and funding acquisition. Chunling Xin: visualization. Xiqi Ma: writing – review & editing. All authors have read and agreed to the published version of the manuscript.

## Conflicts of interest

There are no conflicts to declare.

## Supplementary Material

RA-016-D6RA04878A-s001

## Data Availability

The data underlying this study are available in the published article and its supplementary information (SI). Supplementary information is available. See DOI: https://doi.org/10.1039/d6ra04878a.
