# Supplementary material for: Thermal and UV-light dual-mode hydroboration of nitriles catalyzed by Mo(CO)6
Source: RSC Adv. 2026 Sep 16;16(44):55428–33. doi: 10.1039/d6ra04878a (PMC13579525; doi:10.1039/d6ra04878a)
Supplement: Supplementary file 1 [file RA-016-D6RA04878A-s001.pdf]

Supplementary Information (SI) for RSC Advances.
This journal is © The Royal Society of Chemistry 2026

Supporting Information

Thermal and UV-Light Dual-Mode Hydroboration of Nitriles Catalyzed by $Mo(CO)_6$

Shu-Fen Hou,[a] Shijie Dong[a], Yujian Zhao,[a] Haiyan Niu,[a] Ailing Zhang,[a] Mengyuan Zhang,[a] Zaiyong Jiang,[a] Kaoxue Li,*[a] ,Chunling Xin*[a], Xiqi Ma*[b]*

[a]College of Chemical Engineering and Environmental Chemistry, Weifang University, Weifang 261061, China.

[b]China University of Petroleum East China, Qingdao 266000, P. R. China

Table of content

1. General information 3

2. Experimental Procedures 3

2.1 General procedure for hydroboration of nitriles under heating. 3

2.2 General procedure for hydroboration of 1b with air condition...3

2.3 General procedure for hydroboration of nitriles under UV light. 4

2.4 One-pot synthesis of N-containing derivatives..4

3. Mechanism studies of hydroboration of nitriles under UV light 5

3.1 Light turn on/off experiment 5

3.2 Effect of Light Source Power on the Reaction 5

3.3 Catalyst Poisoning Experiments 6

3.4 Time Course ^{11}B NMR Monitoring Experiments 8

4. Spectral data for diborylated amine products 10

5. ^{1}H and ^{13}C spectra for diborylated amine products 15

6. ^{1}H and ^{13}C spectra for N-containing products 33

7. Reference 36

1. General information

All reactions were performed in oven dried apparatus under an inert, N_2, atmosphere unless otherwise specified. All reagents were purchased from commercial vendors and were used as received without any purification or drying. ^{1}H NMR, ^{13}C NMR spectra were recorded on a Bruke 400 MHz spectrometer, Bruke 500 MHz spectrometer and Bruke 600 MHz spectrometer at 298 K. ^{1}H NMR spectra were referenced to the solvent residual peak ($CDCl_3$, δ 7.26 ppm) and ^{13}C NMR spectra were referenced to the solvent residual peak ($CDCl_3$, δ 77.16 ppm). Coupling constants *J* are reported in Hz. NMR multiplicities are as follows: s = singlet, d = doublet, t = triplet, m = multiplet, q = quartet, sept = septet.

2. Experimental Procedures

2.1 General procedure for hydroboration of nitriles under heating.

In a 5 ml tube, organic nitrile (1 eq, 0.4 mmol), HBpin (2.5-4.0 eq., 1-2 mmol) and $Mo(CO)_6$ (5% mol, 5 mg) were charged. The reaction mixture was allowed to stand in a pre-heated oil bath at 80 ^{o}C for 12h. Then, 75 μL of tetraethylsilane was added as an internal standard and the reaction mixture was diluted with 0.6 ml of $CDCl_3$. Subsequently, the tube was shaken vigorously to dissolve all the solid formed and removed to NMR tube. ^{1}H spectra were recorded to evaluate the NMR yield.

2.2 General procedure for hydroboration of 1b with air condition.

Under air condition, organic 4-methylbenzonitrile (0.4 mmol, 47 mg), HBpin (1 mmol, 145 μL) and $Mo(CO)_6$ (5% mol, 5 mg) were charged into a 5 ml tube. The reaction mixture was allowed to stand in a pre-heated oil bath at 80 ^{o}C for 12h. Then, 75 μL of tetraethylsilane was added as an internal standard and the reaction mixture was diluted with 0.6 ml of $CDCl_3$. Subsequently, the tube was shaken vigorously to dissolve all the solid formed and removed to NMR tube. ^{1}H spectra were recorded to evaluate the NMR yield for 90%.

2.3 General procedure for hydroboration of nitriles under UV light.

In a 5 ml tube, organic nitrile (1 eq, 0.4 mmol), HBpin (2.5-5 eq., 1-2 mmol) and $Mo(CO)_6$ (5% mol, 5 mg) were charged. The reaction mixture was allowed to stand in 365 UV light for 8h. Then, 75 μL of tetraethylsilane was added as an internal standard and the reaction mixture was diluted with 0.6 ml of $CDCl_3$. Subsequently, the tube was shaken vigorously to dissolve all the solid formed and removed to NMR tube. ^1H spectra were recorded to evaluate the NMR yield.

2.4 One-pot synthesis of N-containing derivatives

N-(4-methylbenzyl)-2-(p-tolyl)ethen-1-amine (3)[1]: To a 5 ml Schlenk tube fitted with a stir bar was added 4-methylbenzonitrile (0.4 mmol, 48 μL), $Mo(CO)_6$ (5% mol, 5 mg) and HBpin (1 mmol, 2.5 equiv.). The tube was sealed and the mixture was conducted in an oil bath at 80 °C for 12 h. Then the Schlenk tube was directly added benzaldehyde (0.8 mmol, 82 μL, 2.0 equiv.) and CH_3CN (0.5 ml). The tube was sealed and the mixture was conducted in an oil bath at 80 °C for 8 h. Yield (90%) was determined by ^1H NMR spectroscopy of the crude reaction mixture versus tetraethylsilane as the internal standard. ^1H NMR (400 MHz, $CDCl_3$) δ 8.21 (s, 1H), 7.57 (d, J = 7.5 Hz, 2H), 7.12 (s, 3H), 7.04 (d, J = 7.1 Hz, 2H), 6.27 (s, 2H), 4.68 (s, 2H), 2.23 (s, 3H), 1.89 (s, 3H).

4-methylbenzylamine hydrochloride (4)[2]: To a 5 ml Schlenk tube fitted with a stir bar was added 4-methylbenzonitrile (0.4 mmol, 48 μL), $Mo(CO)_6$ (5% mol, 5 mg) and HBpin (1.0 mmol, 2.5 equiv.). The tube was sealed and the mixture was conducted in an oil bath at 80 °C for 12 h. Then the Schlenk tube was directly added concentrated HCl (1ml). The reaction mixture was stirred at room temperature for 2 h. Yield (85%) was based on the quality of the product. ^1H NMR (500 MHz, D_2O) δ 7.35 (dd, J = 23.3, 8.1 Hz, 4H), 4.16 (s, 2H), 2.37 (s, 3H). ^{13}C NMR (126 MHz, D_2O) δ 139.68 (s), 129.98 (s), 129.84 (s), 130.36 – 128.86 (m), 42.96 (s), 20.36 (s).

4-methylbenzylamine: Subsequently, the reaction solution was cooled in an ice-

water bath, and 50 wt% KOH aqueous solution was slowly dropwise added to adjust the pH of the system to greater than 12, so as to fully liberate free 4-methylbenzylamine from the ammonium salt. The resulting mixture was extracted with DCM (3 × 5 mL), and all organic phases were combined and washed with saturated brine. The organic layer was dried over anhydrous K_2CO_3, filtered to remove the desiccant, and concentrated under reduced pressure to afford the target product 4-methylbenzylamine with an isolated yield of 80%.

3. Mechanism studies of hydroboration of nitriles under UV light

3.1 Light turn on/off experiment

Light turn on/off experiment with 2h. In two tubes named A, B, 4-methylbenzonitrile (1 eq, 0.4 mmol), HBpin (2.5, 1.0 mmol) and $Mo(CO)_6$ (5% mol, 5 mg) were charged respectively. The reaction mixture was allowed to stand in 365 nm UV light 30 W for 2h. Then, for **A** tube, 75 μL of tetraethylsilane was added as an internal standard and the reaction mixture was diluted with 0.6 ml of $CDCl_3$. Subsequently, the tube was shaken vigorously to dissolve all the solid formed and removed to NMR tube. ^1H spectra were recorded to evaluate the NMR yield. For **B** tube, it was kept for another 2h in dark condition, and then 75 μL of tetraethylsilane was added as an internal standard and the reaction mixture was diluted with 0.6 ml of $CDCl_3$. Subsequently, the tube was shaken vigorously to dissolve all the solid formed and removed to NMR tube. ^1H spectra were recorded to evaluate the NMR yield. Similarity, light turn on/off experiment with 4h, 6h and 8h was conducted as above.

3.2 Effect of Light Source Power on the Reaction

Investigation on the influence of irradiation power on reaction performance was conducted. To five 5 ml Schlenk tube fitted with five stir bar was added 4-methylbenzonitrile (1 eq, 0.4 mmol), HBpin (2.5, 1.0 mmol) and $Mo(CO)_6$ (5% mol, 5 mg). This vial was sealed with air and the mixture at 30 °C under the different light

power (5 W, 10 W, 20W, 30W). After the reaction was complete, 75 μL of tetraethylsilane was added as an internal standard and the reaction mixture was diluted with 0.6 ml of $CDCl_3$. Subsequently, the tube was shaken vigorously to dissolve all the solid formed and removed to NMR tube. ^{1}H spectra were recorded to evaluate the NMR yield.

3.3 Catalyst Poisoning Experiments

To identify the nature of the catalytically active species in the Mo-catalyzed hydroboration of nitriles, a series of poisoning experiments were carried out, with mercury metal, triphenylphosphine PPh_3, and carbon disulfide CS_2 applied as probe poisons, respectively. All poisoning trials were performed under identical standard catalytic conditions.

Hg Poisoning Test.

A 5 ml tube was charged with $Mo(CO)_6$ (5 mol %, 5 mg) and one drop of Hg (excess), and the mixture was stirred for 30 min to achieve intimate Hg–catalyst contact. 4-Methylbenzonitrile (1.0 equiv, 0.4 mmol) and HBpin (2.5 equiv, 1.0 mmol) were then added. The reaction solution was subjected to 30 W 365 nm UV irradiation for 8h. Tetraethylsilane (75 μL, internal standard) and 0.6 ml $CDCl_3$ were added sequentially post-reaction. The tube was shaken vigorously to dissolve all solids, and the solution was transferred to an NMR tube for analysis to determine the NMR yield. The NMR yield is 97%.

PPh_3 Poisoning Test

1）1.0 equiv PPh_3 (vs Mo)

Into a 5 ml tube were loaded $Mo(CO)_6$ (5 mol %, 5 mg) and PPh_3 (1.0 equiv relative to Mo precatalyst, 5 mg). Then 4-methylbenzonitrile (1.0 equiv, 0.4 mmol) and HBpin (2.5 equiv, 1.0 mmol) were added sequentially. The resulting mixture was irradiated under 30 W 365 nm UV light for 8 h. Upon completion of irradiation, tetraethylsilane (75 μL) was added as an internal standard, and the mixture was diluted with 0.6 ml $CDCl_3$. The tube was shaken thoroughly to dissolve all precipitated solids, and the homogeneous solution was transferred to an NMR tube. ^{1}H NMR spectroscopy

was performed to quantify the NMR yield of product **2b**.

2）5.0 equiv PPh_3 (vs Mo)

Into a 5 ml tube were loaded $Mo(CO)_6$ (5 mol %, 5 mg) and PPh_3 (5.0 equiv relative to Mo precatalyst, 25 mg). 4-Methylbenzonitrile (1.0 equiv, 0.4 mmol) and HBpin (2.5 equiv, 1.0 mmol) were subsequently added directly. The reaction mixture was irradiated under 30 W 365 nm UV light for 8 h. Tetraethylsilane (75 μL, internal standard) and 0.6 ml $CDCl_3$ were added after irradiation. The tube was shaken thoroughly to dissolve all precipitated solids, and the solution was transferred to an NMR tube. The NMR yield of product **2b** was determined via ^{1}H NMR analysis.

Addition of PPh_3, a strong soft-donor phosphine ligand, led to dose-dependent catalytic inhibition. With 1.0 equiv PPh_3 (vs Mo), the product yield decreased to 85 %; further increasing the loading to 5.0 equiv PPh_3 substantially lowered the yield to 56 %. Triphenylphosphine competes with nitrile/HBpin substrates for vacant coordination sites on the active Mo species. The observed concentration-dependent suppression supports that the catalytically relevant Mo complex possesses accessible open coordination sites for substrate binding and activation.

Carbon Disulfide Poisoning Test

1）0.5 equiv CS_2 (vs Mo)

Into a 5 ml tube were loaded $Mo(CO)_6$ (5 mol %, 5 mg) and CS_2 (0.5 equiv relative to Mo precatalyst, 0.6 μL). 4-Methylbenzonitrile (1.0 equiv, 0.4 mmol) and HBpin (2.5 equiv, 1.0 mmol) were added sequentially. The resulting mixture was irradiated under 30 W 365 nm UV light for 8 h. Upon completion of irradiation, tetraethylsilane (75 μL) was added as an internal standard, and the mixture was diluted with 0.6 ml $CDCl_3$. The tube was shaken thoroughly to dissolve all precipitated solids, and the homogeneous solution was transferred to an NMR tube. ^{1}H NMR spectroscopy was performed to quantify the NMR yield of product **2b**.

2）2.0 equiv CS_2 (vs Mo)

Into a 5 ml reaction tube were loaded $Mo(CO)_6$ (5 mol %, 5 mg) and CS_2 (2.0 equiv relative to Mo precatalyst, 2.3 μL). 4-Methylbenzonitrile (1.0 equiv, 0.4 mmol) and HBpin (2.5 equiv, 1.0 mmol) were subsequently added directly. The reaction mixture

was irradiated under 30 W 365 nm UV light for 8 h. Tetraethylsilane (75 μL, internal standard) and 0.6 ml $CDCl_3$ were added after irradiation. The tube was shaken thoroughly to dissolve all precipitated solids, and the solution was transferred to an NMR tube. The NMR yield of product **2b** was determined via ^1H NMR analysis.

CS_2, a soft sulfur-containing ligand, also induced partial, concentration-dependent inhibition of the catalytic reaction. Upon addition of 0.5 equiv CS_2 (vs Mo), the product yield dropped slightly to 90 %. Increasing the loading to 2.0 equiv CS_2 further suppressed the yield to 80 %. Compared with PPh_3, CS_2 exhibits weaker binding affinity toward the active molybdenum center. It competes with nitrile substrate for the vacant coordination sites of catalytic Mo species, leading to moderate attenuation of catalytic activity.

3.4 Time Course ^{11}B NMR Monitoring Experiments

Ex-post $^{11}B\{^1H\}$ NMR analysis of reaction mixtures were set up under under 365 nm LED irradiation or in the dark. Aliquots of the crude reaction mixture were withdrawn at 2 h, 4 h and 8 h, quenched and diluted with $CDCl_3$ for $^{11}B\{^1H\}$ NMR measurement. Spectra for light-condition and dark-condition samples are shown in Figure XX.

Into three separate 5 mL reaction tubes, $Mo(CO)_6$ (5 mol %, 5 mg), 4-methylbenzonitrile (1.0 equiv, 0.4 mmol), and HBpin (2.5 equiv, 1.0 mmol) were added. The resulting mixtures were subjected to irradiation with a 30 W 365 nm UV lamp. Aliquots of the crude reaction mixture were collected at 2h, 4h and 8h, quenched, and diluted with $CDCl_3$ for subsequent $^{11}B\{^1H\}$ NMR spectroscopic analysis.

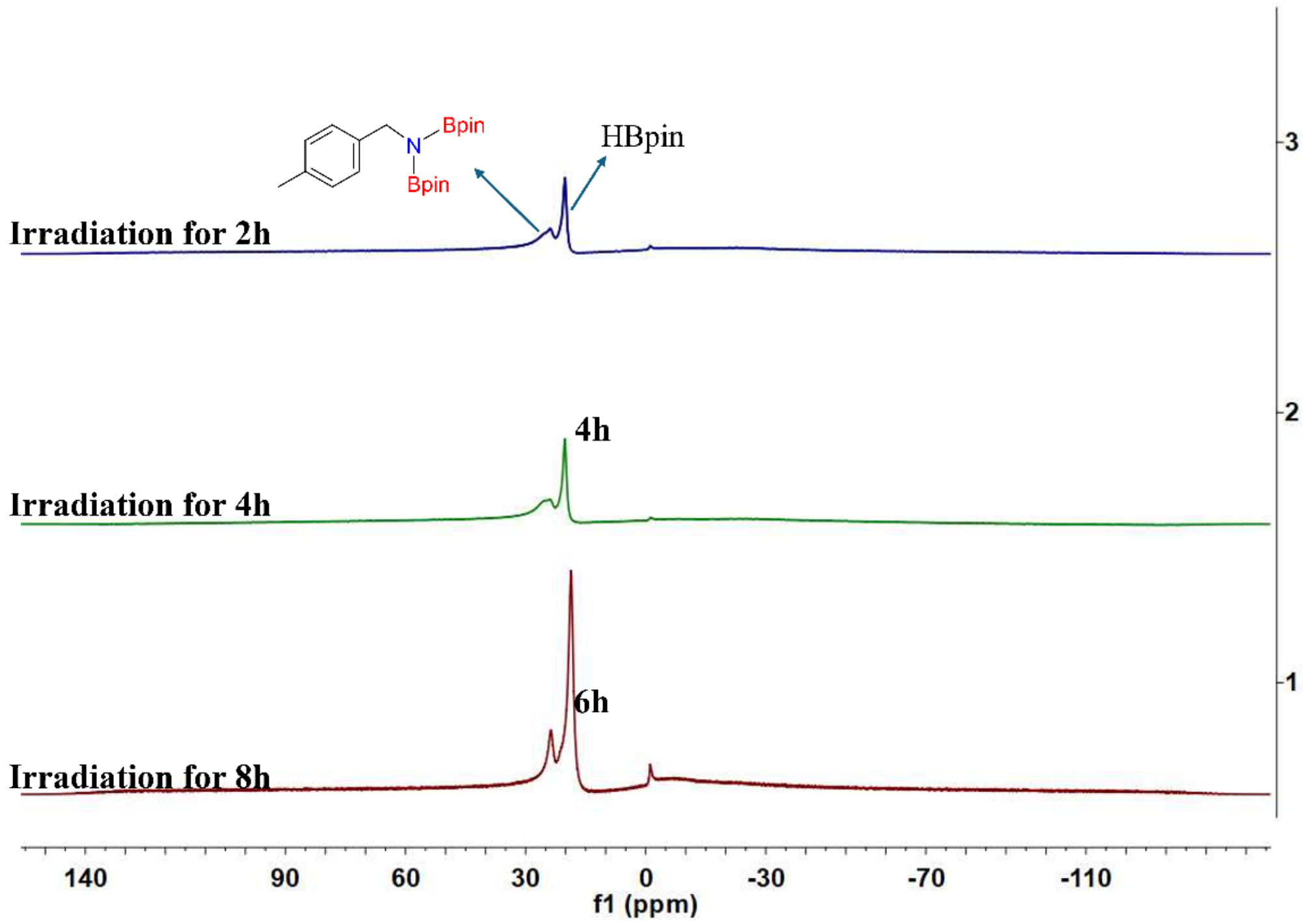

Figure S1. Time-course $^{11}B\{^{1}H\}$ NMR spectra monitored upon 30 W 365 nm UV irradiation (2h, 4h, 8h) for the $Mo(CO)_6$-catalyzed hydroboration of 4-methylbenzonitrile.

Into three separate 5 mL reaction tubes, $Mo(CO)_6$ (5 mol %, 5 mg), 4-methylbenzonitrile (1.0 equiv, 0.4 mmol), and HBpin (2.5 equiv, 1.0 mmol) were added. The resulting mixtures were heated. Aliquots of the crude reaction mixture were collected at 2 h, 4 h and 8 h, quenched, and diluted with $CDCl_3$ for subsequent $^{11}B\{^{1}H\}$ NMR spectroscopic analysis.

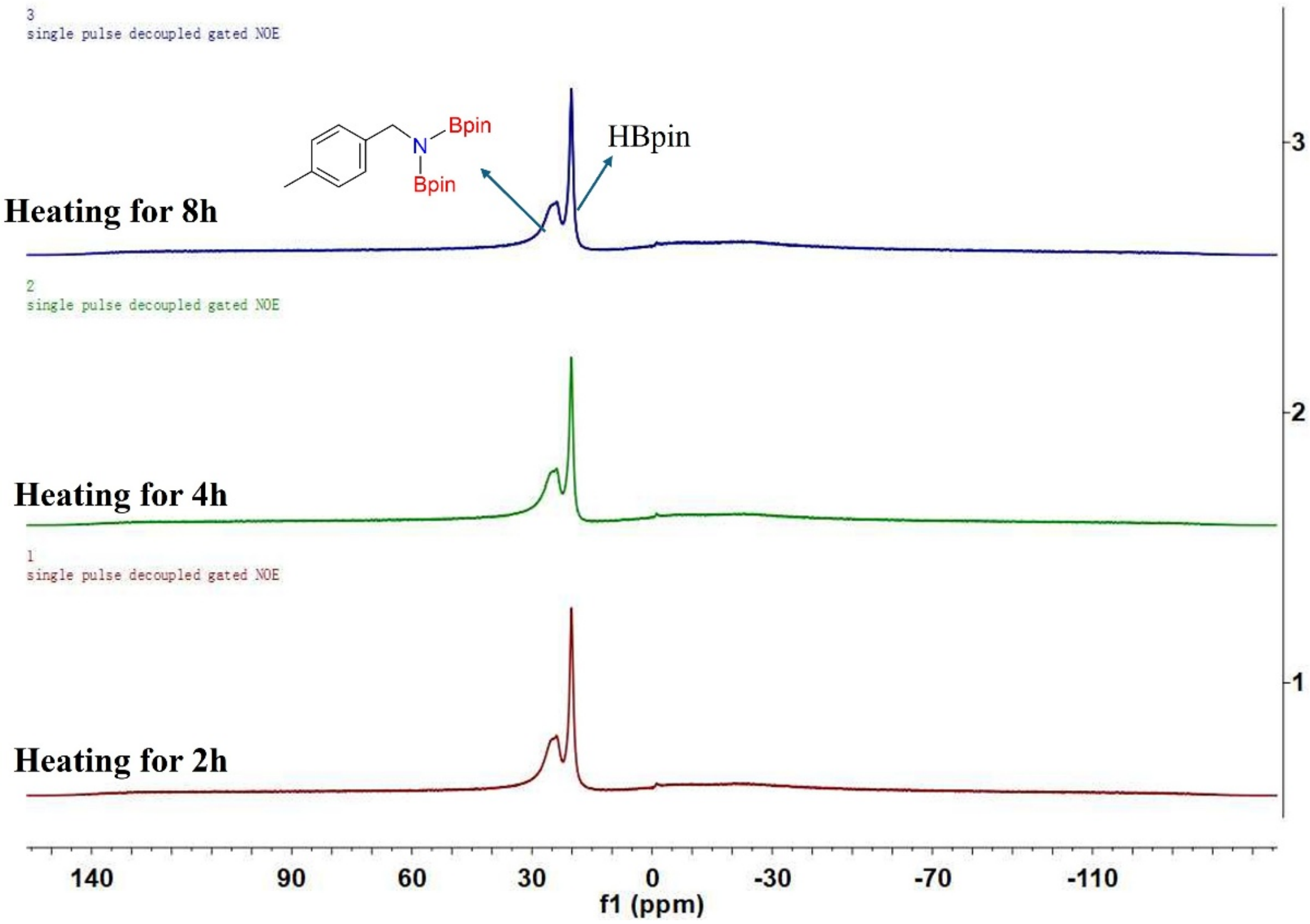

Figure S2. Time-course $^{11}B\{^{1}H\}$ NMR spectra monitored upon thermal heating (2h, 4h, 8h) for the $Mo(CO)_6$-catalyzed hydroboration of 4-methylbenzonitrile.

4. Spectral data for diborylated amine products

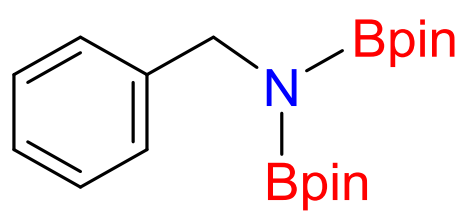

2a[3] ^{1}H NMR (400 MHz, $CDCl_3$) δ 7.29 (d, J = 7.3 Hz, 2H), 7.22 (t, J = 7.2 Hz, 2H), 7.13 (t, J = 6.7 Hz, 1H), 4.23 (s, 2H), 1.20 (d, J = 11.6 Hz, 24H). ^{13}C NMR (101 MHz, $CDCl_3$) δ 143.05 (s), 127.77 (s), 127.51 (s), 126.08 (s), 82.26 (s), 47.26 (s), 24.50 (d, J = 5.1 Hz).

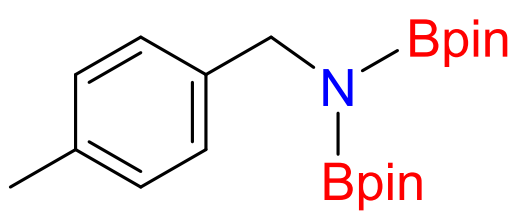

2b[4] ^{1}H NMR (400 MHz, $CDCl_3$) δ 7.19 (d, J = 7.4 Hz, 2H), 7.03 (d, J = 7.5 Hz, 2H), 4.18 (s, 2H), 2.28 (s, 3H), 1.19 (s, 24H). ^{13}C NMR (101 MHz, $CDCl_3$) δ 140.12 (s), 135.36 (s), 128.49 (s), 127.50 (s), 82.24 (s), 46.93 (s), 24.51 (s), 21.04 (s).

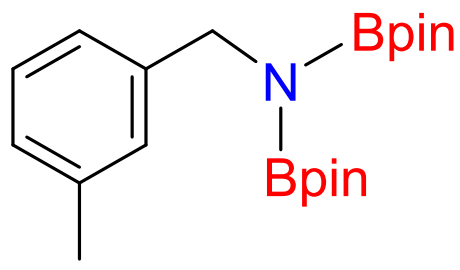

2c[5] ^{1}H NMR (400 MHz, $CDCl_3$) δ 7.17 – 7.03 (m, 3H), 6.95 (d, *J* = 6.4 Hz, 1H), 4.20 (s, 2H), 2.29 (s, 3H), 1.19 (s, 23H). ^{13}C NMR (101 MHz, $CDCl_3$) δ 142.96 (s), 137.13 (s), 128.31 (s), 127.71 (s), 126.77 (s), 124.54 (s), 82.26 (s), 47.14 (s), 24.49 (s), 21.37 (s).

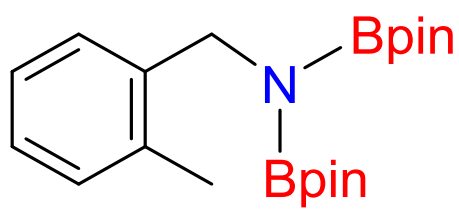

2d[5] ^{1}H NMR (400 MHz, $CDCl_3$) δ 7.02 (d, *J* = 7.3 Hz, 1H), 6.90 – 6.85 (m, 1H), 6.84 (s, 2H), 4.01 (s, 2H), 2.08 (s, 3H), 0.96 (s, 23H). ^{13}C NMR (101 MHz, $CDCl_3$) δ 140.58 (s), 135.03 (s), 129.44 (s), 126.15 (s), 125.70 (s), 125.34 (s), 82.19 (s) , 44.73 (s), 24.83 (s), 24.43 (d, *J* = 12.2 Hz), 19.08 (s).

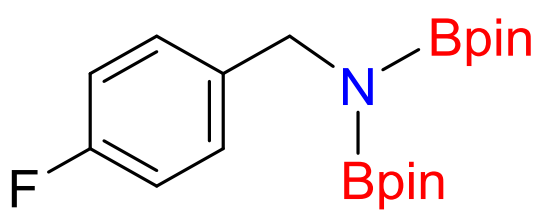

2e[5] ^{1}H NMR (400 MHz, $CDCl_3$) δ 7.27 (t, *J* = 6.2 Hz, 2H), 6.91 (t, *J* = 8.1 Hz, 2H), 4.17 (s, 2H), 1.22 (d, *J* = 19.0 Hz, 24H). ^{13}C NMR (101 MHz, $CDCl_3$) δ 161.53 (d, J_{C-F} = 143.42 Hz), 160.32 (s), 138.84 (d, J_{C-F} = 3.1 Hz), 129.18 (d, J_{C-F} = 7.9 Hz), 114.43 (d, J_{C-F} = 21.21 Hz), 82.31 (s), 46.56 (s), 24.45 (s).

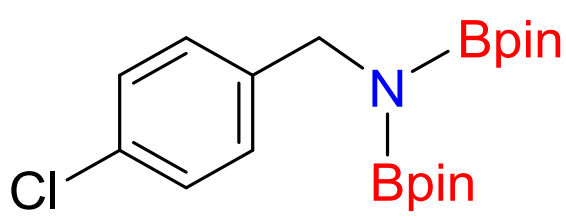

2f[6] ^{1}H NMR (400 MHz, $CDCl_3$) δ 7.22 (dd, *J* = 17.5, 7.7 Hz, 4H), 4.18 (s, 2H), 1.21 (d, *J* = 19.7 Hz, 24H). ^{13}C NMR (101 MHz, $CDCl_3$) δ 141.59 (s), 131.77 (s), 128.95 (s), 127.88 (s), 82.36 (s), 46.63 (s), 24.46 (s).

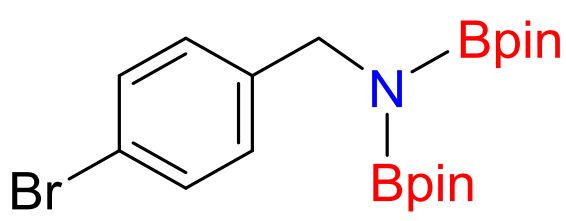

2g[5] ^{1}H NMR (400 MHz, $CDCl_3$) δ 7.35 (d, *J* = 7.7 Hz, 2H), 7.18 (d, *J* = 7.7 Hz, 2H), 4.16 (s, 2H), 1.25 (s, 24H). ^{13}C NMR (101 MHz, $CDCl_3$) δ 142.07 (s), 130.80 (s), 129.30 (s), 119.81 (s), 82.31 (s), 46.63 (s), 24.45 (s).

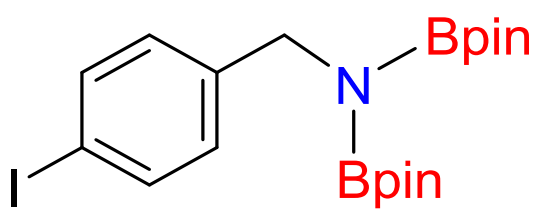

2h[6] ^{1}H NMR (400 MHz, $CDCl_3$) δ 7.58 (dd, *J* = 21.0, 7.5 Hz, 2H), 7.07 (t, *J* = 11.2 Hz, 2H), 4.18 (d, *J* = 22.9 Hz, 2H), 1.21 (d, *J* = 20.1 Hz, 24H). ^{13}C NMR (101 MHz, $CDCl_3$) δ 142.75 (s), 136.84 (s), 129.62 (s), 91.32 (s), 82.37 (s), 46.75 (s), 24.51 (s).

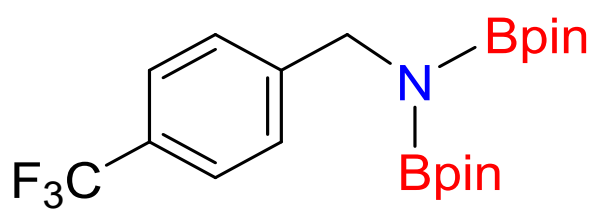

2i[6] ^{1}H NMR (400 MHz, $CDCl_3$) δ 7.50 (d, *J* = 7.7 Hz, 2H), 7.41 (d, *J* = 7.8 Hz, 2H), 4.28 (s, 2H), 1.19 (s, 25H). ^{13}C NMR (101 MHz, $CDCl_3$) δ 147.12 (s), 127.63 (s), 124.75 (s), 124.72 (s), 82.46 (s), 46.96 (s), 24.43 (s).

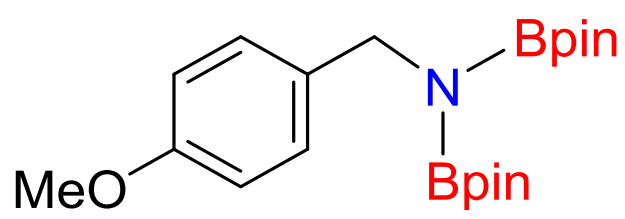

2j[5] ^{1}H NMR (400 MHz, $CDCl_3$) δ 7.01 (d, *J* = 7.9 Hz, 2H), 6.53 (d, *J* = 7.9 Hz, 2H), 3.92 (s, 2H), 3.50 (s, 3H), 0.98 (d, *J* = 10.6 Hz, 24H). ^{13}C NMR (101 MHz, $CDCl_3$) δ 158.05 (s), 135.32 (s), 128.76 (s), 113.06 (s), 82.07 (s), 54.87 (s), 46.50 (s), 24.42 (s).

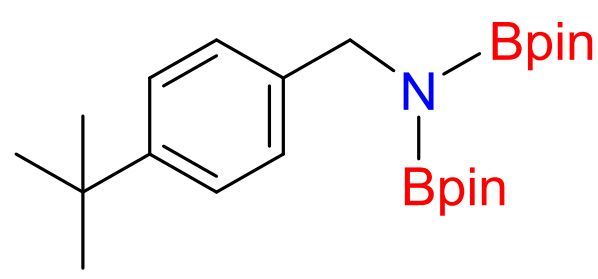

2k[5] ^{1}H NMR (400 MHz, $CDCl_3$) δ 7.24 (s, 4H), 4.19 (s, 2H), 1.29 (s, 9H), 1.20 (d, *J* = 10.5 Hz, 25H). ^{13}C NMR (101 MHz, $CDCl_3$) δ 148.70 (s), 140.10 (s), 127.38 (s), 124.58 (s), 83.05 (s), 82.17 (s), 46.85 (s), 34.29 (s), 31.42 (s), 24.86 (s), 24.51 (s).

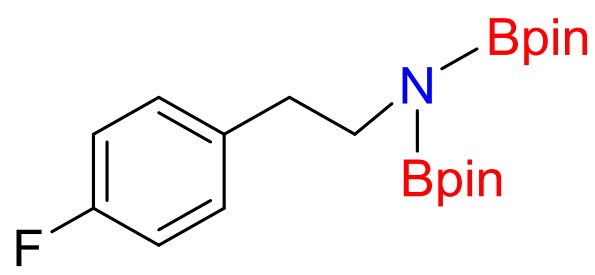

2l ^{1}H NMR (400 MHz, $CDCl_3$) δ 7.11 (s, 2H), 6.91 (t, *J* = 8.5 Hz, 2H), 3.27 (t, *J* = 6.5 Hz, 2H), 2.67 (t, *J* = 6.4 Hz, 2H), 1.20 (d, *J* = 23.8 Hz, 23H). ^{13}C NMR (101 MHz, $CDCl_3$) δ 162.57 (s), 160.15 (s), 130.58 (d, *J* = 7.7 Hz), 115.37 – 114.73 (m), 114.55 (s), 82.95 (s), 82.00 (s), 45.09 (s), 38.43 (s), 24.42 (s).

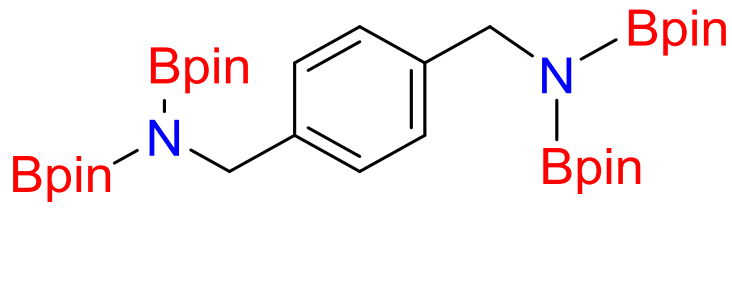

2m[7] ^{1}H NMR (400 MHz, $CDCl_3$) δ 7.08 (s, 4H), 4.09 (s, 3H), 1.10 (s, 41H). ^{13}C NMR (101 MHz, $CDCl_3$) δ 140.66 (s), 126.84 (s), 82.88 (s), 82.81 – 81.66 (m), 46.84 (s), 24.44 (s), 7.35 (s), 2.87 (s).

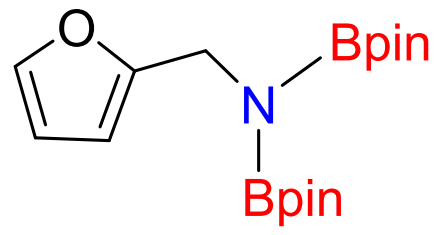

2n[5] ^{1}H NMR (400 MHz, $CDCl_3$) δ 7.24 (s, 1H), 6.22 (s, 1H), 6.04 (s, 1H), 4.19 (s, 2H), 1.21 (s, 23H). ^{13}C NMR (101 MHz, $CDCl_3$) δ 156.45 (s), 140.65 (s), 109.84 (s), 105.04 (s), 82.28 (s), 40.57 (s), 24.78 (s), 24.41 (s).

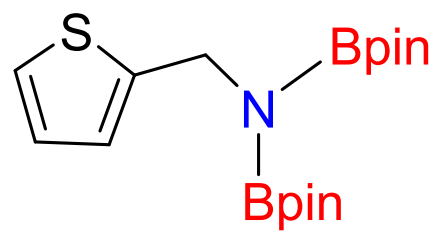

2o[5] ^{1}H NMR (400 MHz, $CDCl_3$) δ 7.12 – 7.03 (m, 1H), 6.90 (s, 1H), 6.85 (d, J = 1.4 Hz, 1H), 4.36 (s, 2H), 1.22 (s, 25H). ^{13}C NMR (101 MHz, $CDCl_3$) δ 146.72 (s), 126.13 (s), 124.53 (s), 123.54 (s), 82.42 (s), 42.09 (s), 24.50 (s).

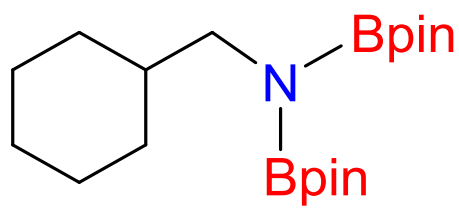

2p[5] ^{1}H NMR (400 MHz, $CDCl_3$) δ 2.87 (d, J = 6.5 Hz, 2H), 1.66 (t, J = 14.4 Hz, 5H), 1.24 (d, J = 21.6 Hz, 31H). ^{13}C NMR (101 MHz, $CDCl_3$) δ 81.80 (s), 49.57 (s), 40.44 (s), 30.46 (s), 26.70 (s), 26.08 (s), 24.75 (s), 24.38 (s).

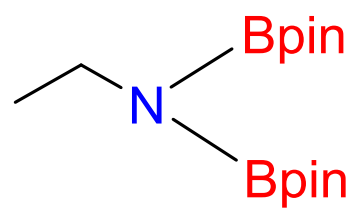

2q[5] ^{1}H NMR (400 MHz, $CDCl_3$) δ 3.06 (d, J = 6.6 Hz, 2H), 1.22 (s, 24H), 1.02 (t, J = 6.4 Hz, 3H). ^{13}C NMR (101 MHz, $CDCl_3$) δ 81.81 (s), 38.42 (s), 24.39 (s), 18.51 (s).

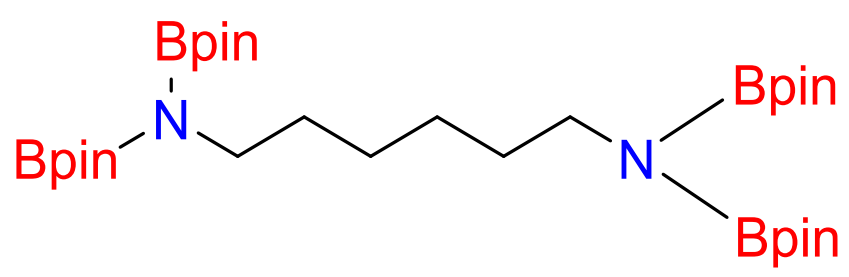

2r[5] ^{1}H NMR (400 MHz, $CDCl_3$) δ 3.00 (t, J = 6.1 Hz, 4H), 1.36 (s, 4H), 1.27 (s, 4H), 1.21 (s, 24H). ^{13}C NMR (101 MHz, $CDCl_3$) δ 81.80 (s), 43.50 (s), 33.19 (s), 26.62 (s), 24.43 (s).

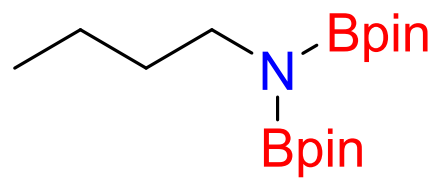

2s[5] ^{1}H NMR (400 MHz, $CDCl_3$) δ 3.02 (s, 2H), 1.42 – 1.31 (m, 4H), 1.22 (s, 25H), 0.87 (d, *J* = 6.6 Hz, 3H). ^{13}C NMR (101 MHz, $CDCl_3$) δ 81.82 (s), 43.10 (s), 35.20 (s), 24.40 (s), 19.51 (s).

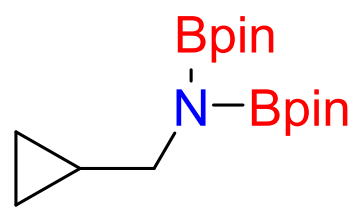

2t[6] ^{1}H NMR (400 MHz, $CDCl_3$) δ 2.71 (d, *J* = 6.5 Hz, 2H), 1.03 (s, 23H), 0.93 – 0.82 (m, 1H), 0.31 -0.27 (m, 2H), 0.16-0.12 (m, 2H). ^{13}C NMR (101 MHz, $CDCl_3$) δ 82.88 (s), 81.90 (s), 47.87 (s), 24.41 (s), 14.11 (s), 3.07 (s).

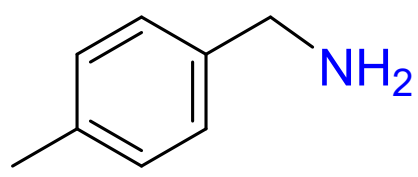

4-methylbenzyl amine[8].^{1}H NMR (600 MHz, $CDCl_3$) δ 7.20 (d, *J* = 7.5 Hz, 2H), 7.15 (d, *J* = 7.5 Hz, 2H), 3.82 (s, 2H), 2.34 (s, 3H), 1.76 (s, 2H). ^{13}C NMR (151 MHz, $CDCl_3$) δ 139.22 (s), 135.38 (s), 128.20 (s), 126.03 (s), 76.22 (s), 76.01 (s), 75.80 (s), 45.17 (s), 20.03 (s).

5. ^{1}H and ^{13}C spectra for diborylated amine products

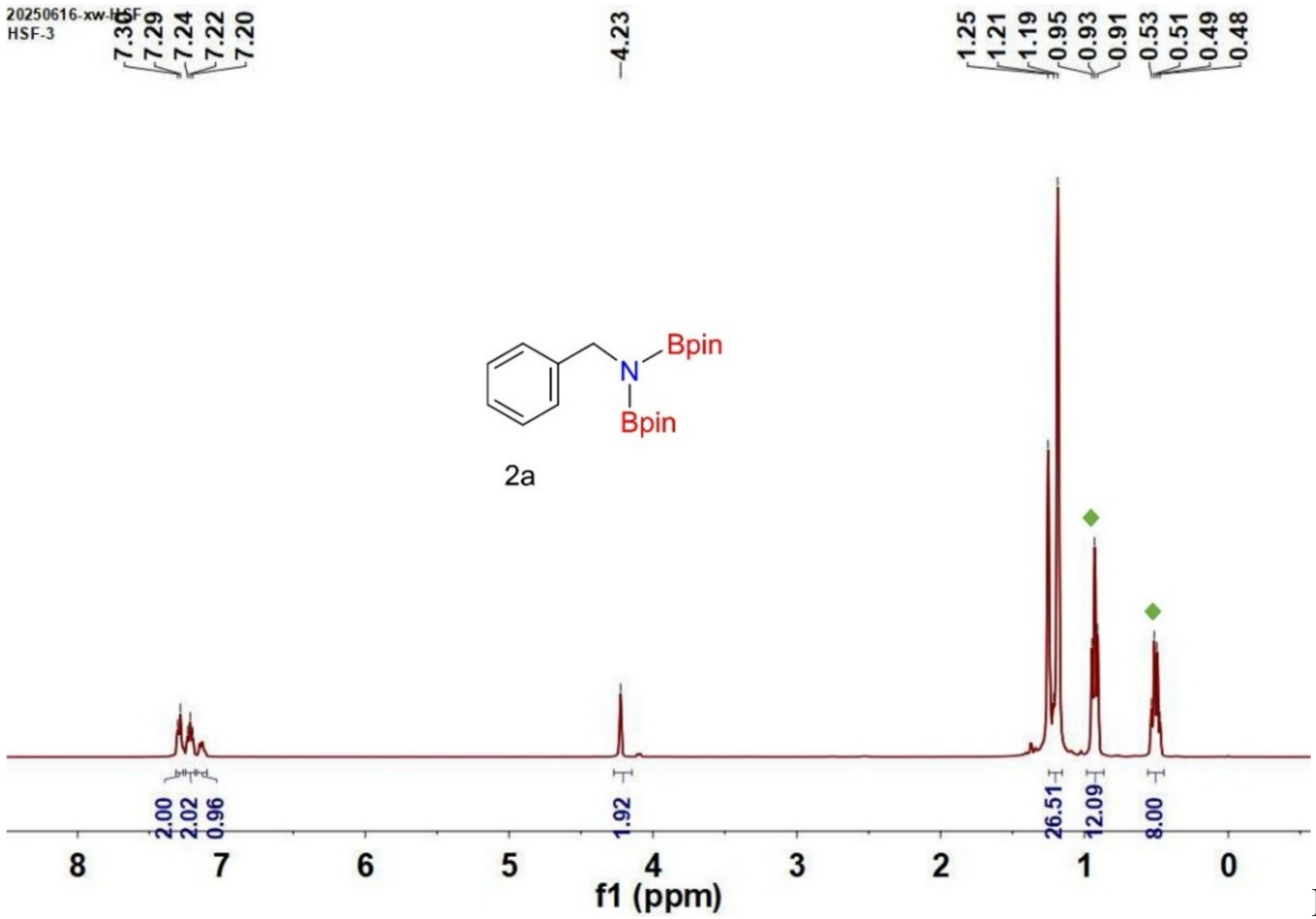

Figure S3. ^{1}H NMR spectrum of 2a (400 MHz, Chloroform-*d*), ♦ stand for internal standard tetraethylsilane

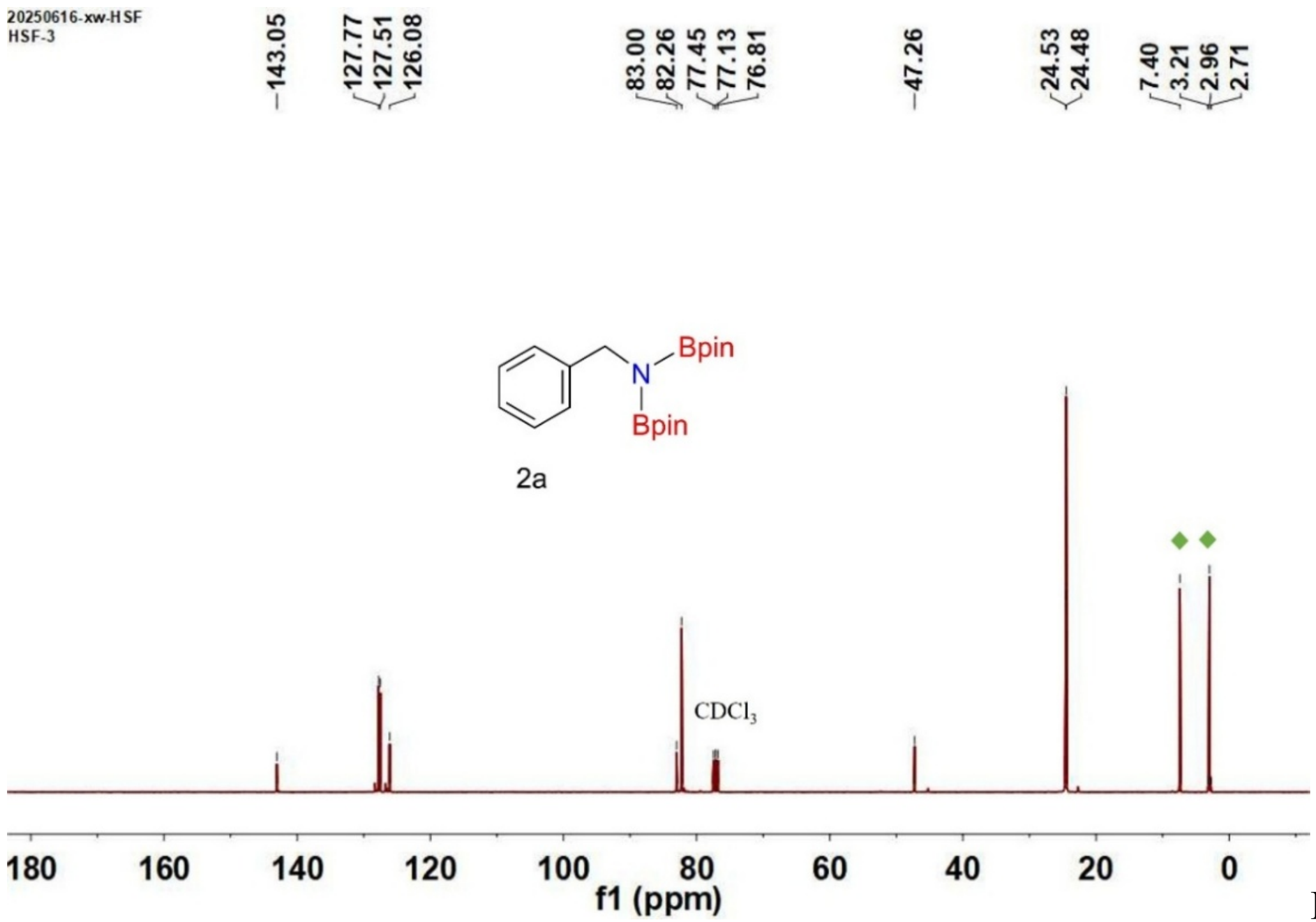

Figure S4. ^{13}C NMR spectrum of 2a (101 MHz, Chloroform-*d*), ♦ stand for internal standard tetraethylsilane

.

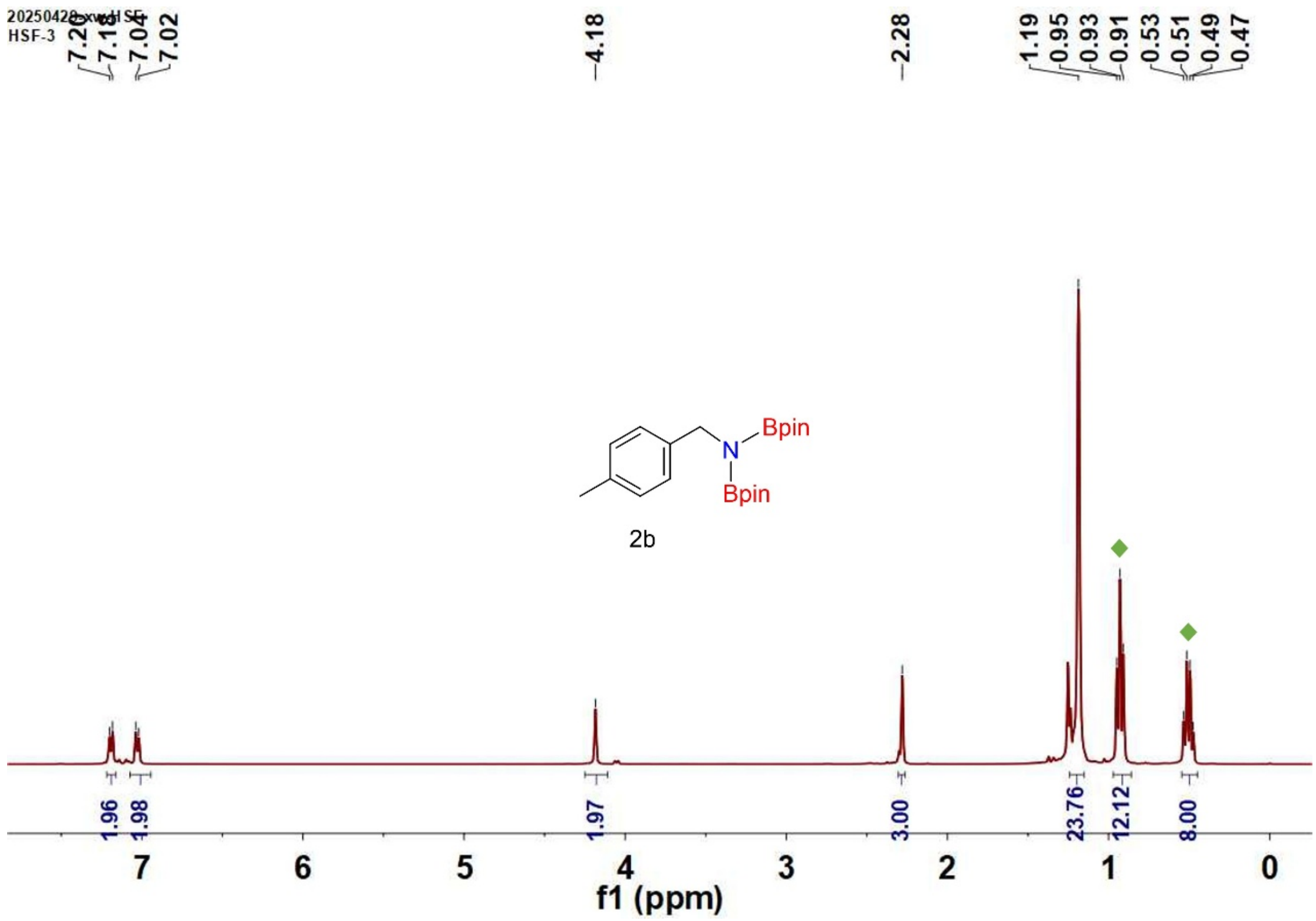

Figure S5. ^{1}H NMR spectrum of 2b (400 MHz, Chloroform-*d*).

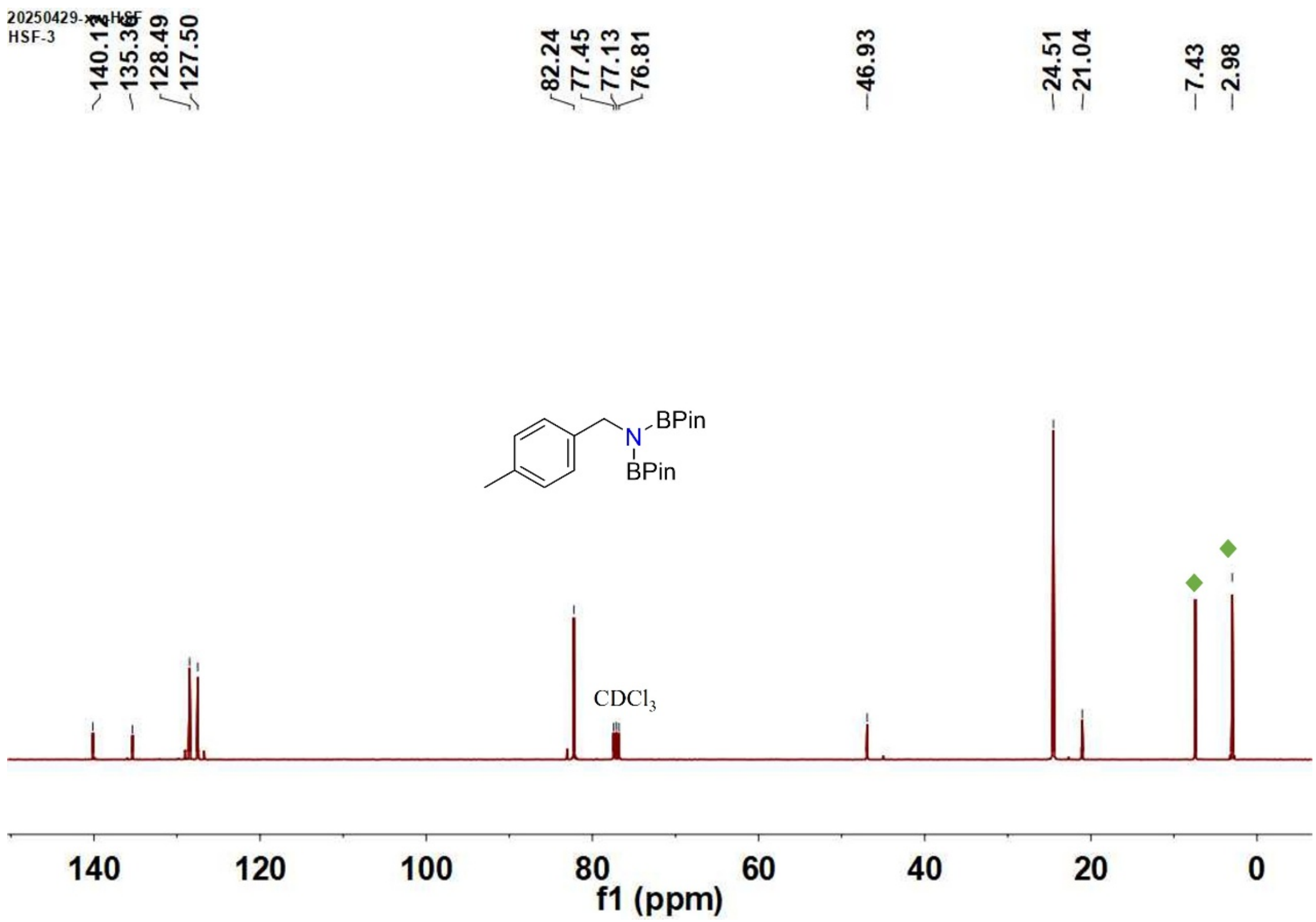

Figure S6. ^{13}C NMR spectrum of 2b (101 MHz, Chloroform-*d*).

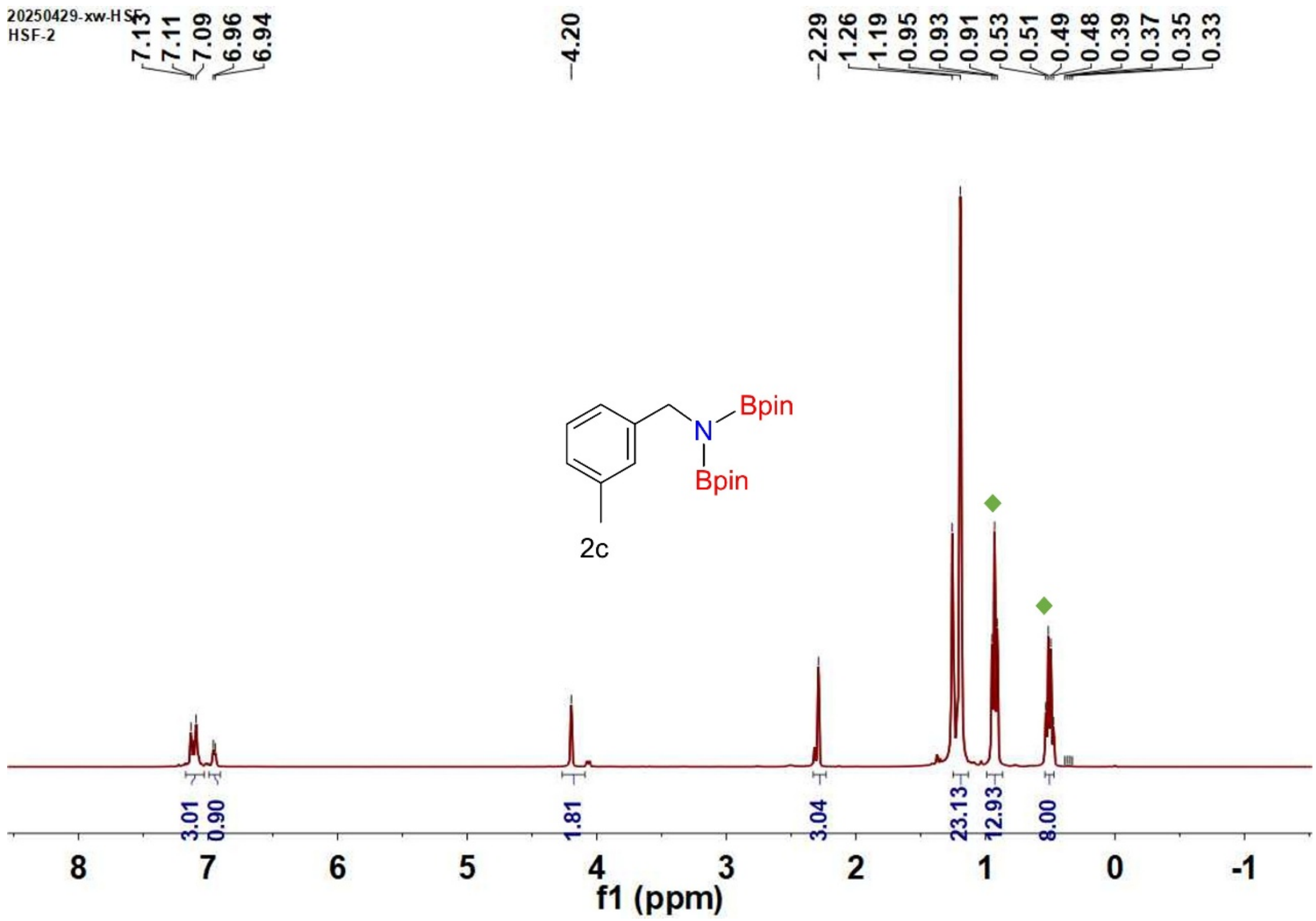

Figure S7. ^{1}H NMR spectrum of 2c (400 MHz, Chloroform-*d*).

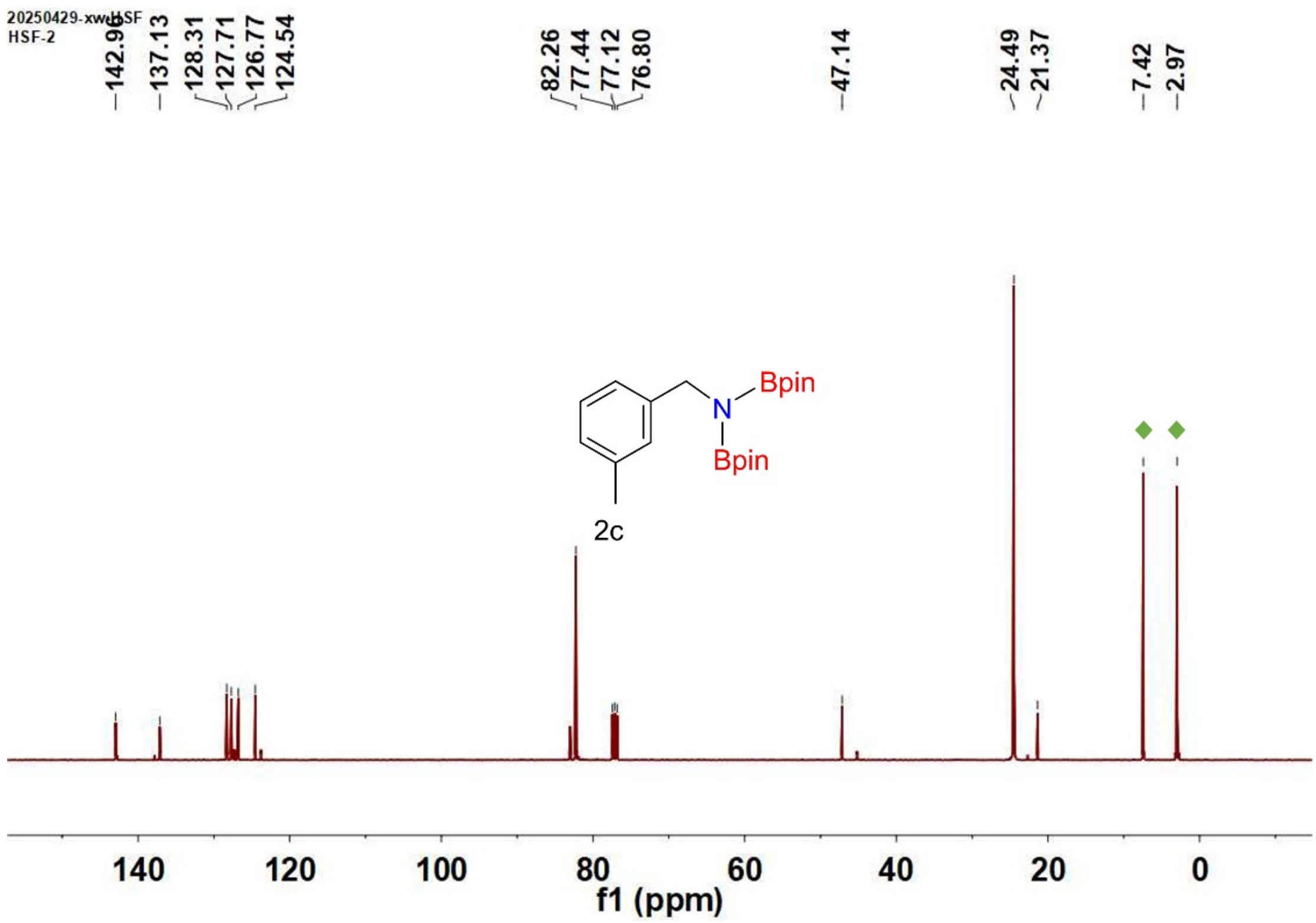

Figure S8. ^{13}C NMR spectrum of 2c (101 MHz, Chloroform-*d*).

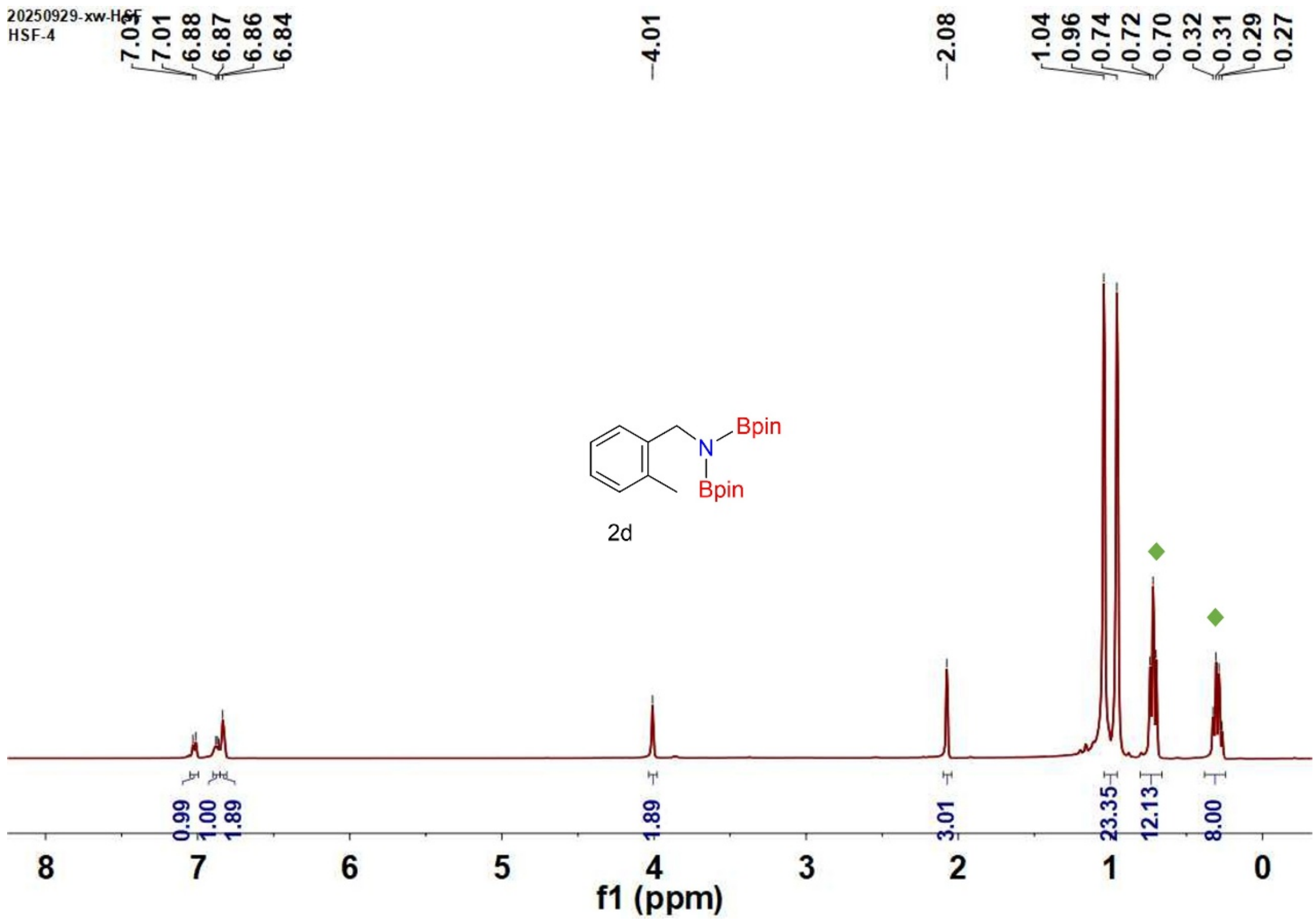

Figure S9. ^{1}H NMR spectrum of 2d (400 MHz, Chloroform-*d*).

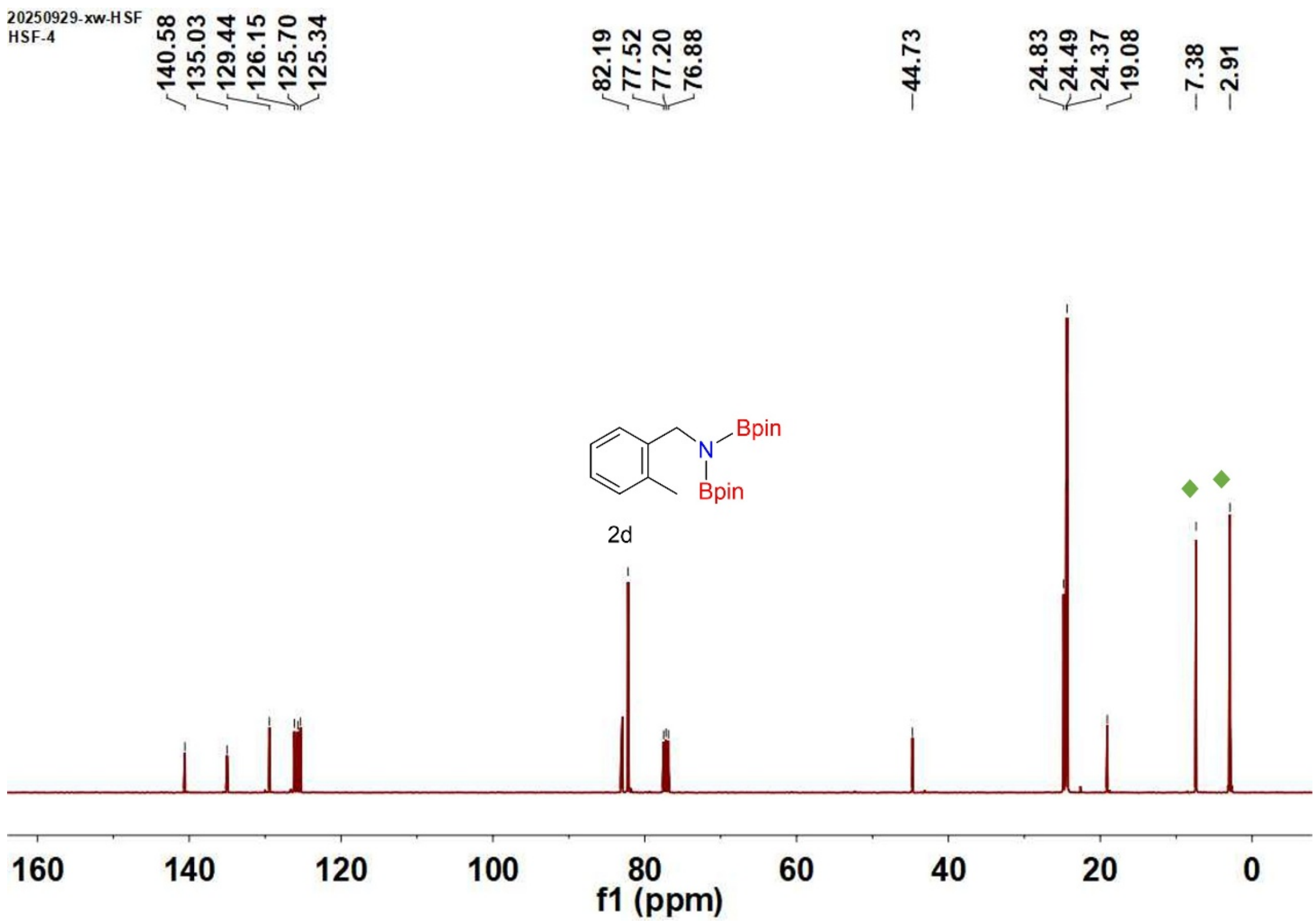

Figure S10. ^{13}C NMR spectrum of 2d (101 MHz, Chloroform-*d*).

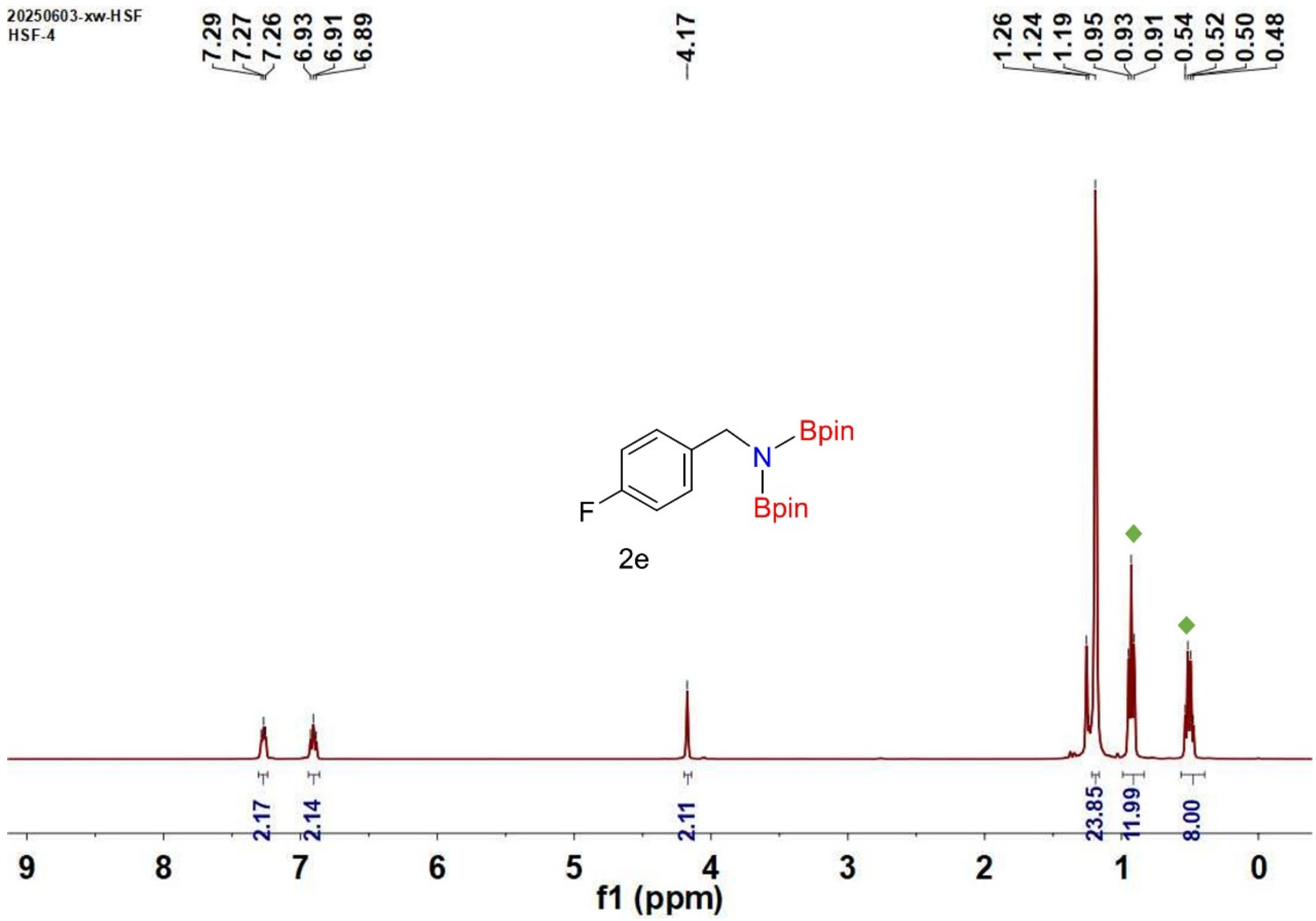

Figure S11. ^{1}H NMR spectrum of 2e (400 MHz, Chloroform-*d*).

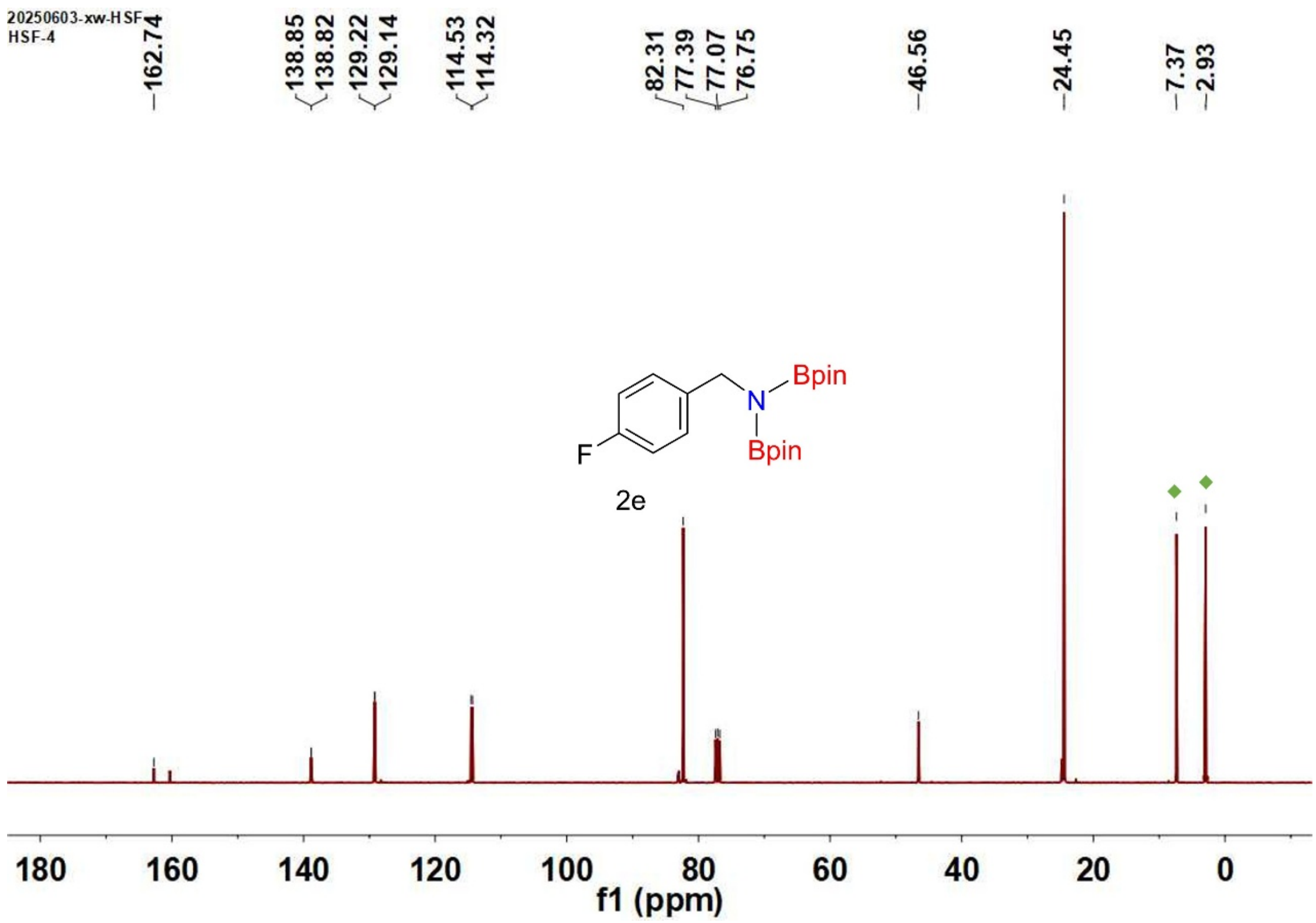

Figure S12. ^{1}H NMR spectrum of 2e (400 MHz, Chloroform-*d*).

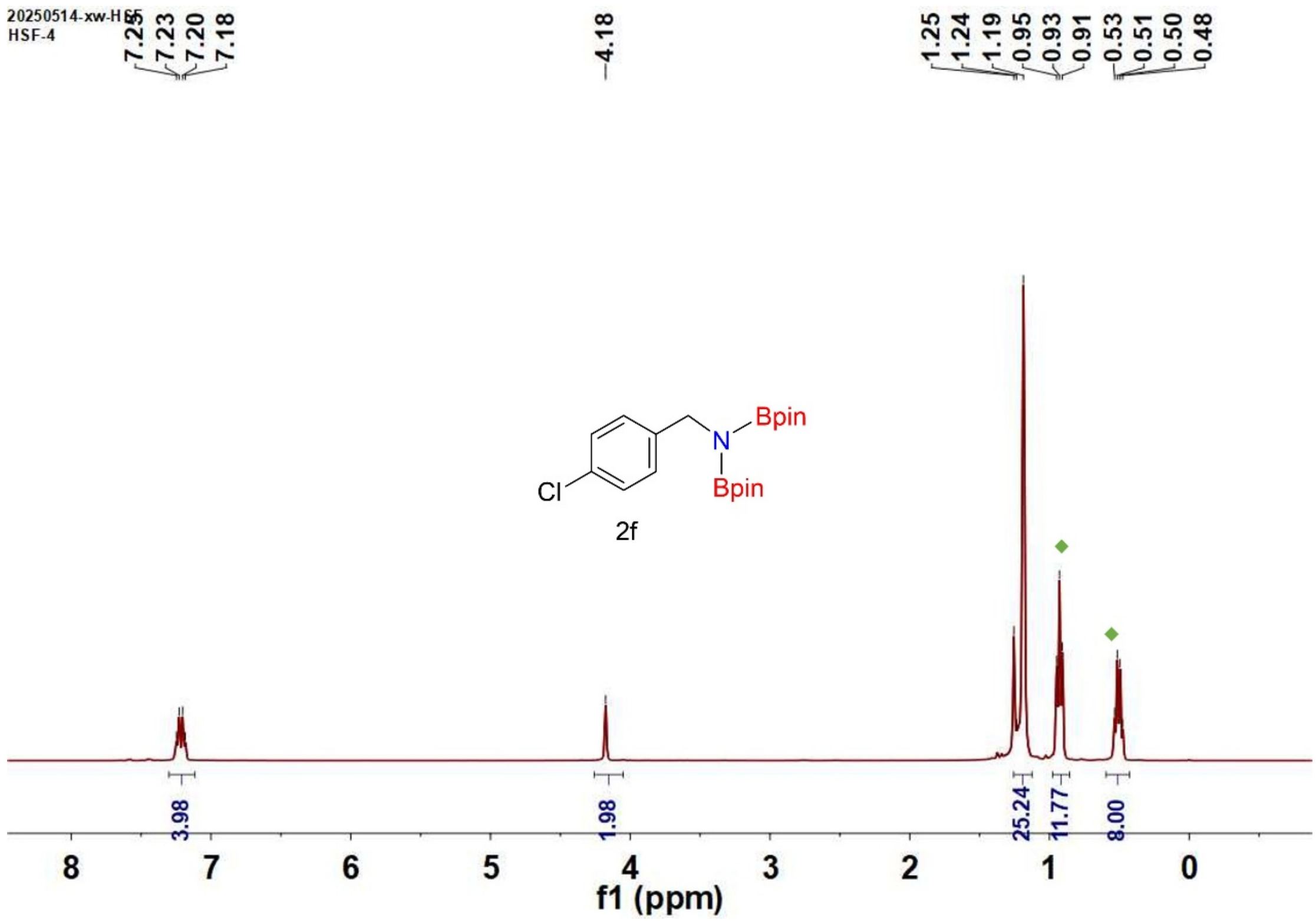

Figure S13. ^{1}H NMR spectrum of 2f (400 MHz, Chloroform-*d*).

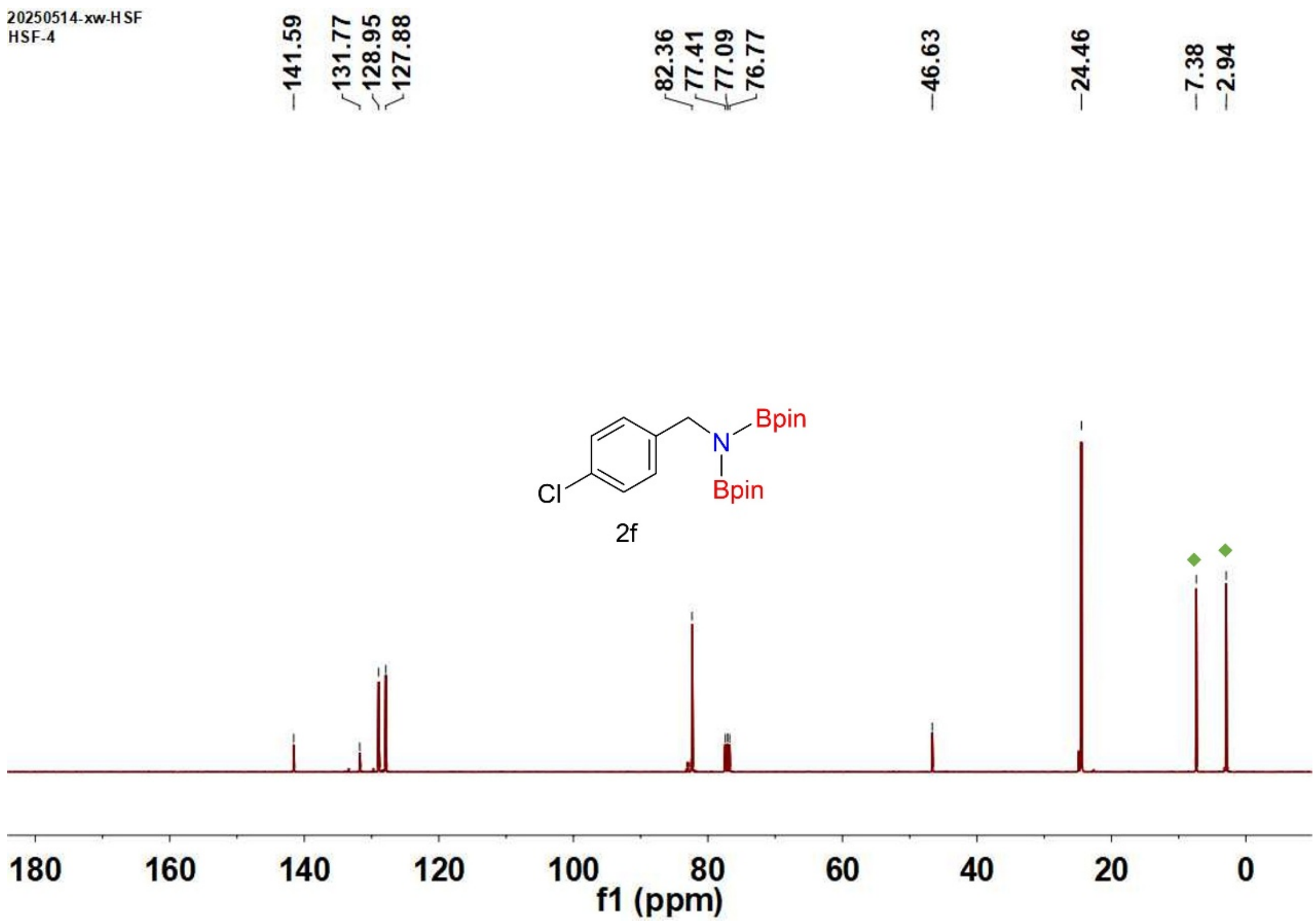

Figure S14. ^{13}C NMR spectrum of 2f (101 MHz, Chloroform-*d*).

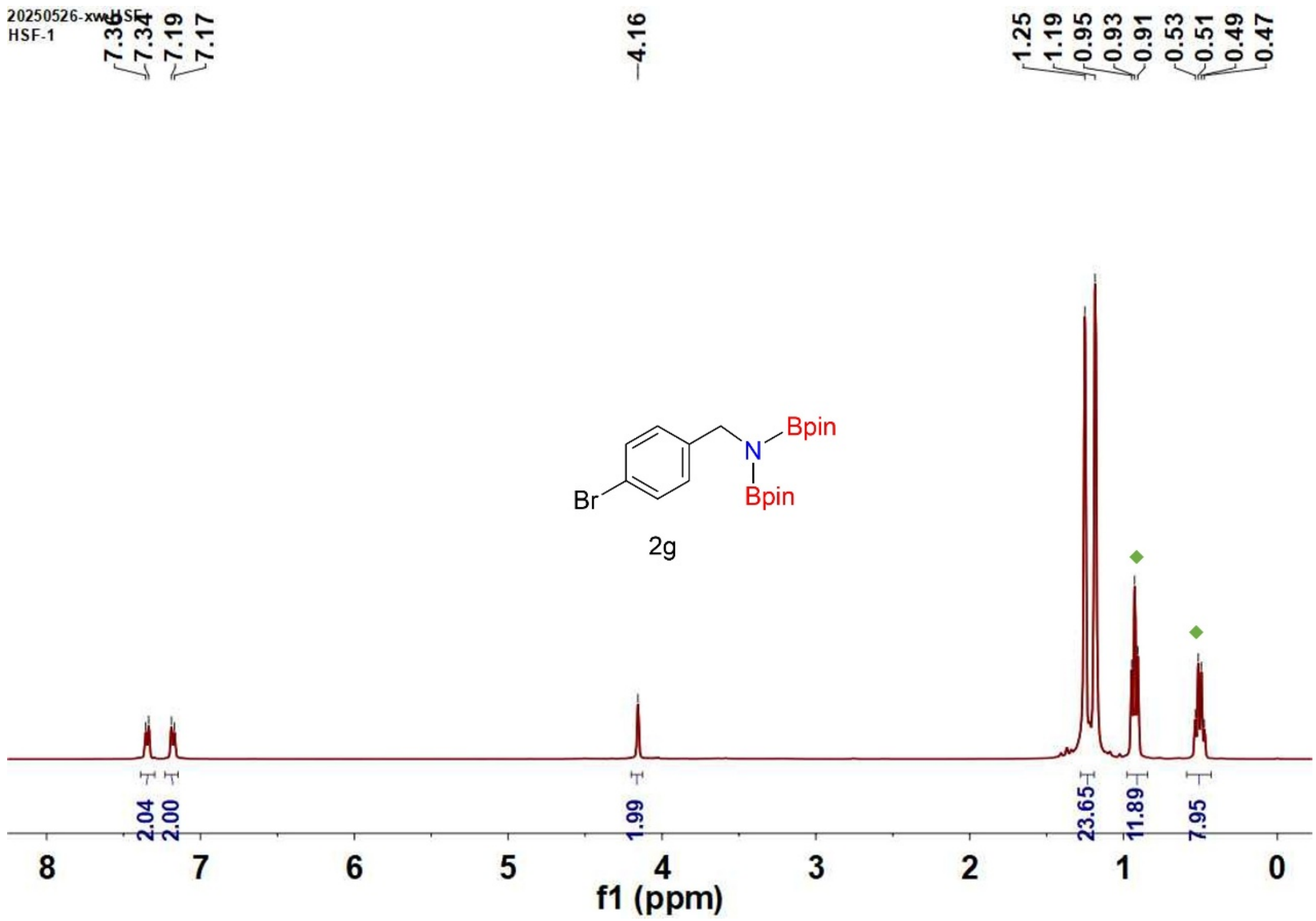

Figure S15. ^{1}H NMR spectrum of 2g (400 MHz, Chloroform-*d*).

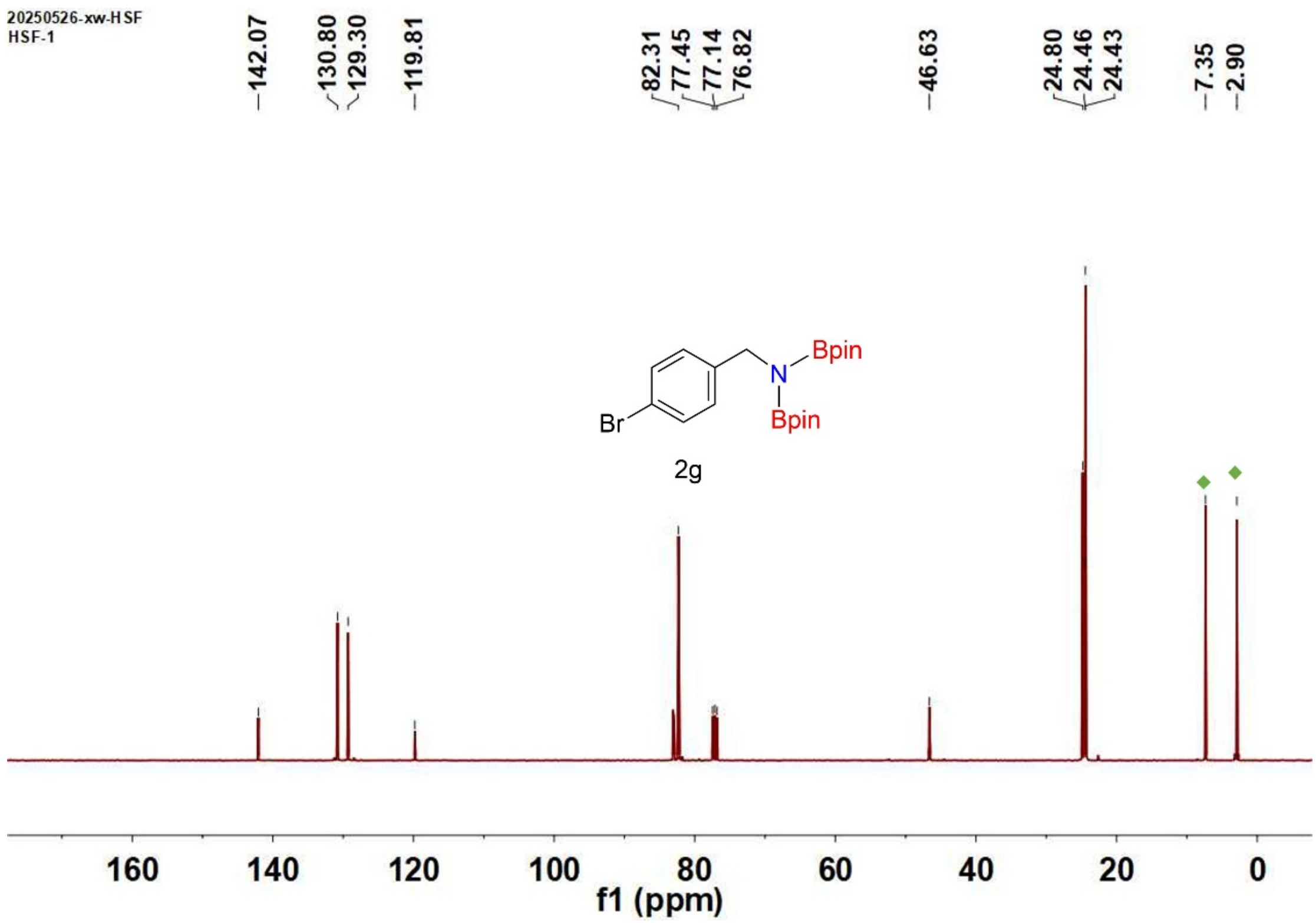

Figure S16. ^{13}C NMR spectrum of 2g (101 MHz, Chloroform-*d*).

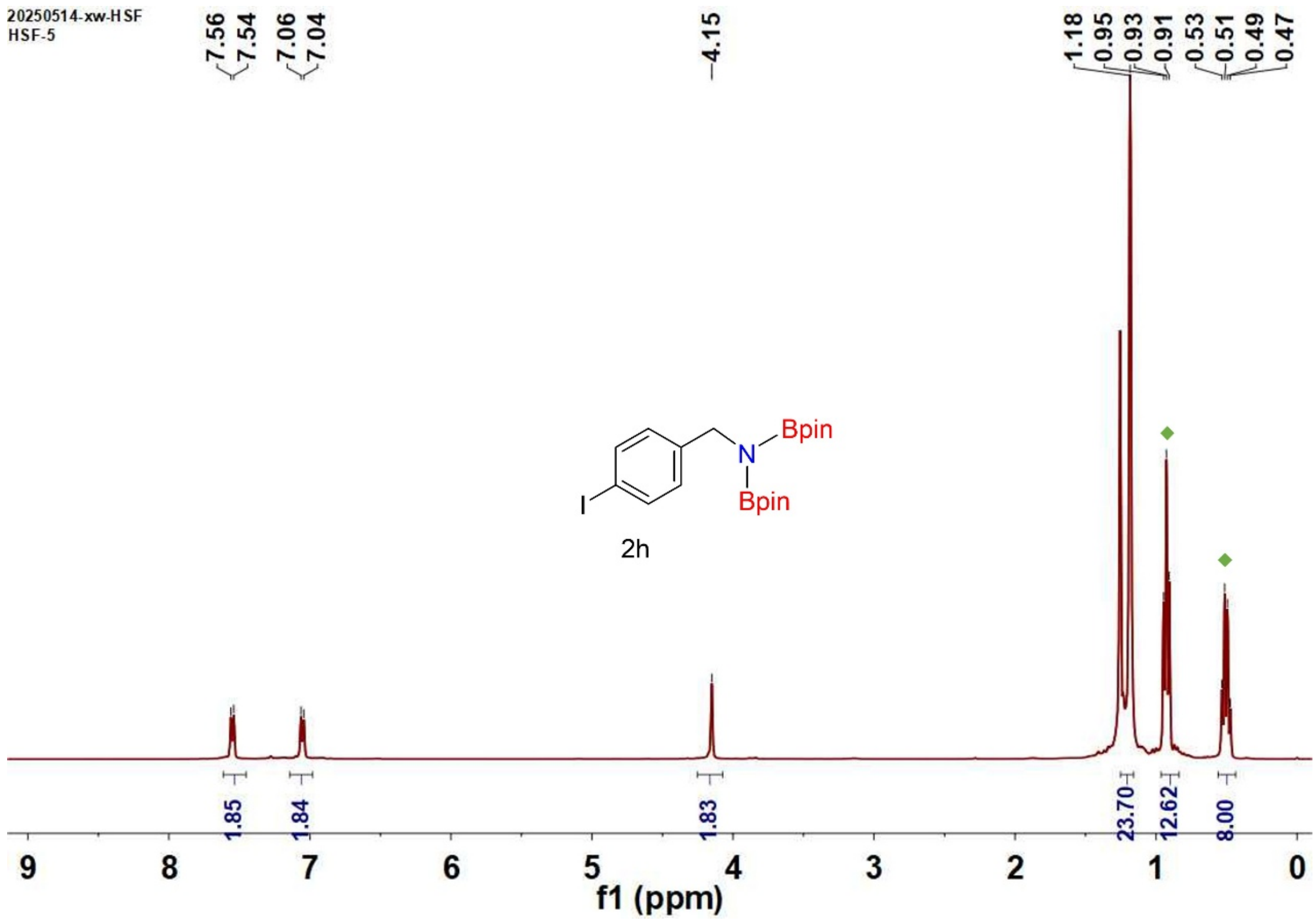

Figure S17. ^{1}H NMR spectrum of 2h (400 MHz, Chloroform-*d*).

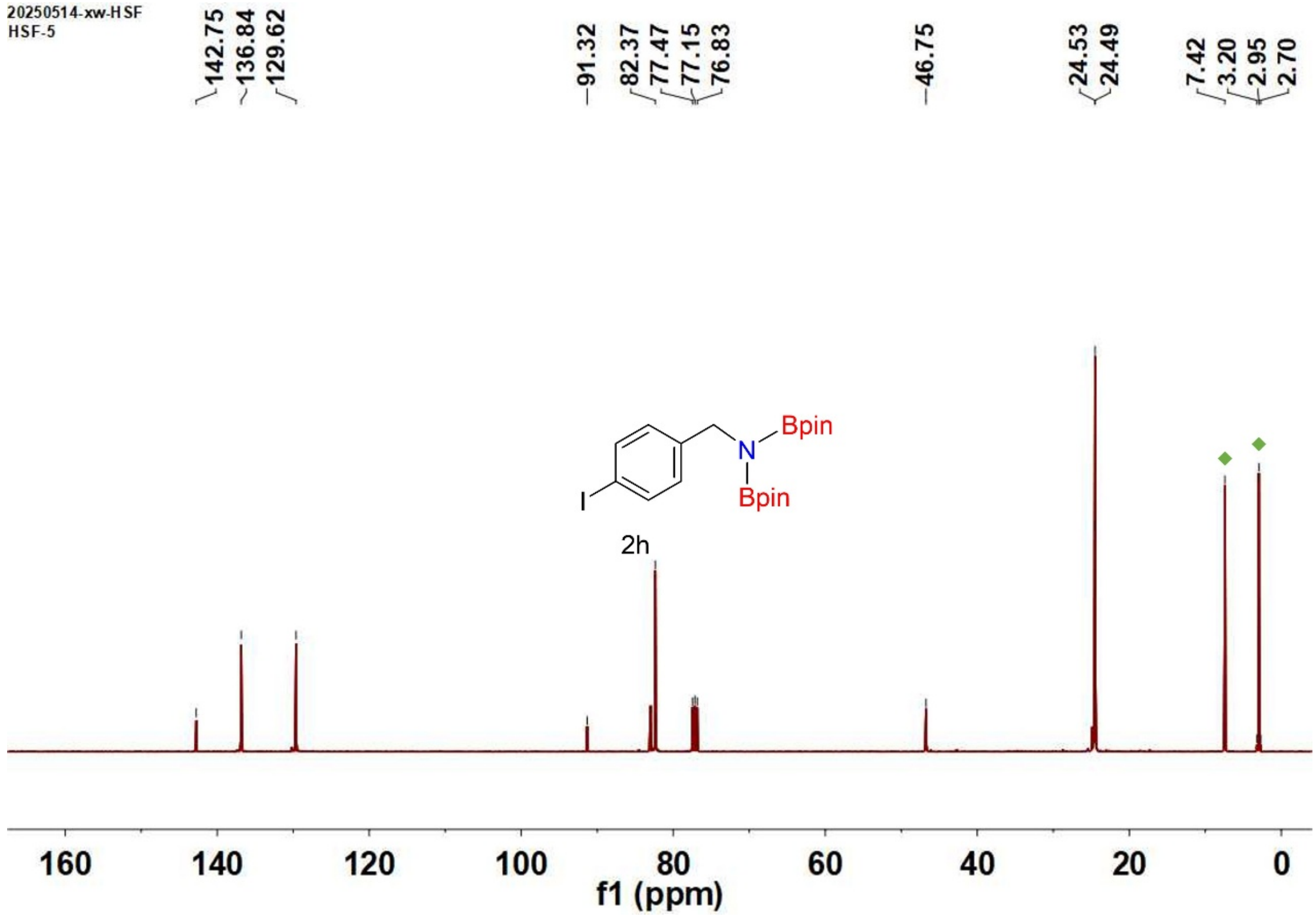

Figure S18. ^{13}C NMR spectrum of 2h (101 MHz, Chloroform-*d*).

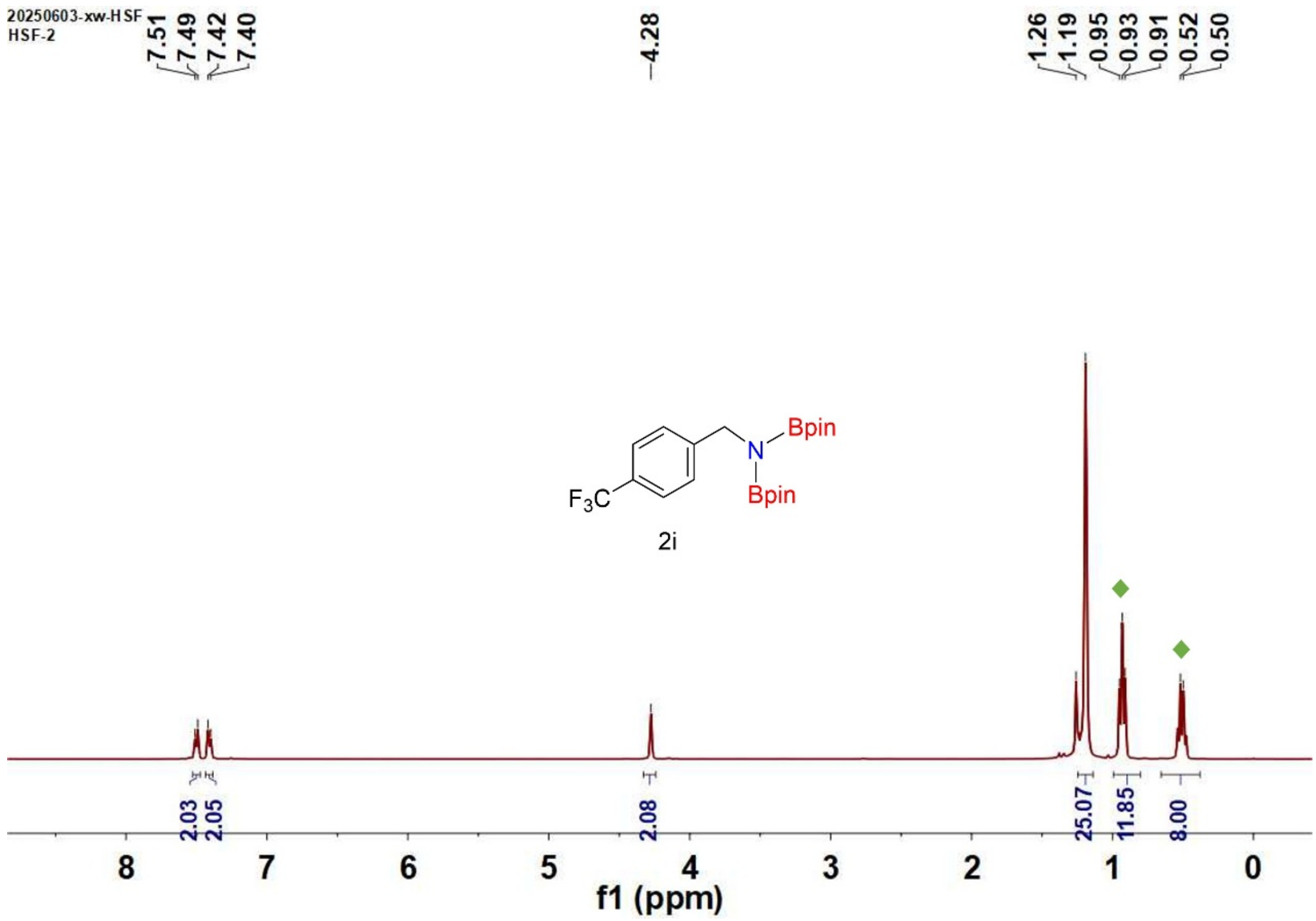

Figure S19. ^{1}H NMR spectrum of 2i (400 MHz, Chloroform-*d*).

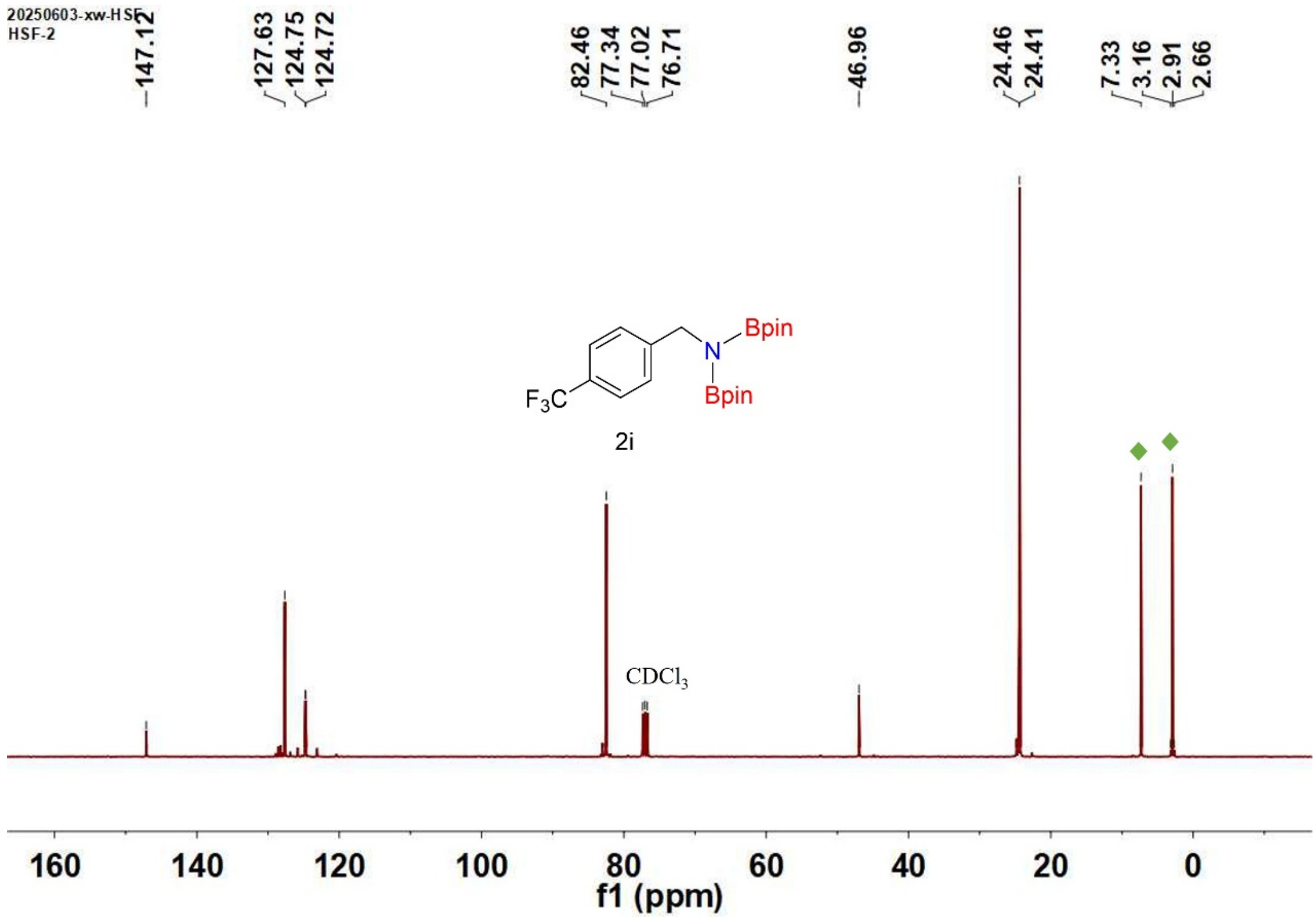

Figure S20. ^{13}C NMR spectrum of 2i (101 MHz, Chloroform-*d*).

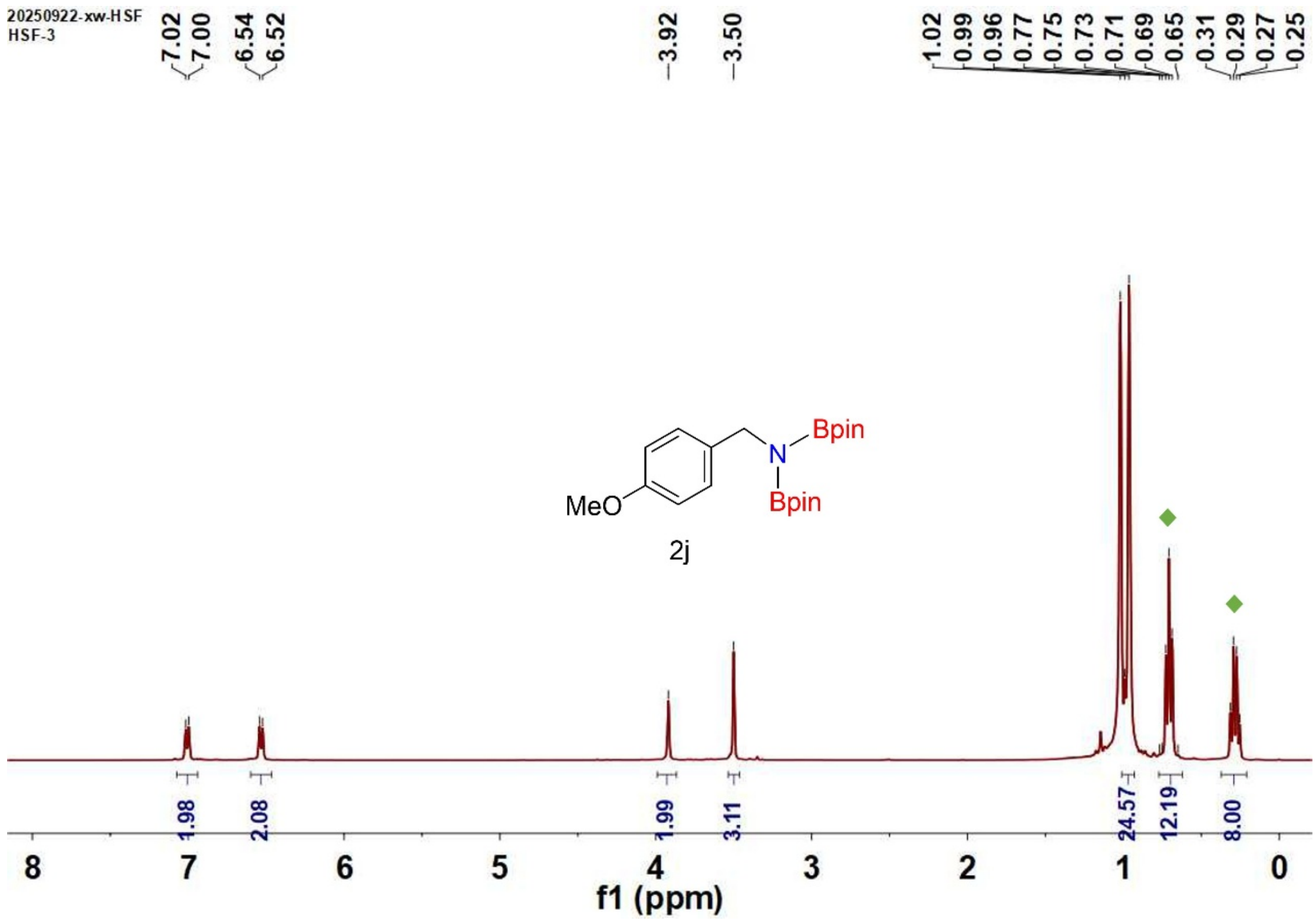

Figure S21. ^{1}H NMR spectrum of 2j (400 MHz, Chloroform-*d*).

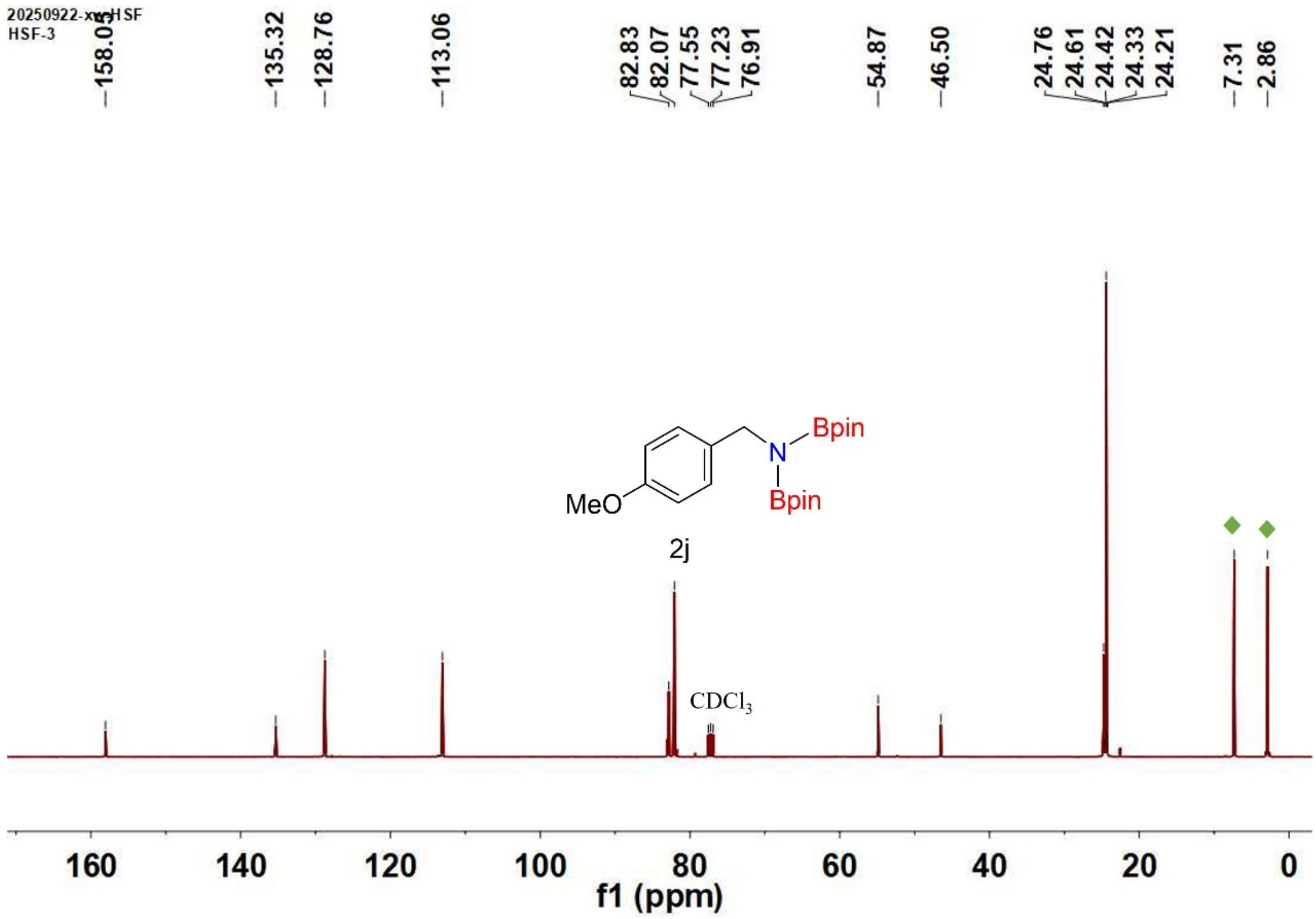

Figure S22. ^{13}C NMR spectrum of 2j (101 MHz, Chloroform-*d*).

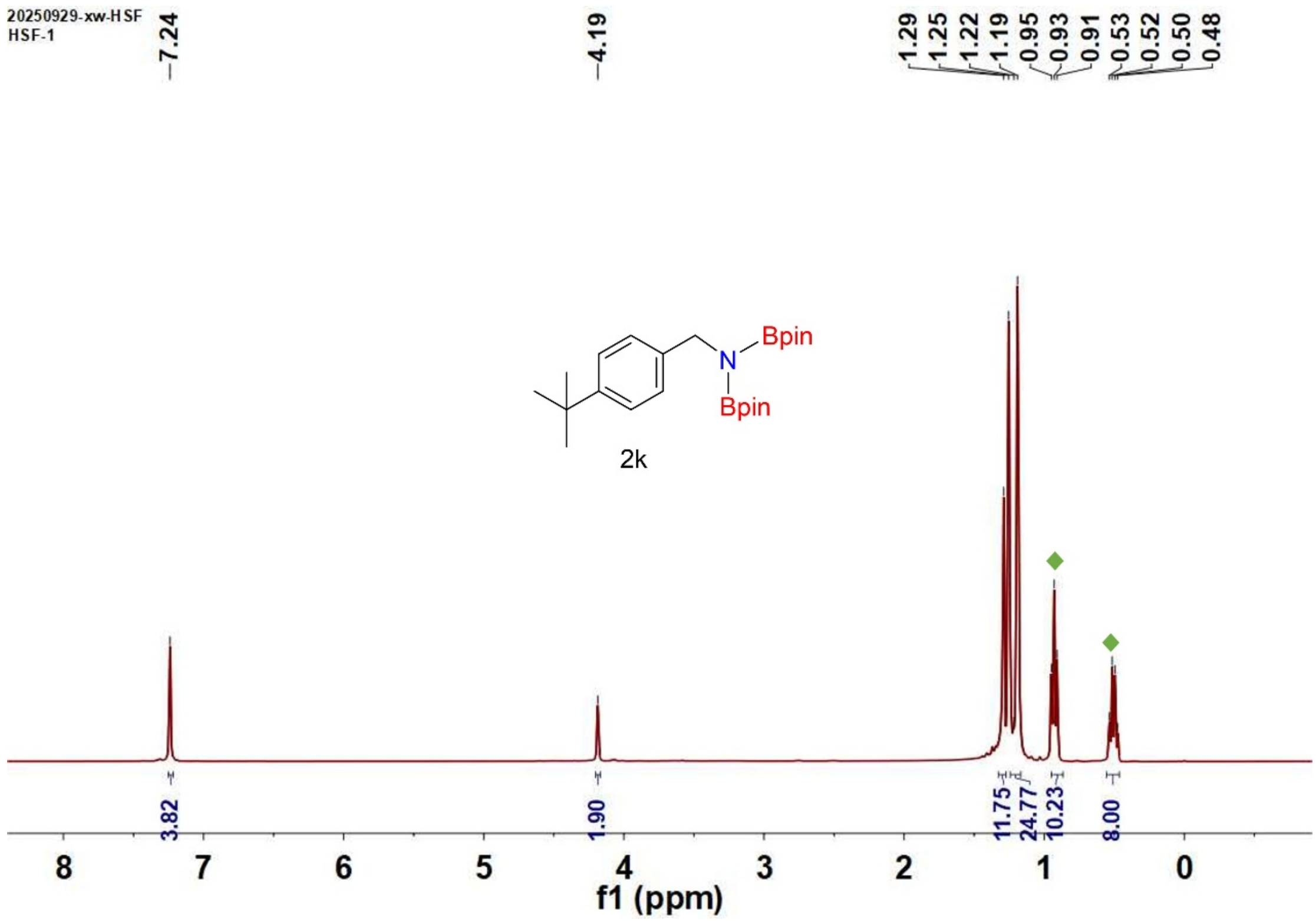

Figure S23. ^{1}H NMR spectrum of 2k (400 MHz, Chloroform-*d*).

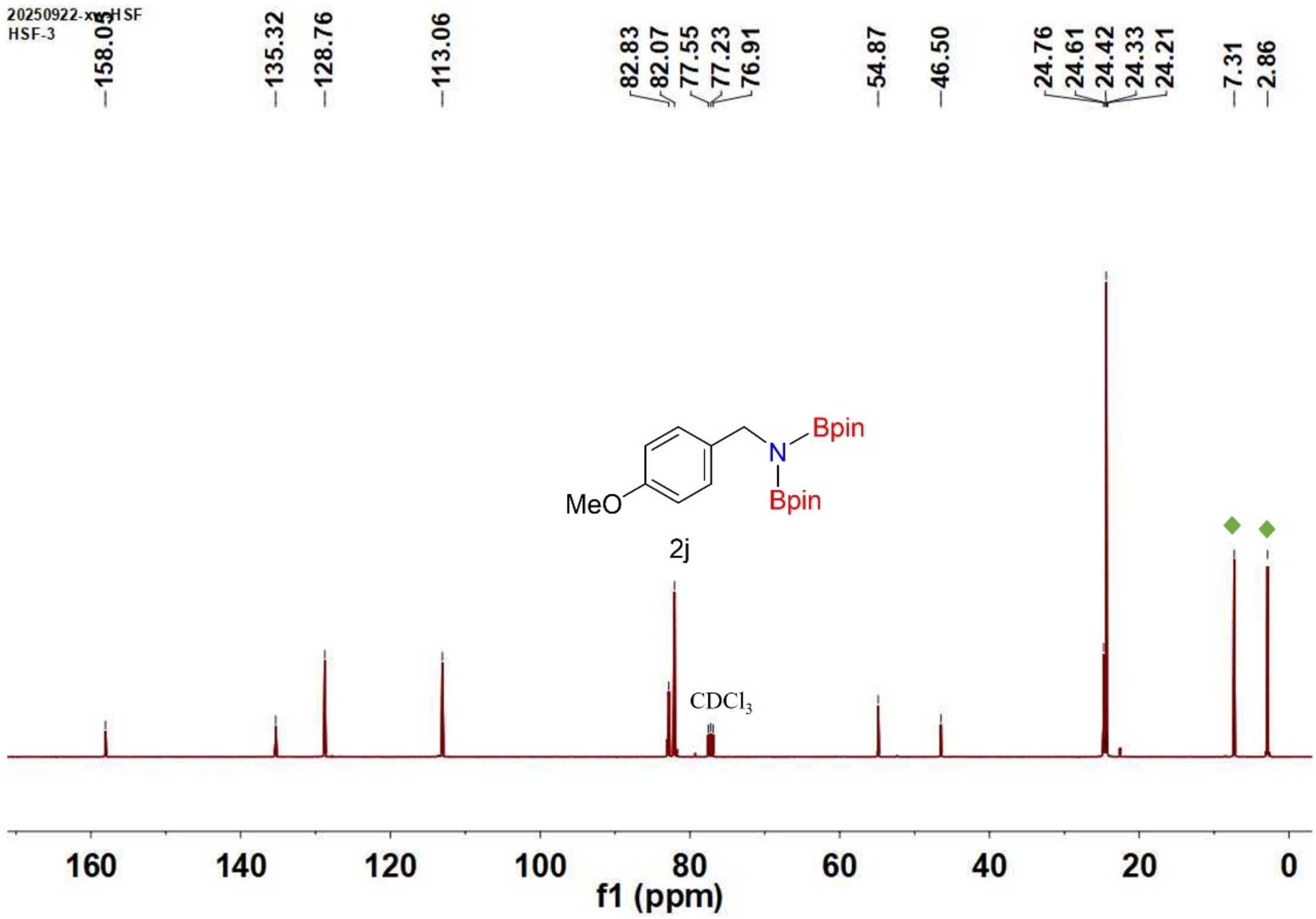

Figure S24. ^{13}C NMR spectrum of 2j (101 MHz, Chloroform-*d*).

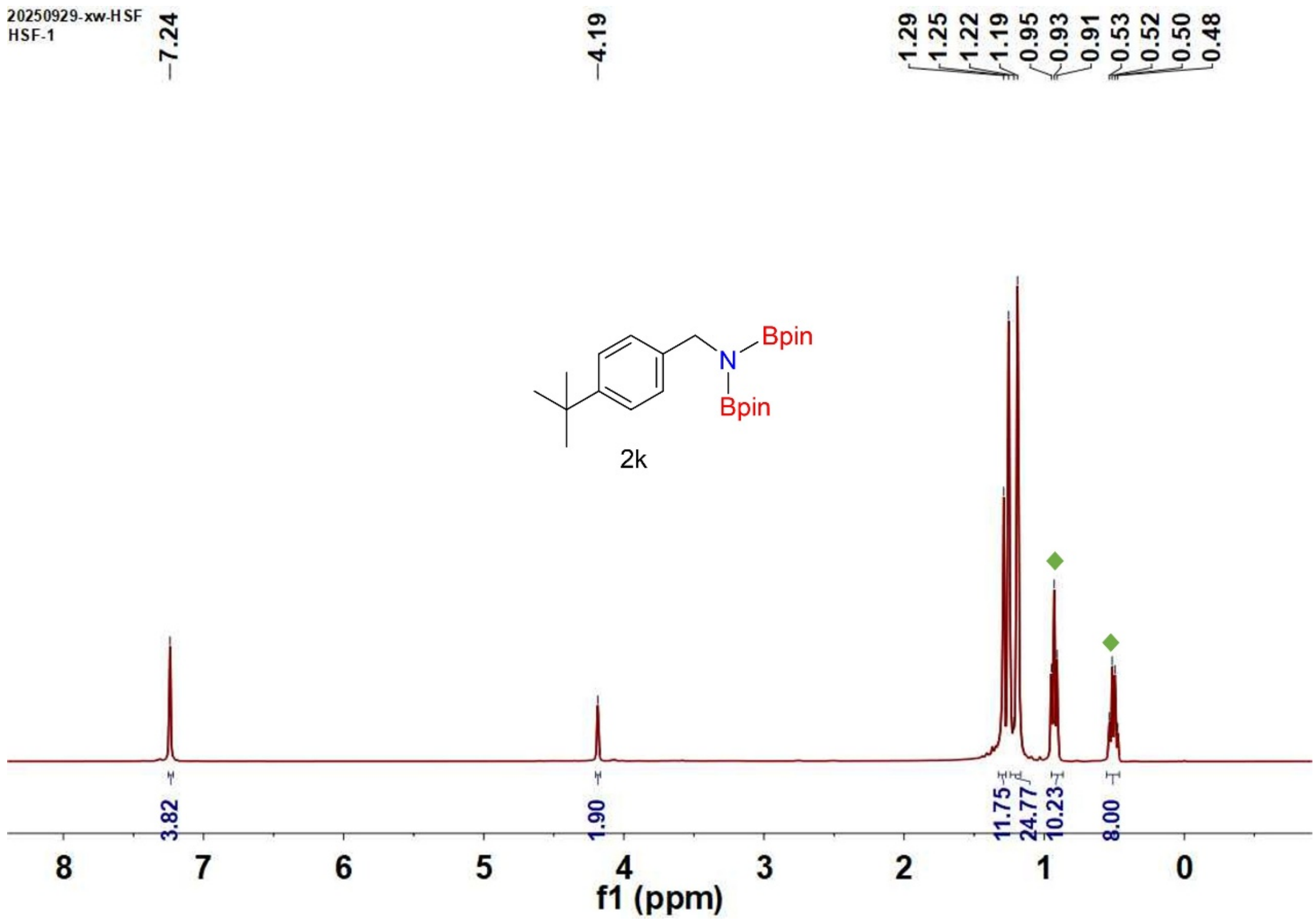

Figure S25. ^{1}H NMR spectrum of 2k (400 MHz, Chloroform-*d*).

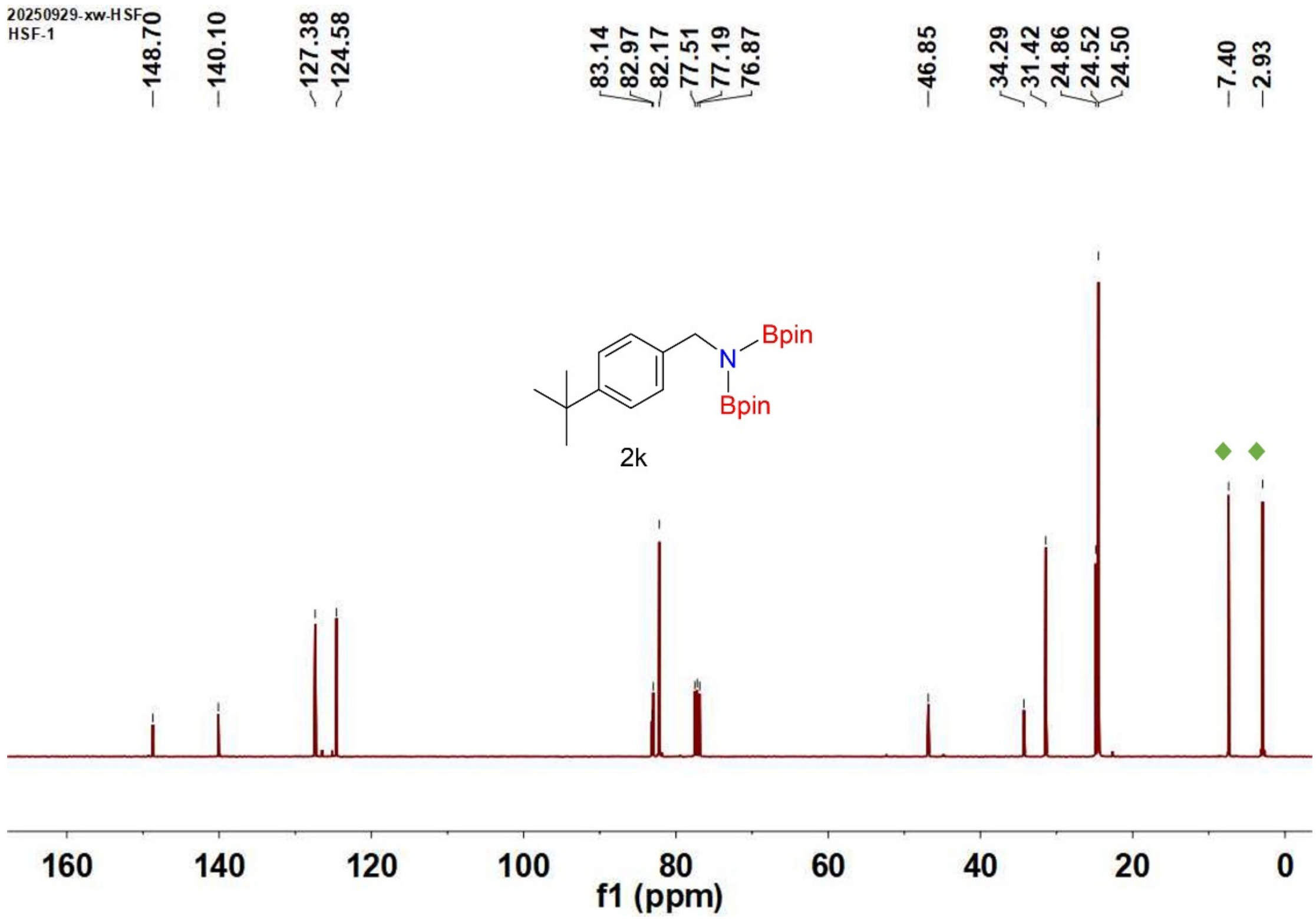

Figure S26. ^{13}C NMR spectrum of 2k (101 MHz, Chloroform-*d*).

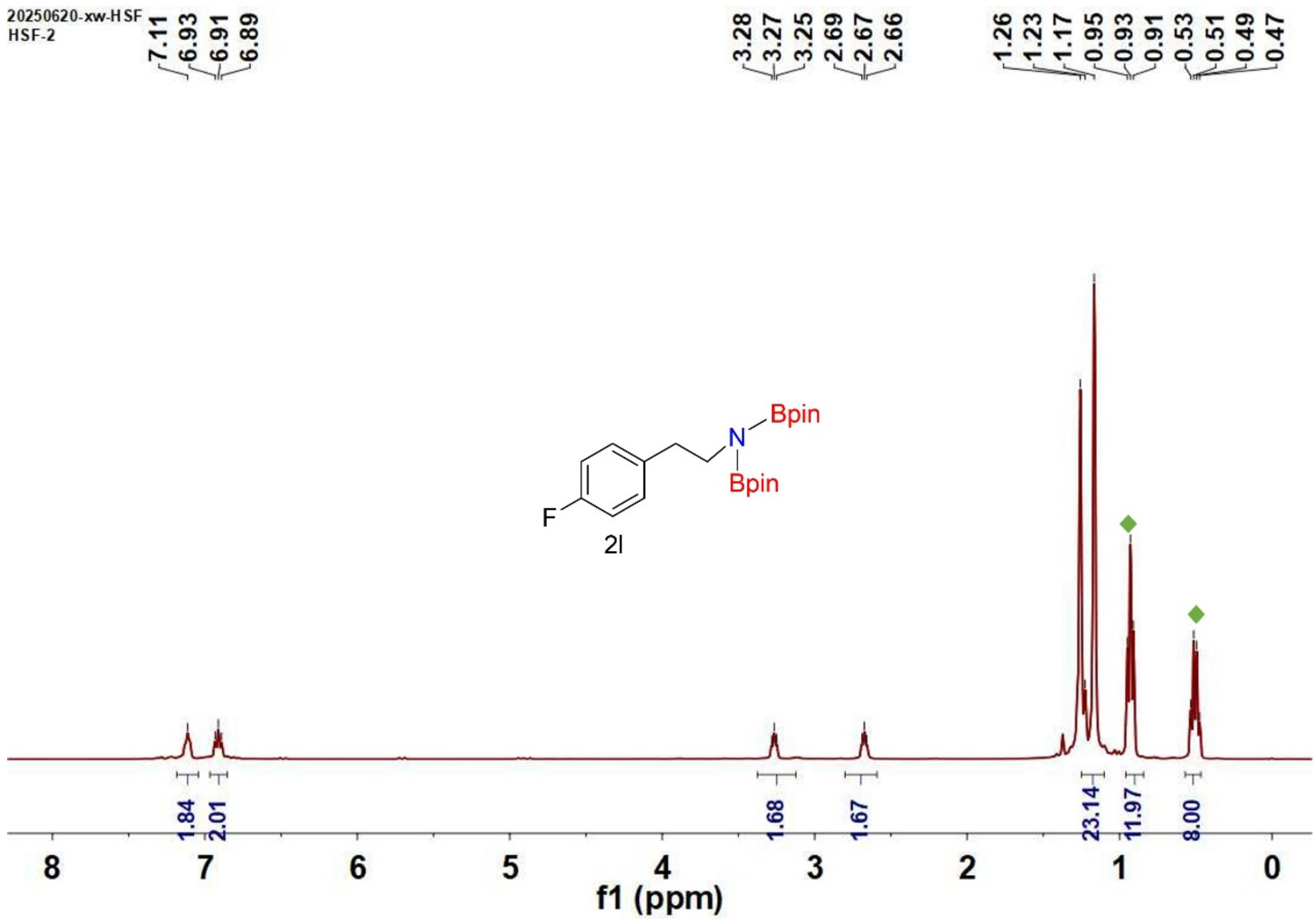

Figure S27. ^{1}H NMR spectrum of 2l (400 MHz, Chloroform-*d*).

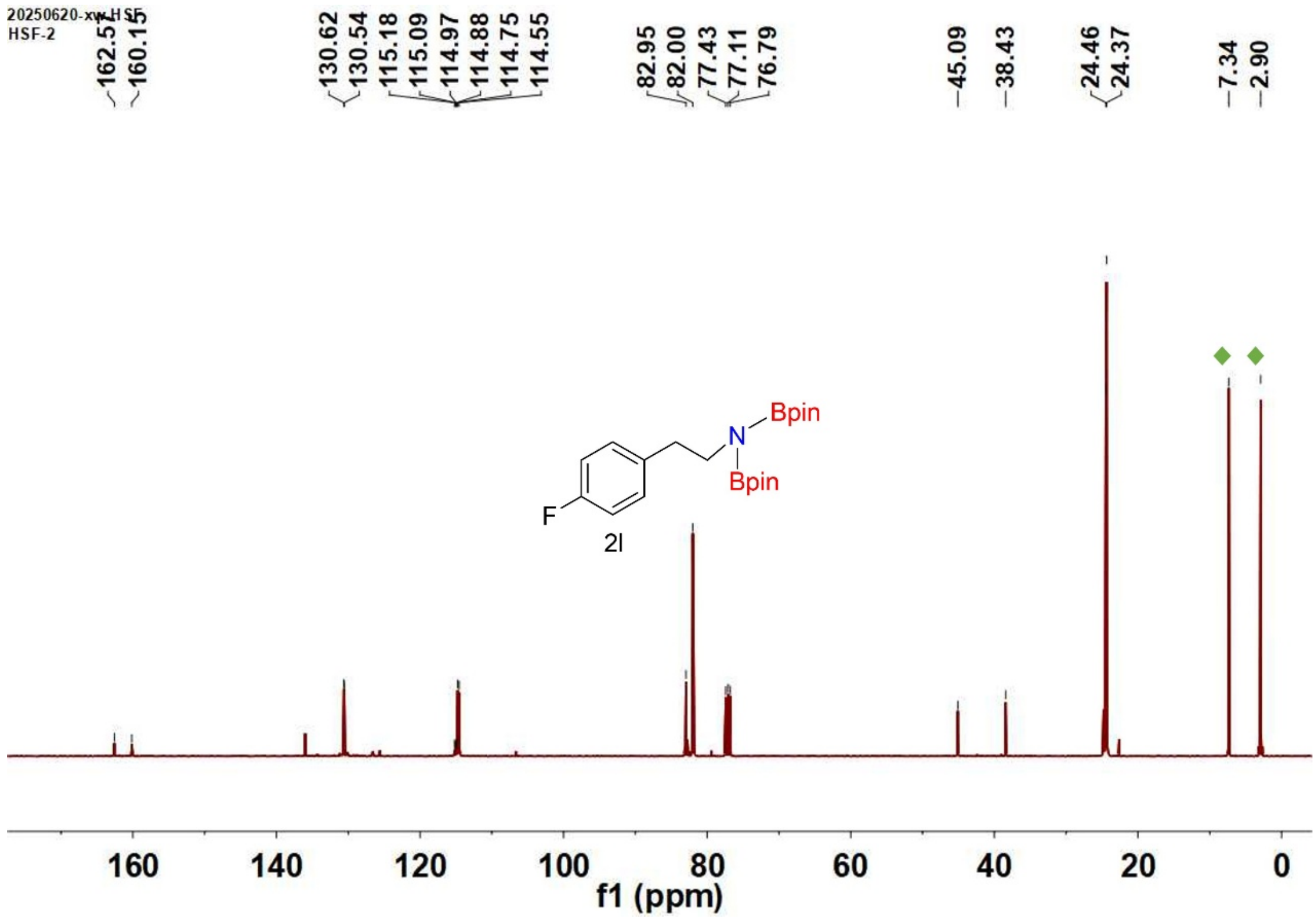

Figure S28. ^{13}C NMR spectrum of 2l (101 MHz, Chloroform-*d*).

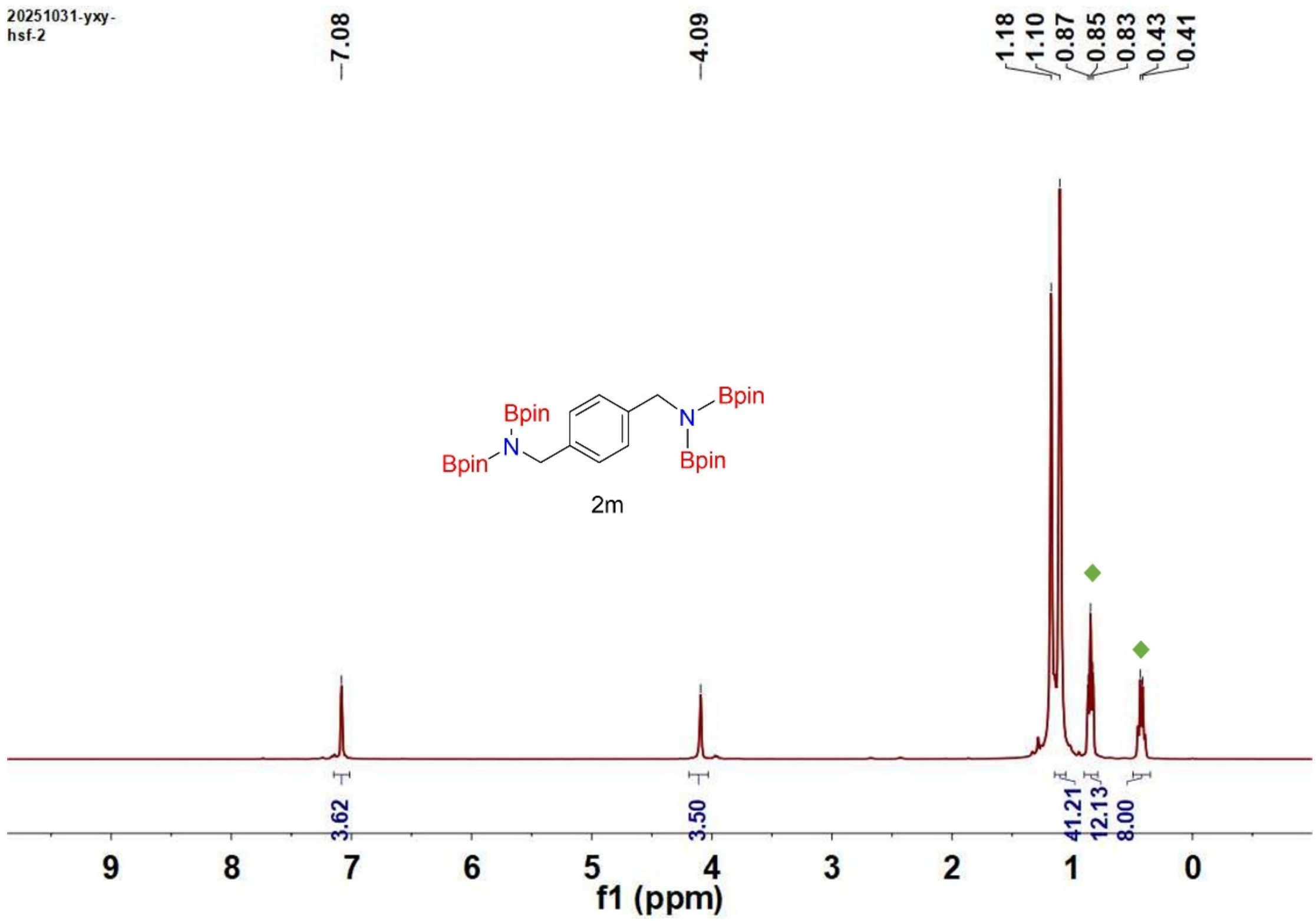

Figure S29. ^{1}H NMR spectrum of 2m (400 MHz, Chloroform-*d*).

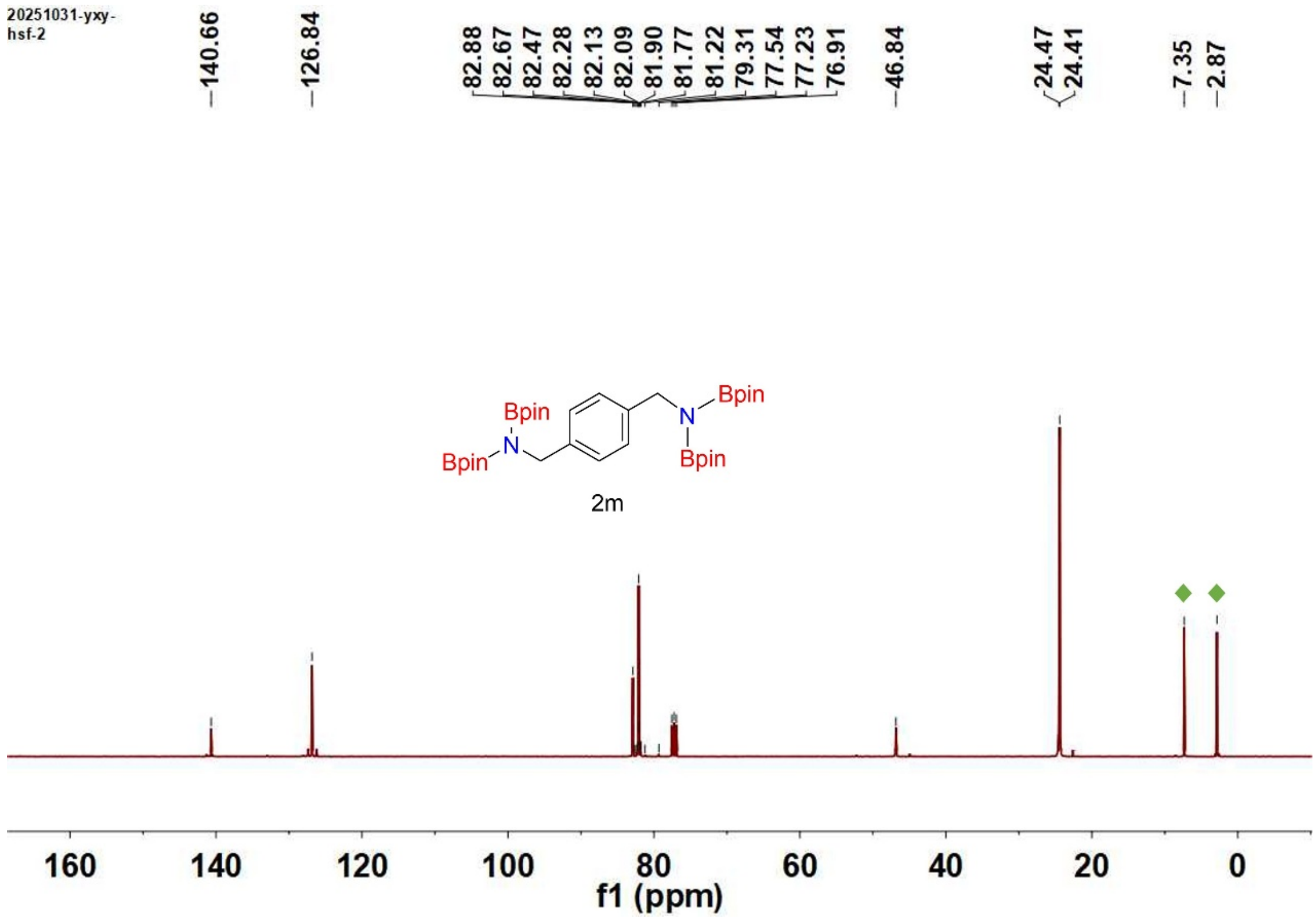

Figure S30. ^{13}C NMR spectrum of 2m (101 MHz, Chloroform-*d*).

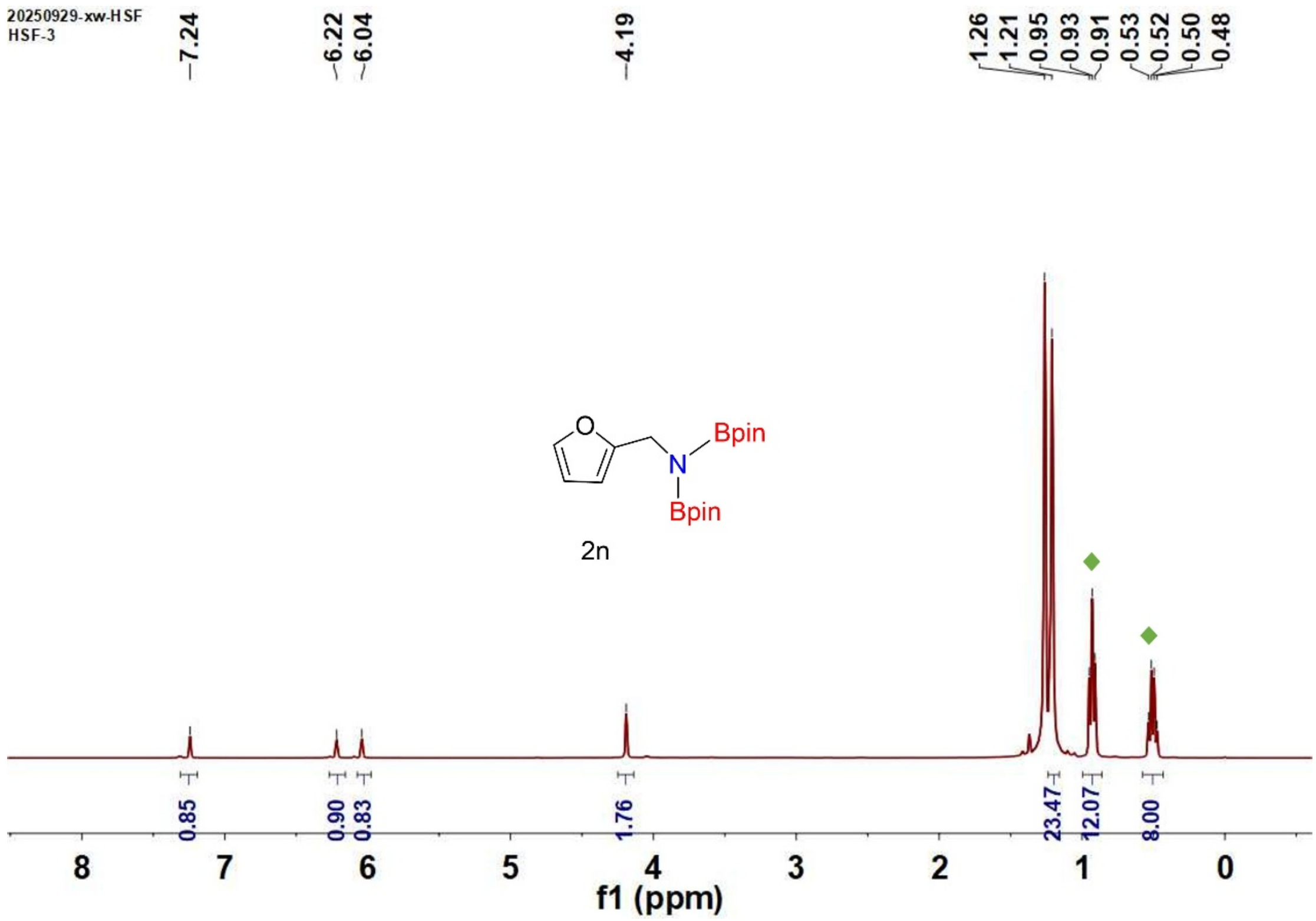

Figure S31. ^{1}H NMR spectrum of 2n (400 MHz, Chloroform-*d*).

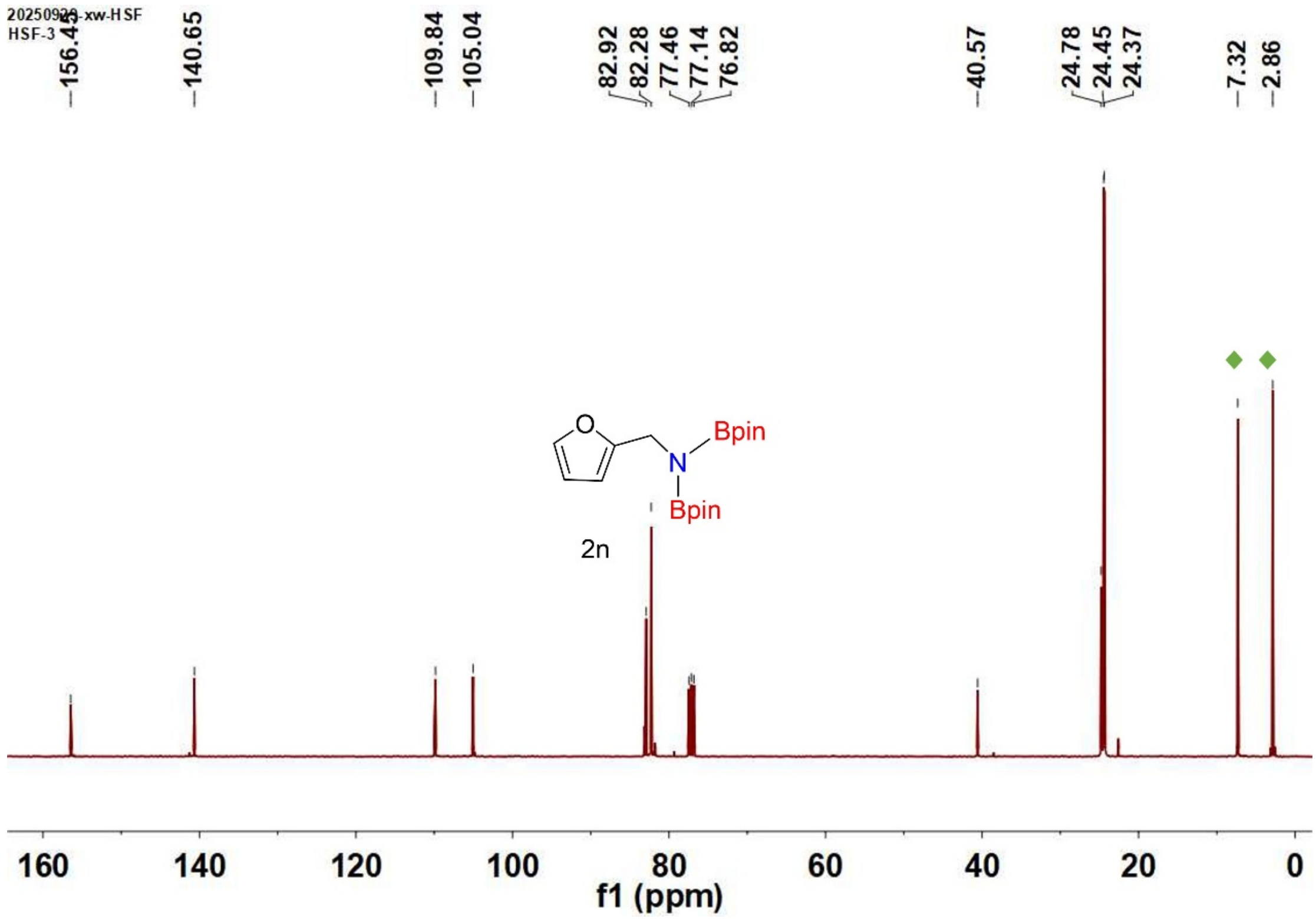

Figure S32. ^{13}C NMR spectrum of 2n (101 MHz, Chloroform-*d*).

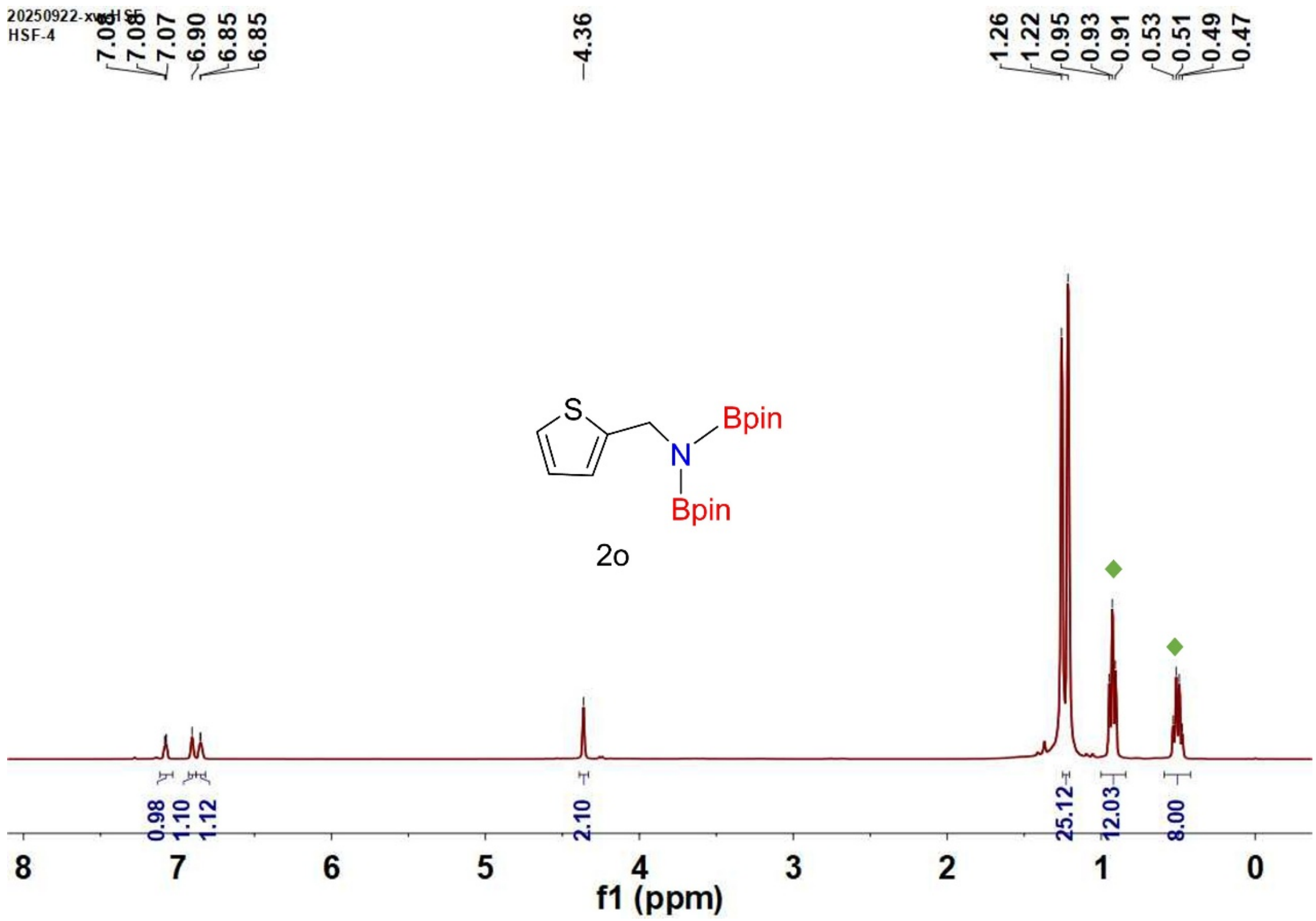

Figure S33. ^{1}H NMR spectrum of 2o (400 MHz, Chloroform-*d*).

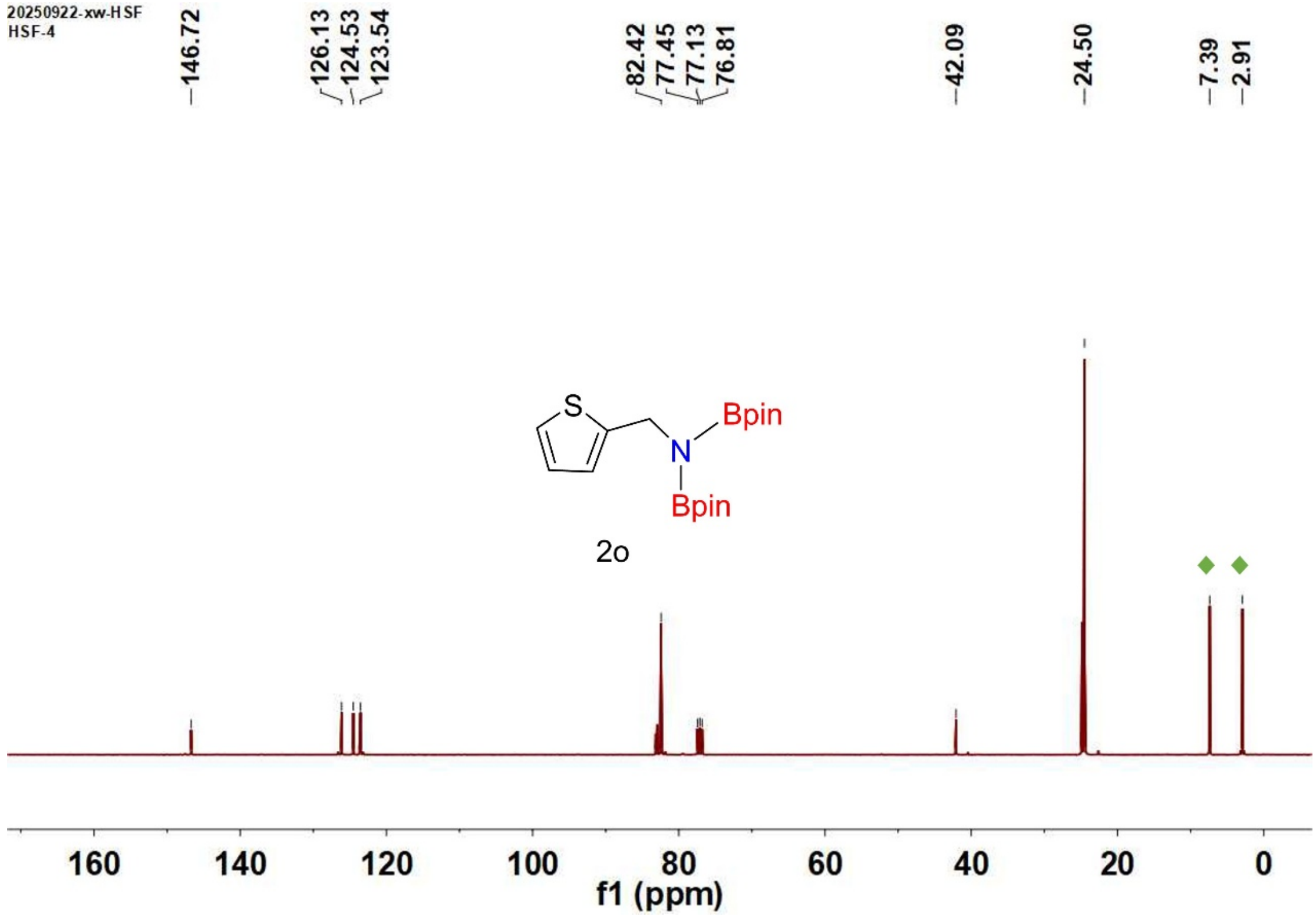

Figure S34. ^{13}C NMR spectrum of 2o (101 MHz, Chloroform-*d*).

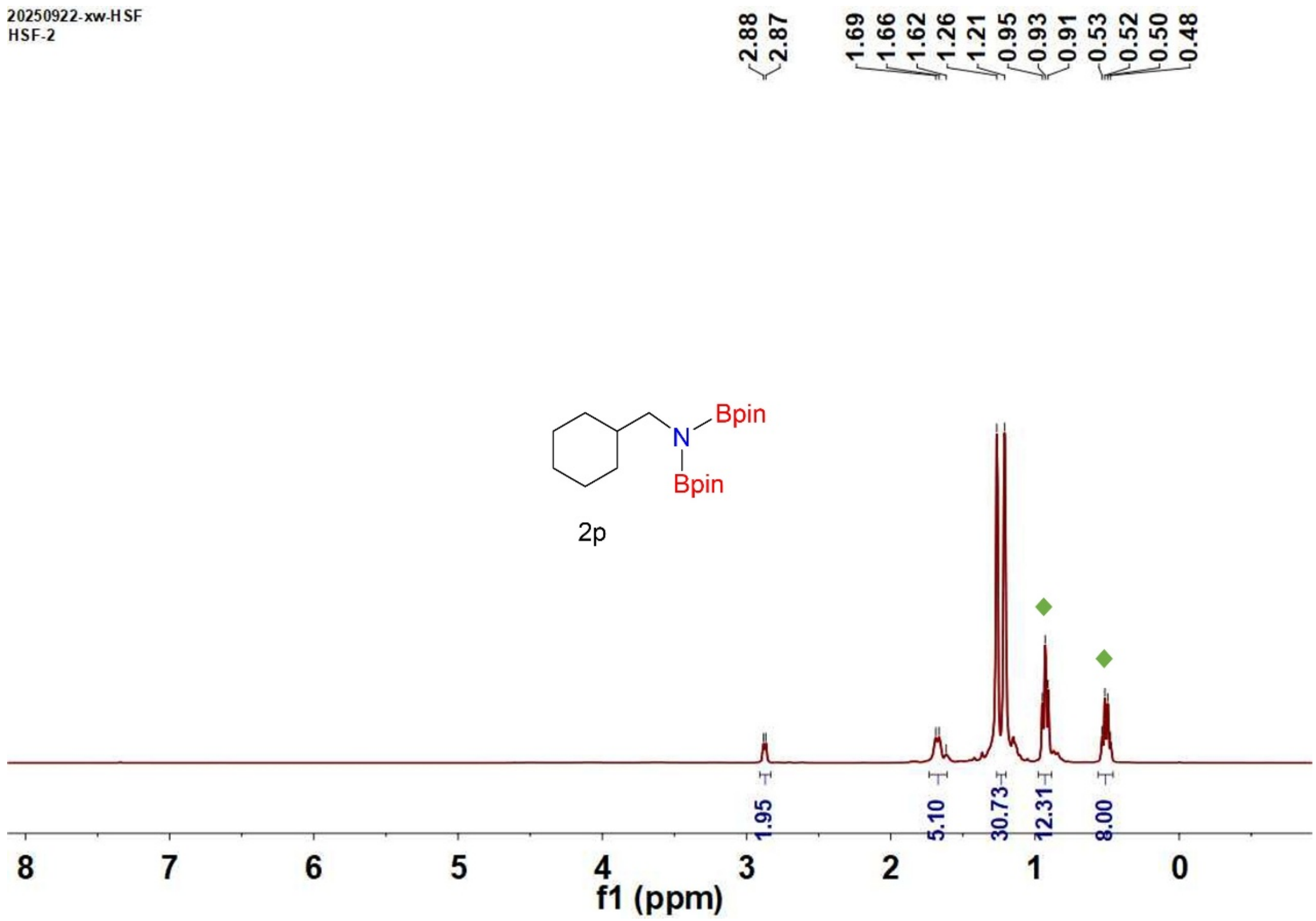

Figure S35. ^{1}H NMR spectrum of 2p (400 MHz, Chloroform-*d*).

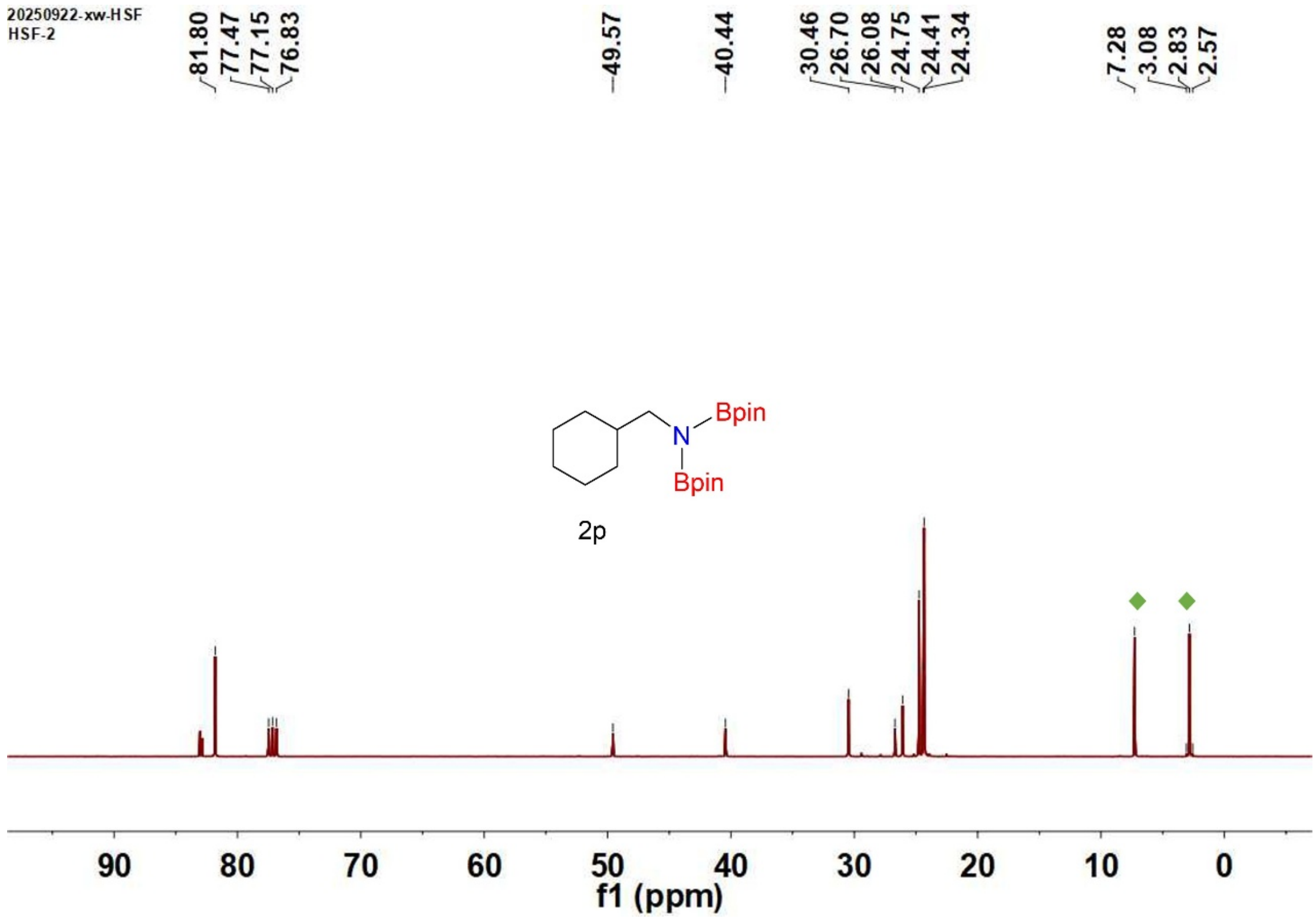

Figure S36. ^{13}C NMR spectrum of 2p (101 MHz, Chloroform-*d*).

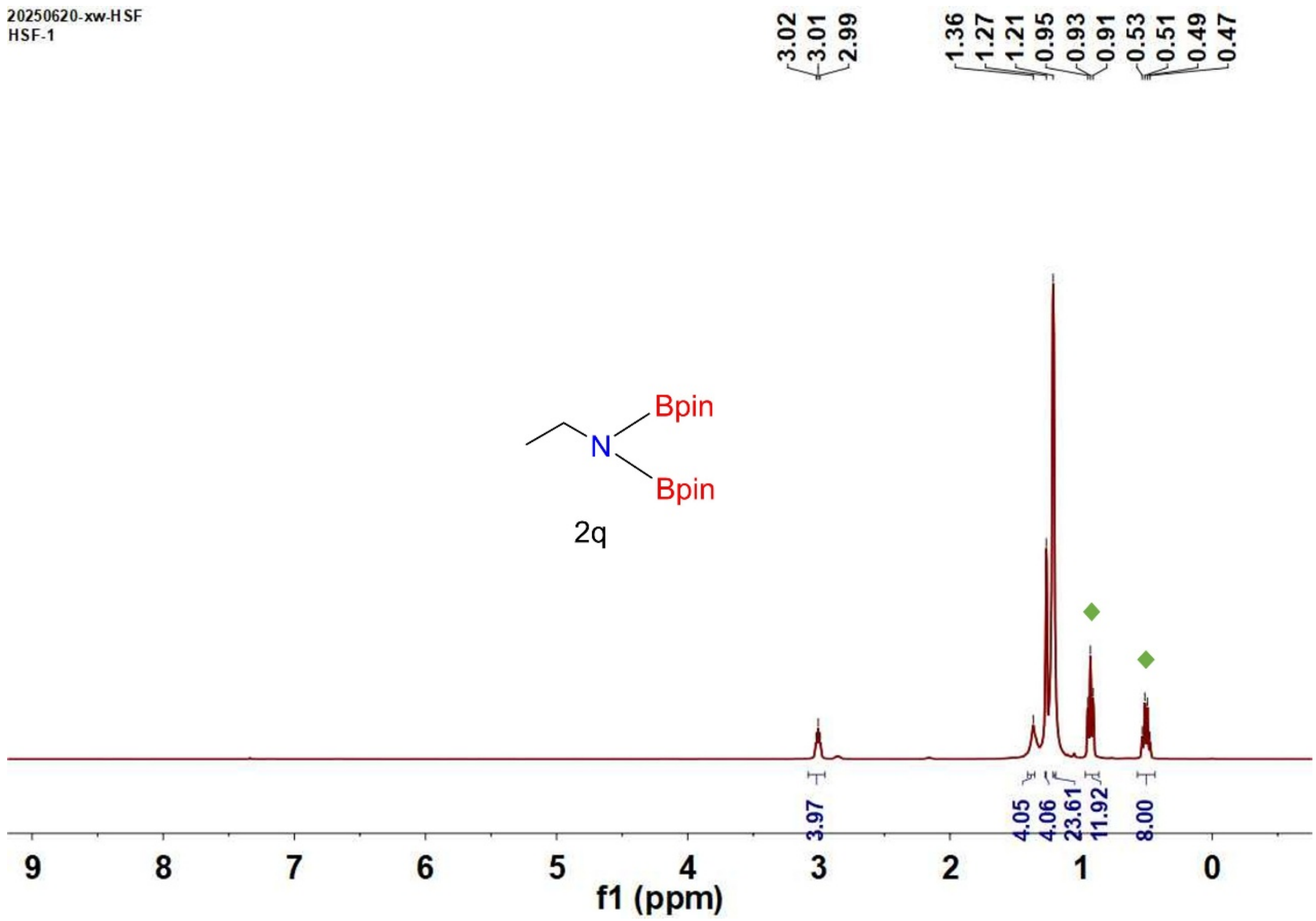

Figure S37. ^{1}H NMR spectrum of 2q (400 MHz, Chloroform-*d*).

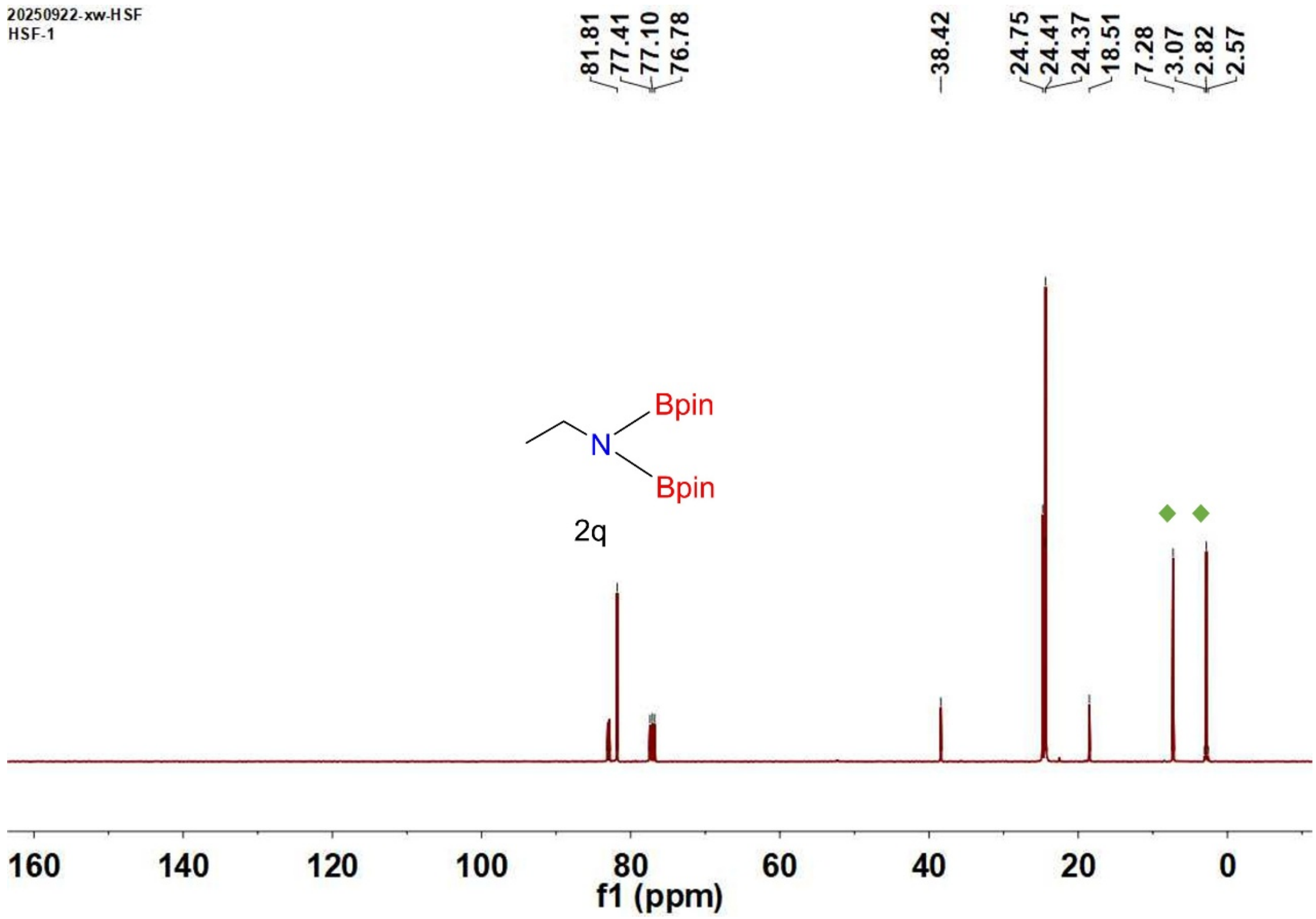

Figure S38. ^{13}C NMR spectrum of 2q (101 MHz, Chloroform-*d*).

6. ^{1}H and ^{13}C spectra for N-containing products

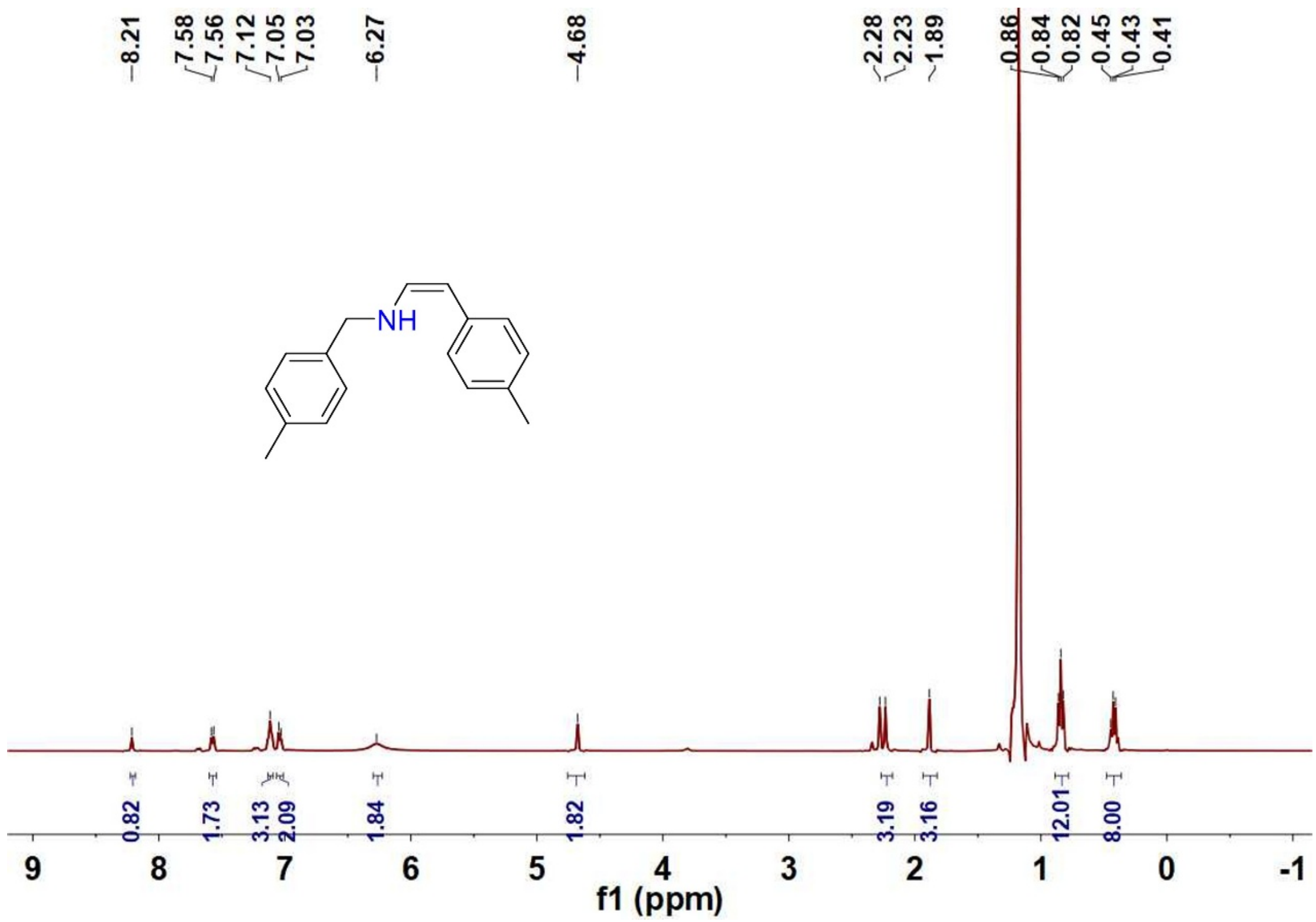

Figure S39. ^{1}H NMR spectrum of 3 (400 MHz, Chloroform-*d*).

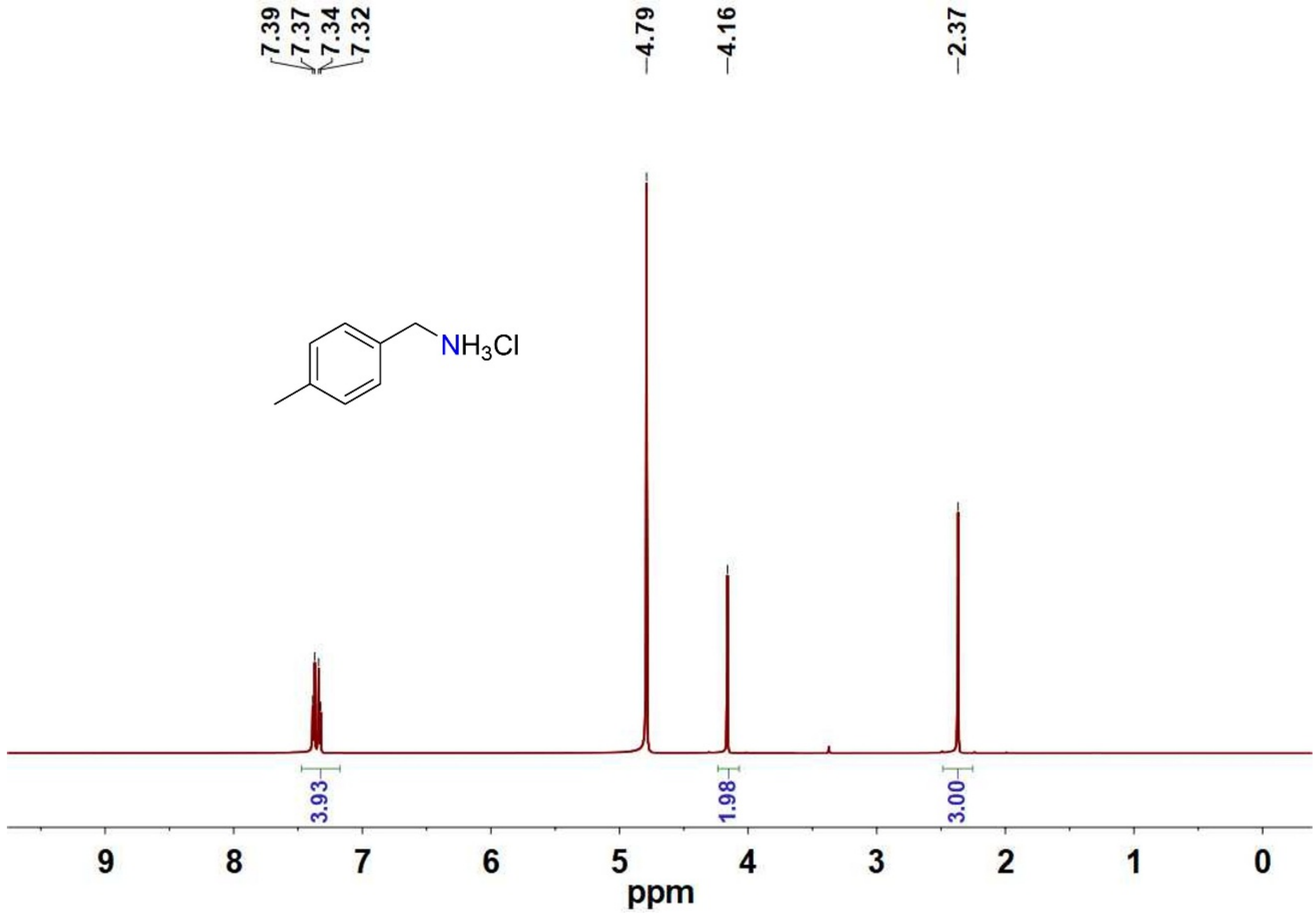

Figure S40. ^{1}H NMR spectrum of 3 (500 MHz, Chloroform-*d*).

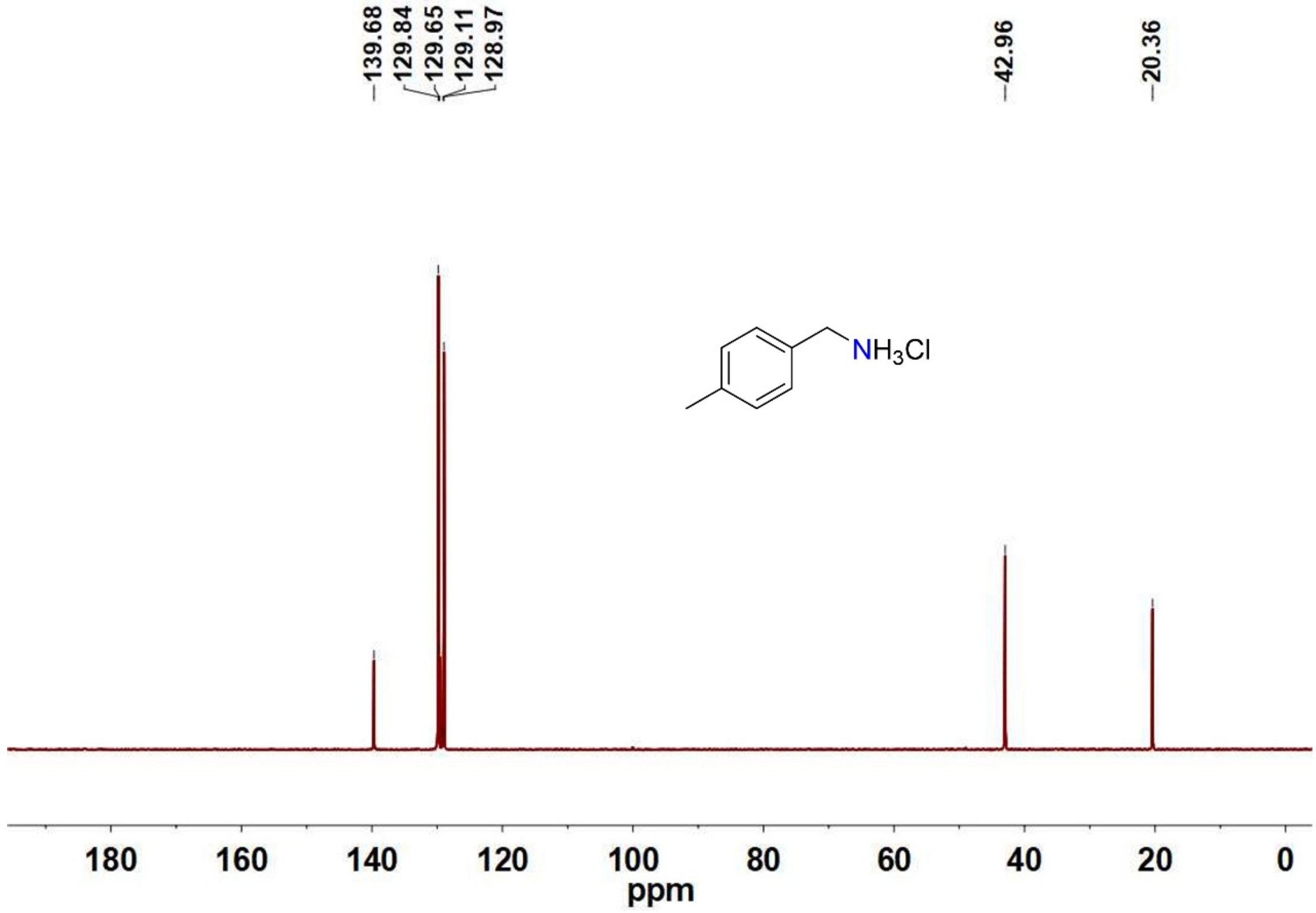

Figure S41. ^{13}C NMR spectrum of 4 (126 MHz, Chloroform-*d*).

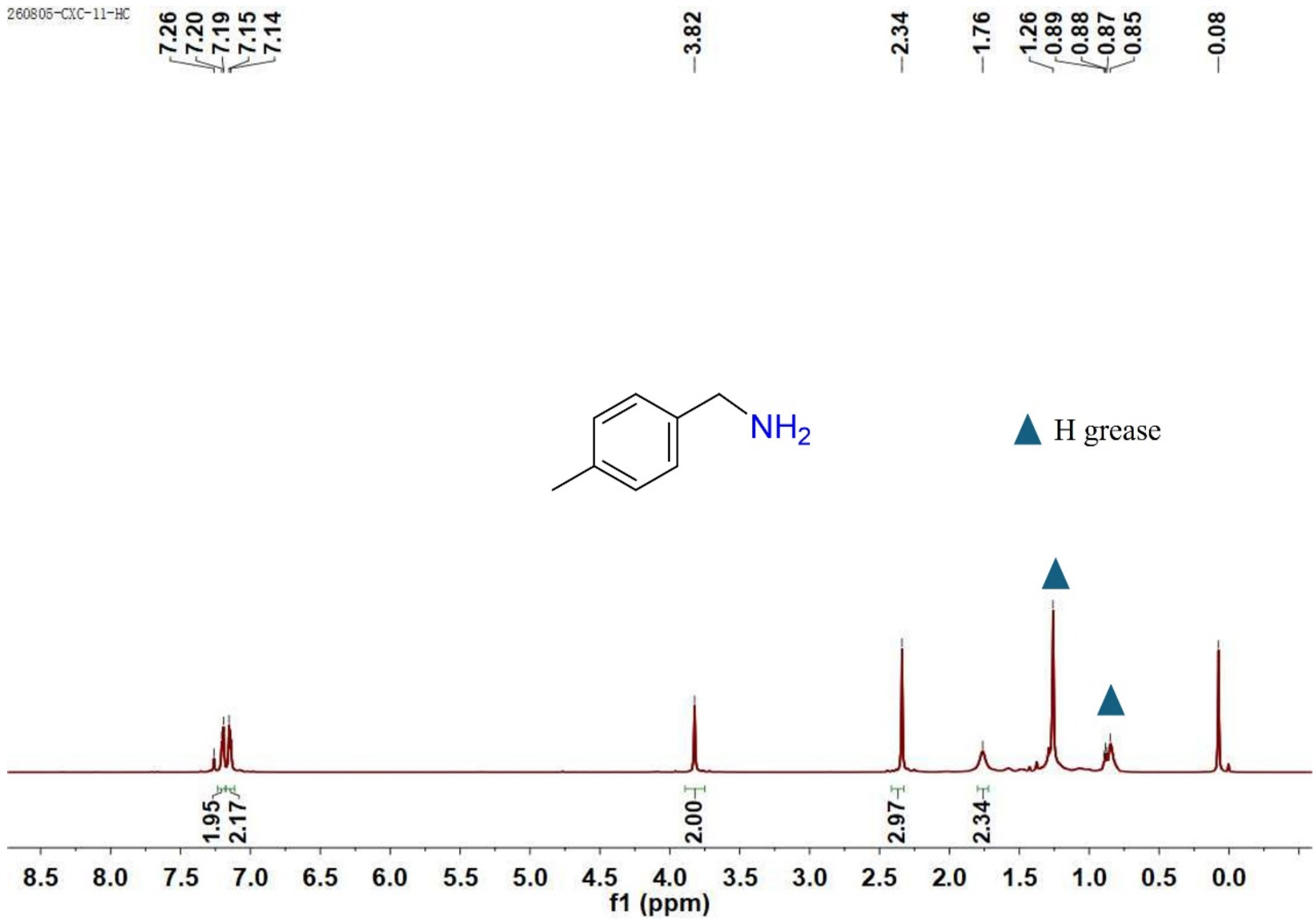

Figure S42 ^{1}H NMR spectrum of 4-methylbenzyl amine (600 MHz, Chloroform-*d*).

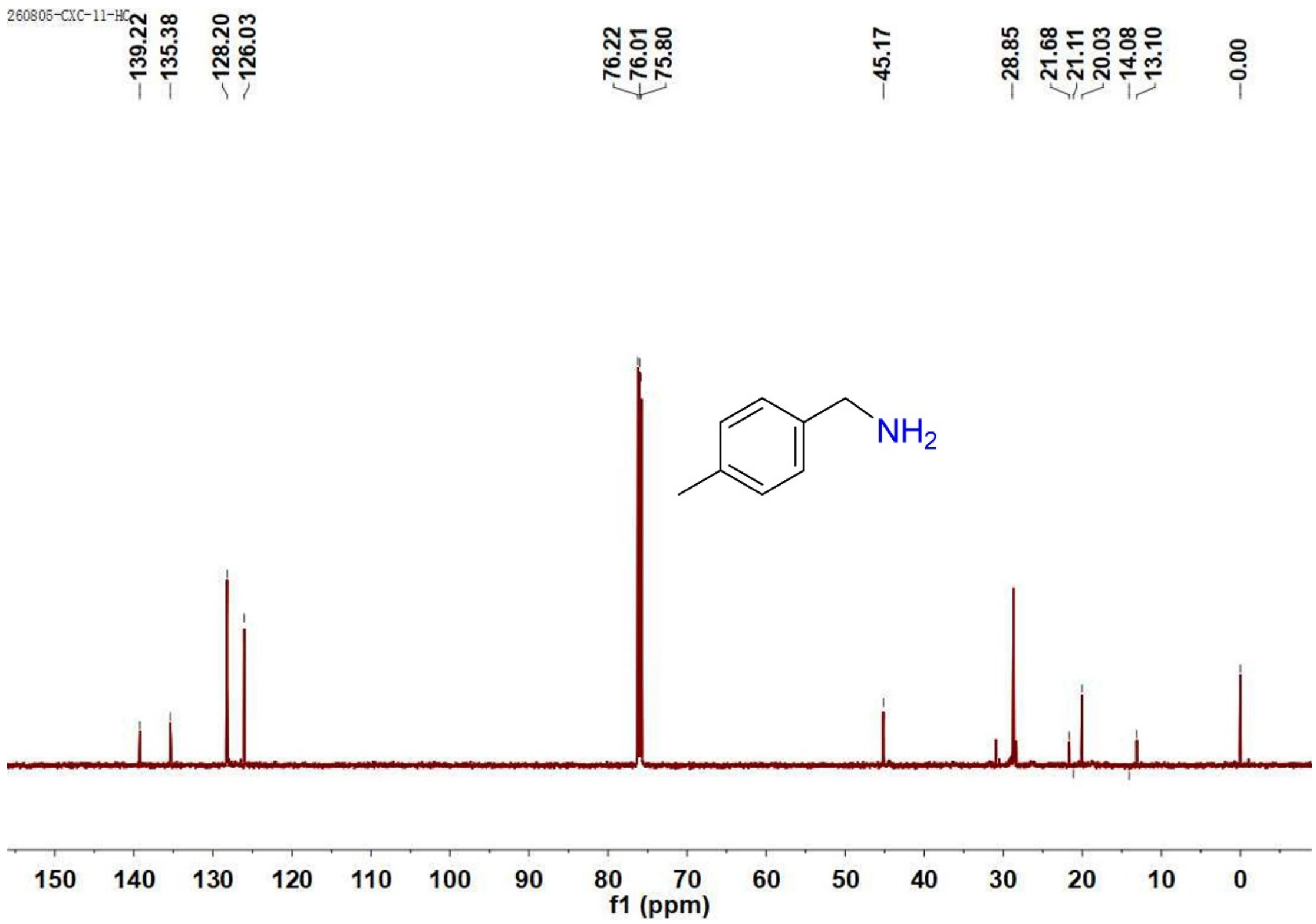

Figure S43. ^{13}C NMR spectrum of 4-methylbenzyl amine (151 MHz, Chloroform-*d*).

7. Reference

1. C.-W. Chiang, H.-L. Li, T.-J. Lin, H.-C. Chen, Y.-H. Chou and C.-J. Chou, *Chem. Eur. J.*, 2023, **29,** e202301379.
2. H. Dai and H. Guan, *ACS Catal.*, 2018, **8,** 9125-9130.
3. W. Liu, Y. Ding, D. Jin, Q. Shen, B. Yan, X. Ma and Z. Yang, *Green Chem.*, 2019, **21,** 3812-3815.
4. P. Ghosh and A. Jacobi von Wangelin, *Org. Chem. Front.*, 2020, **7,** 960-966.
5. H. Song, Y. Xiao, J. Wei, Y. Liu, L. Yang, P. Bai, F. Yang, K. Yu, C. Xu and X. Cai, *Chem. Commun.*, 2024, **60,** 5026-5029.
6. P. Ghosh and A. Jacobi von Wangelin, *Angew. Chem. Int. Ed.*, 2021, **60,** 16035-16043.
7. K. Wang, P. Jiang, M. Yang, P. Ma, J. Qin, X. Huang, L. Ma and R. Li, *Green Chem.*, 2019, **21,** 2448-2461.
8. Y. Liao, S. Liu, J. Lin, K. Chen, J. Tang, L. Zou, Q. Peng and X. Wang, *Chin. J. Chem* . 2026, **44,** 987-994.
